# A Practical Strategy to Discover New Antitumor Compounds by Activating Silent Metabolite Production in Fungi by Diethyl Sulphate Mutagenesis

**DOI:** 10.3390/md12041788

**Published:** 2014-03-27

**Authors:** Shi-Ming Fang, Chang-Jing Wu, Chang-Wei Li, Cheng-Bin Cui

**Affiliations:** 1 Key Laboratory of Structure-Based Drug Design & Discovery of Ministry of Education, School of Traditional Chinese Materia Medica, Shenyang Pharmaceutical University, Shenyang 110016, China; E-Mails: fang_shiming@163.com (S.-M.F.); wucj2009@163.com (C.-J.W.); 2 Beijing Institute of Pharmacology and Toxicology, Beijing 100850, China; E-Mail: sdrlcw@126.com

**Keywords:** natural products, alkaloids, structure elucidation, DES mutagenesis, silent fungal metabolite production

## Abstract

Many fungal biosynthetic pathways are silent in standard culture conditions, and activation of the silent pathways may enable access to new metabolites with antitumor activities. The aim of the present study was to develop a practical strategy for microbial chemists to access silent metabolites in fungi. We demonstrated this strategy using a marine-derived fungus *Penicillium purpurogenum* G59 and a modified diethyl sulphate mutagenesis procedure. Using this strategy, we discovered four new antitumor compounds named penicimutanolone (**1**), penicimutanin A (**2**), penicimutanin B (**3**), and penicimutatin (**4**). Structures of the new compounds were elucidated by spectroscopic methods, especially extensive 2D NMR analysis. Antitumor activities were assayed by the MTT method using human cancer cell lines. Bioassays and HPLC-photodiode array detector (PDAD)-UV and HPLC-electron spray ionization (ESI)-MS analyses were used to estimate the activated secondary metabolite production. Compounds **2** and **3** had novel structures, and **1** was a new compound belonging to a class of very rare natural products from which only four members are so far known. Compounds **1**–**3** inhibited several human cancer cell lines with IC_50_ values lower than 20 μM, and **4** inhibited the cell lines to some extent. These results demonstrated the effectiveness of this strategy to discover new compounds by activating silent fungal metabolic pathways. These discoveries provide rationale for the increased use of chemical mutagenesis strategies in silent fungal metabolite studies.

## 1. Introduction

Natural products are important sources of drugs and drug leads [1,2,3,4]. Among the small molecule antitumor drugs that were approved from the 1930s to the middle of June 2012, 67% were derived from or inspired by natural products [2]. A significant number of natural product drugs and drug leads are of microbial origin, making cultured microbes crucial sources. Fungal metabolites, especially those derived from marine fungi, are particularly important and have attracted considerable attention [5,6,7]. In spite of their roles in drug development, the majority of cultured microbes cannot be used for drug production because the pathways that produce secondary metabolites are silenced in standard culture conditions [8,9,10,11,12]. Various strategies have been developed to activate silenced pathways and elicit metabolite production from microbial isolates. Rational genetic engineering has provided promising new techniques that may reveal additional methods of metabolite production [8,9,10,11,12]. However, current strategies still suffer from significant technical challenges that limit their utility [9]. The complex gene manipulations used in those techniques have also restricted their use in natural products. In contrast, the one strain-many compounds (OSMAC) strategy [13] and chemical epigenetics methodology [14,15] have been widely applied by microbial chemists to access cryptic secondary metabolites [8,9,10,16]. The culture-based, simple procedures outlined by these strategies are suitable for use by microbial chemists. Ribosome engineering is another simple way to activate silent pathways by introducing drug-resistance mutations in bacteria [17,18,19]. This strategy was recently extended to fungi [20]. Additional, simple approaches are needed to activate secondary metabolite production.

Diethyl sulphate (DES), a chemical mutagen, has been used for plant or microbial breeding [21,22,23], but not to activate silent pathways until our group preliminarily tested its use for this purpose [24]. Also, no research reports have systematically discussed the use of chemical mutagenesis to activate silent pathways, though it has been used to synthesize new antibiotics in mutasynthesis [25,26,27], improve antibiotic production [28,29], and investigate antibiotic biosynthesis [30,31]. Due to documented effects of chemical mutagens on the microbial secondary metabolisms [25,26,27,28,29,30,31], we hypothesized that chemical mutagenesis may be more intentionally and rationally used to activate silent pathways for secondary metabolite studies.

**Figure 1:**
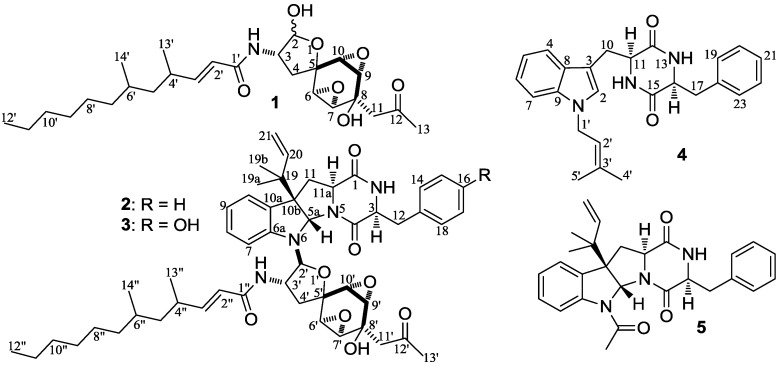
Structures of **1**–**5** produced in mutant BD-1-3 by activating silent metabolites in strain G59.

Several *Penicillium purpurogenum* strains are known to produce novel structural metabolites, including some with antitumor activities [32,33,34,35,36,37]. However, *P. purpurogenum* G59, a marine-derived wild type fungal strain that was initially isolated by our group [38], did not produce any metabolites with antitumor activities in an MTT assay using K562 cells [20,38]. Using this strain, we previously developed a new approach to activate silent metabolite production by introducing drug-resistance with dimethyl sulfoxide (DMSO) as an accessory agent [20]. Our preliminary test on the DES mutagenesis of strain G59 in the presence of DMSO led to the discovery of two new antitumor terpenes [24]. This suggested that the DES mutagenesis of strain G59 may activate additional silent secondary metabolite production. Therefore, we used DES mutagenesis of strain G59 and an old, modified method of chemical mutagenesis in the present study to test our hypothesis and attempt to discover additional new compounds. Four new antitumor compounds, **1**–**4** (Figure 1), were discovered by activating silent metabolic pathways in the G59 strain by DES mutagenesis in the present study. Among these, **1** was a new compound belonging to a rare family of natural products with only four members known from natural sources, and **2** and **3** were novel structures. We report herein the use of DES chemical mutagenesis to activate silent fungal secondary metabolites and the discovery of four new compounds by the use of this strategy.

## 2. Results and Discussions

### 2.1. Modification of the DES Mutagenesis Procedure

We reported that the treatment of *P. purpurogenum* G59 spores at 4 °C with aqueous DMSO alone did not affect strain growth [20]. As DMSO may enhance DES penetration, we preliminarily treated the G59 spores in 20% (v/v) DMSO with 0.5% (v/v) DES at 4 °C for 1 day and got an antitumor mutant [24]. As an additional preliminary test in the present study, the G59 spores were treated in 50% (v/v) DMSO with 0.5% (v/v) DES at 4 °C for 1 day, which gave another antitumor mutant. Thus, the DES mutagenesis in the present study was performed on the G59 spores in aqueous DMSO at 4 °C and groups for the spore treatments were set as shown in Table 1.

### 2.2. DES Mutagenesis and Mutant Selection

The DES mutagenesis of the strain G59 was performed on fresh G59 spores. The fresh G59 spores were suspended in 20% or 50% (v/v) aqueous DMSO and treated with 0.5%, 1.0%, and 2.0% (v/v) DES at 4 °C for 1–30 days. Aliquots (80 µL) of the treated spores were spread on potato dextrose agar (PDA) plates at 1, 2, 7, 10, 15, and 30 days of the treatment and incubated at 28 °C for 5–7 days. During the incubation period, mutant colonies with different morphological characteristics were selected by single colony isolation. Typical phenotypic differences of the parent G59 strain and several mutants are given as examples in Figure S1 in the Supplementary Information (SI). A total of 42 mutants were selected, including 22 from the 20% DMSO group and 20 from the 50% DMSO group (Table 2).

**Table 1:** Group setting for G59 spore treatments with diethyl sulphate (DES) in aqueous dimethyl sulfoxide (DMSO) suspensions.

Groups	DES% (v/v)	Treatment Times at 4 °C	Dilution Ratio ^a^
20% (v/v) DMSO	0.5, 1, 2	1–30 days	OSS:DMSO = 4:1
50% (v/v) DMSO	0.5, 1, 2	1–30 days	OSS:DMSO = 1:1

^a^ Original spore suspensions at the same spore density in water were diluted with fresh DMSO to the given ratio, so that the spore density remained the same in 20% and 50% DMSO groups, respectively, details see in Experimental Section, OSS indicates the original G59 spore suspension in water.

**Table 2:** Mutant selection by DES-treatment of G59 spores in aqueous DMSO at 4 °C ^a^.

DMSO% (v/v)	DES% (v/v)	Numbers of Mutants Selected on the Days of DES-Treatment at 4 °C						
		1st Day	2nd Day	7th Day	10th Day	15th Day	30th Day	Total
20%	0.5	2	1	2	2	1	1	9
	1	1	2	1	2	NC	NC	6
	2	1	1	1	2	1	1	7
	Sum	4	4	4	6	2	2	22
50%	0.5	2	2	NC	2	1	NC	7
	1	2	2	1	3	1	NC	9
	2	2	NC	1	1	NC	NC	4
	Sum	6	4	2	6	2	0	20
Total		10	8	6	12	4	2	42

^a^ Fresh G59 spores were treated with DES in aqueous DMSO at 4 °C for different times (day). During the treatment period, aliquots (80 μL) of the treated spores were spread on PDA plates, incubated at 28 °C for 5–7 days, and colonies with different appearances were selected by single colony isolation to obtain the aimed mutants; NC: No colonies developed.

The mutagenicity of DES correlates with its action as a DNA ethylating agent [39,40]. Higher levels of DES lead to higher rates of mutation, but hydrolysis decreases DES levels, which is enhanced at higher temperatures [39]. In the present study, the mutagenesis of G59 spores in 20% and 50% DMSO was performed with DES at 4 °C. The low temperature was used to limit the hydrolysis of DES and lower the toxicity of DMSO, which is also enhanced at higher temperatures [20]. Previously, we confirmed that the treatment of G59 spores with 20% and 50% DMSO at 4 °C did not affect the strain growth [20]. DMSO increases membrane permeability and enhances the penetration of hydrophilic and hydrophobic molecules into cells. DMSO was used to increase the penetration of DES into G59 cells and to maintain high levels of intracellular DES. DMSO might thus have contributed to the DES mutagenesis of the G59 strain. Bioactive mutants that have acquired metabolic capability to produce secondary metabolites with antitumor activities by the DES mutagenesis were obtained in a quite high efficiency as shown below. The role of DMSO in this phenomenon was unclear and requires further investigation.

### 2.3. Estimation of Activated Production of Silent Metabolites by DES Mutagenesis by Bioassays

In order to preliminarily estimate the activated production of silent bioactive metabolites by DES mutagenesis, we tested antitumor activities of the extracts of strain G59 and the 42 mutant cultures. The mutants and the control G59 strain were fermented at the same time and same conditions to obtain their ethyl acetate (EtOAc) extracts. Then, the extracts were subjected to the MTT assay using K562 cells to evaluate their antitumor activities. Extracts of 31 mutants, 73.8% of the 42 mutants, inhibited the K562 cells with the inhibition rate (IR%) values in Table 3. Among them, 15 were from the 20% DMSO group, which accounted for 68.2% of the 22 mutants in this group, and 16 were from the 50% DMSO group, which accounted for 80.0% of the 20 mutants in this group. In contrast, the G59 extract did not inhibit the K562 cells at 100 µg/mL (Table 3), similar to our previous results at 100 and 1000 µg/mL [20,24,38]. The 31 mutants have acquired the metabolic capability to produce antitumor metabolites through DES mutagenesis.

### 2.4. Chromatographic Analysis of Metabolite Production Induced by DES Mutagenesis

To further examine that the secondary metabolite production was activated by DES mutagenesis, the EtOAc extracts of the control G59 strain and the 31 mutants were subjected to HPLC-photodiode array detector (PDAD)-UV and HPLC-electron spray ionization (ESI)-MS analyses. The control G59 strain and the 31 mutant extracts produced different HPLC profiles, and many new peaks were detected in the mutant extracts (Figures S2 and S3 in the SI for the HPLC-PDAD-UV and HPLC-ESI-MS analyses, respectively). New peaks in the mutant extracts were verified by their UV (Figures S2 in the SI) and MS (Figures S3 in the SI) spectra, and these analyses indicated that diverse secondary metabolites were being produced by these mutants. These results, coupled with the bioassay results, indicated that some of the biosynthetic pathways originally silent in the G59 strain were activated to produce bioactive secondary metabolites in these mutants.

### 2.5. Isolation of 1–5 in the Mutant BD-1-3 Extract and Identification of the Known Compound 5

Large-scale fermentation and extraction of the bioactive mutant BD-1-3 gave an EtOAc extract that inhibited K562 cells with an IR% value of 68.7% at 100 µg/mL. However, the control G59 extract that was obtained by fermentation of the G59 strain at the same time and same conditions did not inhibit the K562 cells. Repeated column chromatography of the mutant extract, tracing newly produced metabolites by direct comparison with the control G59 extract, afforded crude **1**–**5** samples. Further purification of the crude samples by HPLC yielded five pure metabolites **1**–**5** (Figure 1). Structures of four new compounds, named penicimutanolone (**1**), penicimutanin A (**2**), penicimutanin B (**3**), and penicimutatin (**4**), were elucidated by spectroscopic methods; the structure elucidation of **1**–**4** is described in detail in Section 2.7. The known compound **5** was identified as fructigenine A [41] on the basis of its physicochemical and spectroscopic data.

**Table 3:** MTT assay results for the G59 and 31 mutant extracts on K562 cells ^a^.

Strain	Condition for Treating G59 Spores to Select Mutant			IR% at 100 µg/mL(Mean ± SD, *n* = 3)
	DMSO% (v/v)	DES% (v/v)	Time (day)	
G59	—	—	—	5.6 ± 3.5
BD-1-1	20	0.5	10	43.7 ± 5.4
BD-1-3		0.5	1	58.1 ± 3.6
BD-1-5		0.5	2	46.6 ± 18.8
BD-1-5′		1.0	10	43.9 ± 7.4
BD-1-6		0.5	7	49.0 ± 14.1
BD-2-5		1.0	7	44.3 ± 17.1
BD-3-1		2.0	1	55.8 ± 2.0
BD-3-5		2.0	7	32.6 ± 20.2
BD-1m-1		0.5	10	43.9 ± 25.0
BD-2m-2		1.0	10	47.0 ± 12.8
BD-3m-1		2.0	10	40.8 ± 11.1
BD-3m-2		2.0	10	38.3 ± 15.9
BD-1n-1		0.5	15	65.5 ± 20.5
BD-3n-1		2.0	15	55.5 ± 9.3
BD-3p-1		2.0	30	52.2 ± 11.6
AD-1-1	50	0.5	1	38.7 ± 17.5
AD-1-2		0.5	1	47.4 ± 23.9
AD-1-5		0.5	1	44.9 ± 23.1
AD-2-1		1.0	1	49.8 ± 17.9
AD-2-2		1.0	1	44.7 ± 21.8
AD-2-3		1.0	2	37.3 ± 23.8
AD-2-4		1.0	2	38.5 ± 25.0
AD-2-5		0.5	1	27.9 ± 15.5
AD-1m-1		1.0	1	37.3 ± 9.1
AD-1m-2		0.5	10	41.3 ± 27.9
AD-2m-1		1.0	1	36.4 ± 7.7
AD-2m-2		1.0	2	47.6 ± 1.9
AD-2m-3		1.0	10	44.2 ± 29.7
AD-3m-1		2.0	10	53.7 ± 5.1
AD-1n-1		0.5	15	52.9 ± 13.2
AD-2n-1		1.0	2	40.4 ± 1.8

^a^ The IR% values given in this table were from the triplicate MTT tests that were carried out using the EtOAc extracts from three rounds of individual fermentations of the parent G59 strain and 31 mutants, respectively.

### 2.6. Experimental Verification for the Newly Produced 1–5 in the Mutant BD-1-3 Extract

The EtOAc extracts of the control G59 strain and the mutant BD-1-3 were subjected to HPLC-PDAD-UV and HPLC-ESI-MS analyses using **1**–**5** as references. In the HPLC-PDAD-UV analysis, all of **1**–**5** were detected in the BD-1-3 extract, though **3** and **4** were very minor metabolites of the mutant (Figure S4 in the SI). This was also supported by the HPLC-ESI-MS analysis (Figure S5 in the SI). In contrast, none of these metabolites were detected in the control G59 extract both by the HPLC-PDAD-UV (Figure S4 in the SI) and HPLC-ESI-MS (Figures S5 in the SI) analyses. These analyses indicated that the production of **1**–**5** in the mutant BD-1-3 was caused by the activation of silent metabolic pathways in the strain G59 by DES mutagenesis. Although the silent pathways remain undefined, the structural features of **1**–**5** suggested that they may originate from the polyketide synthase (PKS) system (**1**), the mixed nonribosomal peptide synthetase (NRPS)-PKS systems (**2** and **3)**, and the NRPS systems involving prenyltransferases (**4** and **5**). Additional investigation into the activated metabolic pathways and their regulatory mechanisms are needed for further exploration of the affected pathways.

### 2.7. Structure Elucidation of New Compounds 1–4

Penicimutanolone (**1**), a crystalline powder (MeOH), mp 114–115 °C, 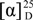
−11.9 (*c* 1.0, MeOH), had the molecular formula C_26_H_41_NO_7_ by HRESIMS. The UV spectrum showed an end absorption at 215 nm (log ε 4.21) in MeOH, and the IR spectrum showed absorptions due to OH, CH_3_/CH_2_, C=O, NHCO, and C=C groups (see the IR spectrum in the SI). The NMR spectra gave two sets of ^1^H/^13^C signals in approximate ratios of 6.3:1 in CDCl_3_ and 1.8:1 in CD_3_OD. The ratio in CDCl_3_ changed from approximate 5.5:1 in the ^1^H-NMR spectrum measured soon after dissolving **1** in CDCl_3_ to the approximate ratio of 6.3:1 in the ^1^H-NMR spectrum measured after over a week in the CDCl_3_ solution, which appeared along with disappearance of two hydroxyl proton signals. These observations revealed the presence of dynamic isomerism in the solutions, which made the NMR spectra complicated, especially the ^1^H-NMR spectrum. Exhaustive analyses of the ^1^H and ^13^C-NMR spectra with the aid of DEPT, GOESY, ^1^H-^1^H COSY, HMQC, HMBC, and NOESY techniques resulted in the full signal assignments for the major isomer **1a** and the miner isomer **1b** both in CDCl_3_ (Table 4 and Table S1 in the SI) and CD_3_OD (Table S2 in the SI).

Partial structures related to the proton spin systems in the two isomers **1a** and **1b** were determined by DEPT, ^1^H-^1^H COSY, and HMQC spectra. Then, all structural elements were connected by HMBC correlations to deduce planar structures. The 1-oxaspiro[4,5]decane ring system with a cyclohexane bisoxirane moiety was established by the HMBCs: The HMBCs were detected on H-2/C-5, Hα-4/C-5, H_2_-4/C-6, H_2_-4/C-10, H-6/C-5, H-7/C-5, H-9/C-5, H-10/C-5, H-7/C-8, and H-9/C-8. Amide linkage of the acyclic fatty acid chain was located at C-3 by the HMBCs on HN/C-2,3,1′,2′ and H-3/C-1′. The HMBCs on HO-8/C-7,8,9,11, H_2_-11/C-7,8,9,12,13, and H_3_-13/C-11,12 joined both 8-CH_2_COCH_3_ and 8-OH to C-8. The NOEs on the protons HO-2/H-2 linked 2-OH to C-2 to form a hemiacetal. This hemiacetal structure caused *cis*-*trans* isomerism in solution.

**Figure 2:**
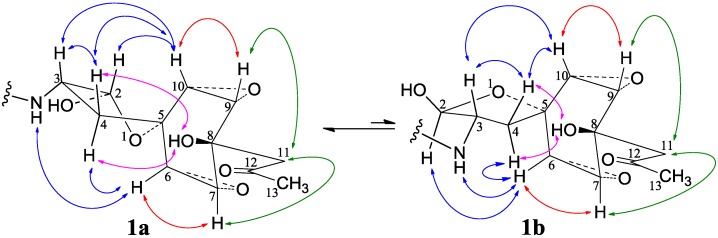
Relative stereochemistry of the spiro ring system for 2,3-*cis* and -*trans* isomers **1a** and **1b**. Key NOE interactions are indicated by arrows.

The major isomer **1a** and the minor isomer **1b** were determined as 2,3-*cis* and 2,3-*trans* isomers by the NOEs shown in Figure 2, respectively. These NOEs also established the relative stereochemistry of the spiro ring system in **1** as shown in Figure 2. Couplings (Table 4) of vicinal protons on five-membered rings consisted of this ring conformation [42,43]. Large four-bond couplings of the epoxide protons H-6/H-10 (3.0 Hz for **1a**) and H-7/H-9 (3.4 Hz for **1a**) also coincided with the boat conformation of the cyclohexane ring as seen in aranorosine [42,43]. Further, the NOEs on H_2_-4/HO-8 defined the *quasi*-axial 8-OH on the same side of C-4, while the NOEs on H_2_-11/H-7 and H_2_-11/H-9 conferred the *quasi*-equatorial 8-CH_2_COCH_3_ in **1** (Figure 2). The 15.3 Hz coupling of H-2′/H-3′ and the NOEs on H-2′/H-4′ and H-2′/H_3_-13′ indicated the *E* configuration of the 2′,3′-double bond in **1**.

**Table 4:** 600 MHz ^1^H and 150 MHz ^13^C-NMR data of **1** in CDCl_3_ (data from the spectra measured after over a week in CDCl_3_, **1a**:**1b** = approx. 6.3:1) ^a^.

**No.**	**1a** (2,3-*cis*)						**1b** (2,3-*trans*)								
	**δ**_C_^b,c^	**δ**_H_^b^ (*J* **in Hz**)	**COSY** ^d^	**NOE** ^e^	**HMBC** ^f^	**δ**_C_^b,c^	**δ**_H_^b^ (*J* **in Hz**)	**COSY** ^d^	**NOE** ^e^	**HMBC** ^f^
2	96.45 s	5.55 t (4.3)	H-3, NH, Hα-4	2-OH, H-3,10, NH, Hβ-4	C-3,4,5	102.61 s	5.57 br d (3.7)	H-3, Hα-4	2-OH, H-3,6, NH, Hα-4	C-3,4,5
2-OH	—	4.98 g br s		H-2		—	4.80 ^g^ br s		H-2	
3	52.11 d	4.72 dtd (10.7, 8.4, 4.3)	H-2, NH, H_2_-4	H-2, NH, Hβ-4, H-10	C-2,4,1′	57.21 d	4.59 m	H-2, NH, H_2_-4	H-2, NH, Hβ-4, H-10	C-2,5,1′
NH	—	6.06 br d (8.0)	H-2, H-3	H-2,3,6, Hα,β-4, H-2′	C-2,3,1′,2′	—	5.95 br s	H-3	H-2,3,6, Hα,β-4, H-2′	
4	37.06 t	Hβ 2.58 dd (12.9, 8.4) Hα 2.00 ddd (12.9, 10.7, 4.3)	H-3, Hα-4 H-2, H-3, Hβ-4	H-2,3, NH, Hα-4, H-10 NH, Hβ-4, H-6	C-2,3,6,10 C-3,5,6,10	38.50 t	Hβ 2.63 br dd (12.5, 6.2) Hα 2.16 br d (12.5)	H-3, Hα-4 H-2, H-3, Hβ-4	H-3, NH, Hα-4, H-10 H-2, NH, Hβ-4, H-6	C-2,3,5,6,10 C-2,3,6,10
5	79.05 s	—	—	—	—	80.45 s	—	—	—	—
6	59.37 d	3.31 dd (3.2, 2.6)	H-7, H-10	NH, Hα-4	C-4,5,7,10	59.55 d	3.36 br s	H-7, H-10	H-2, NH, Hα-4	C-7,10
7	58.63 d	3.25 dd (3.3, 2.5)	H-6, H-9	H_2_-11	C-5,6,8,9,11	58.63 d	3.25 dd (3.2, 2.6)	H-6, H-9	H_2_-11	C-6,8,9
8	66.37 s	—	—	—	—	66.32 s	—	—	—	—
8-OH	—	4.67 ^g^ br s		H_2_-11	C-7,8,9,11	—	4.67 ^g^ br s		H_2_-11	C-7,8,9,11
9	58.08 d	3.27–3.23 m	H-7, H-10	H_2_-11	C-5,7,8,10,11	58.29 d	3.27-3.23 m	H-7, H-10	H_2_-11	C-7,8,10
10	57.42 d	3.19 dd (3.2, 2.5)	H-6, H-9	H-2, H-3, Hβ-4	C-4,5,6,9	57.81 d	3.16 br s	H-6, H-9	H-3, Hβ-4	C-6,9
11	47.29 t	Ha 3.09 d (18.1) Hb 3.05 d (18.1)	H_3_-13 H_3_-13	H-7,9, HO-8, H_3_-13 H-7,9, HO-8, H_3_-13	C-7,8,9,12 C-7,8,9,12	47.33 t	Ha 3.09 d (18.1) Hb 3.05 d (18.1)	H_3_-13 H_3_-13	H-7,9, HO-8, H_3_-13 H-7,9, HO-8, H_3_-13	C-7,8,9,12 C-7,8,9,12
12	210.89 s	—	—	—	—	210.86 s	—	—	—	—
13	31.65 q	3H 2.28 s	H_2_-11	H_2_-11	C-11,12	31.67 q	3H 2.26 s	H_2_-11	H_2_-11	C-11,12
1′	166.12 s	—	—	—	—	166.16 s	—	—	—	—
2′	121.41 d	5.76 d (15.3)	H-3′, H-4′	NH, H-4′, H_3_-13′	C-1′,3′,4′	121.19 d	5.74 d (15.0)	H-3′, H-4′	NH, H-4′, H_3_-13′	C-1′,4′
3′	151.30 d	6.73 br dd (15.3, 8.3)	H-2′, H-4′, H_3_-13′	H-4′, Ha-5′, H_3_-13′	C-1′,2′,4′,5′,13′	151.67 d	6.74 br dd (15.0, 8.0)	H-2′, H-4′, H_3_-13′	H-4′, Ha-5′, H_3_-13′	C-1′,2′,4′,5′,13′
4′	34.13 d	2.44–2.33 m	H-2′,3′, H_2_-5′, H_3_-13′	H-2′,3′, H_2_-5′, H_3_-13′,14′	C-2′,3′,5′,6′,13′	34.11 d	2.12-2.04 m	H-2′,3′, H_2_-5′,H_3_-13′	H-2′,3′, H_2_-5′, H_3_-13′, 14′	C-2′,3′,5′,6′,13′
5′	44.05 t	Ha 1.39–1.33 m Hb 1.14–1.07 m	H-4′, Hb-5′, H-6′ H-4′, Ha-5′, H-6′	H-4′ H-4′	C-3′,4′,6′,7′,13′,14′ C-3′,4′,6′,7′,13′,14′	44.05 t	Ha 1.39–1.33 m Hb 1.14–1.07 m	H-4′, Hb-5′, H-6′ H-4′, Ha-5′, H-6′	H-4′ H-4′	C-3′,4′,6′,7′,13′,14′ C-3′,4′,6′,7′,13′,14′
6′	30.45 d	1.44‒1.36 m	H_2_-5′, H_2_-7′, H_3_-14′		C-4′,5′,7′,8′,14′	30.45 d	1.44‒1.36 m	H_2_-5′, H_2_-7′, H_3_-14′		C-4′,5′,7′,8′,14′
7′	37.42 t	Ha 1.27–1.18 m Hb 1.11–1.05 m	H-6′, Hb-7′, H_2_-8′ H-6′, Ha-7′, H_2_-8′		C-5′,6′,8′,9′,14′ C-5′,6′,8′,9′,14′	37.38 t	Ha 1.27–1.18 m Ha 1.11–1.05 m	H-6′, Hb-7′, H_2_-8′ H-6′, Ha-7′, H_2_-8′		C-5′,6′,8′,9′,14′ C-5′,6′,8′,9′,14′
8′	26.84 t	2H 1.28–1.21 m	H_2_-7′, H_2_-9′		C-6′,7′,9′,10′	26.84 t	2H 1.28–1.21 m	H_2_-7′, H_2_-9′		C-6′,7′,9′,10′
9′	29.71 t	2H 1.27-1.18 m	H_2_-8′, H_2_-10′		C-7′,8′,10′,11′	29.71 t	2H 1.27–1.18 m	H_2_-8′, H_2_-10′		C-7′,8′,10′,11′
10′	31.93 t	2H 1.28–1.21 m	H_2_-9′, H_2_-11′		C-8′,9′,11′,12′	31.93 t	2H 1.28–1.21 m	H_2_-9′, H_2_-11′		C-8′,9′,11′,12′
11′	22.67 t	2H 1.31–1.26 m	H_2_-10′, H_3_-12′		C-9′,10′,12′	22.67 t	2H 1.32–1.28 m	H_2_-10′, H_3_-12′		C-9′,10′,12′
12′	14.08 q	0.88 t (7.0)	H_2_-11′		C-10′,11′	14.08 q	0.83 t (7.0)	H_2_-11′		C-10′,11′
13′	20.47 q	1.03 dd (6.6, 0.8)	H-3′, H-4′	H-2′,3′, 4′	C-3′,4′,5′	20.43 q	1.01 d (6.6)	H-4′	H-2′,3′, 4′	C-3′,4′,5′
14′	19.56 q	0.84 d (6.4)	H-6′	H-4′	C-5′,6′,7′	20.41 q	0.82 d (6.4)	H-6′	H-4′	C-5′,6′,7′

^a^ Signals were assigned on the basis of DEPT, GOESY 1D difference NOE, 2D ^1^H–^1^H COSY, HMQC, HMBC, and NOESY experiments. The approximate 6.3:1 ratio of **1a**:**1b** was calculated using the integral values of their NH, H-2, H-3, H-4α, H-4β, H-6 and H-10 signals in the ^1^H-NMR spectrum measured after over a week of time period in CDCl_3_. The ^1^H signals in this Table except for the signals of 2-OH and 8-OH were from the ^1^H-NMR spectrum measured after over a week in CDCl_3_, where the 2-OH and 8-OH proton signals had already disappeared; ^b^ Chemical shift values (δ_H_ and δ_C_) were recorded using the internal TMS signals (δ_H_ and δ_C_ both 0.00) as references, respectively; ^c^ Multiplicities of the carbon signals were determined by DEPT experiments and are shown as s (singlet), d (doublet), t (triplet) and q (quartet), respectively; ^d^ Numbers in each line of this column indicate the protons that correlated with the proton in the corresponding line in ^1^H–^1^H COSY; ^e^ Numbers in each line of this column indicate the protons that showed NOE correlations with the proton in the corresponding line in NOESY or 1D difference NOE experiments. The NOEs between two protons in a spin coupling relationship were detected by the 1D difference NOE experiments; ^f^ Numbers in each line of this column indicate the carbons that showed HMBC correlations with the proton in the corresponding line in the HMBC experiments optimized for the 8.3 Hz of long-range *J*_CH_ value; ^g^ This OH signal was from the ^1^H-NMR spectrum measured soon after dissolving samples in CDCl_3_, which disappeared after over a week in the CDCl_3_ solution.

Compound **1** is a new compound possessing a cyclohexane bisoxirane moiety as a part of the 1-oxaspiro[4,5]decane ring system. Only four compounds in this class have so far been reported from natural sources [42,43,44,45], including taranorosinol B [44] with a C-C substituent at C-8. Thus **1** provides a new member of this rare class of natural products that also has a C-C substituent at C-8 and was obtained by activated production of silent metabolites in a fungus.

Penicimutanin A (**2**) and B (**3**) were obtained both as crystalline solids (MeOH), mp 131–132 °C, 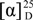 −99.7 (*c* 0.5, MeOH) for **2** and mp 125–126 °C, 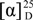 −65.8 (*c* 0.2, MeOH) for **3**. The molecular formulae, C_51_H_66_N_4_O_8_ for **2** and C_51_H_66_N_4_O_9_ for **3**, were assigned by HRESIMS. The UV absorptions of **2** [λ_max_ nm (log *ɛ*) in MeOH: 298 (3.47), 244 (4.10) and 209 (4.69)] and **3** [λ_max_ nm (log ɛ) in MeOH: 297 (3.42), 243sh and 209 (4.64)] revealed the presence of an indoline chromophore [41,46,47,48,49,50]. Their IR spectra closely resembled each other and showed absorption bans due to OH/NH, CH_3_/CH_2_, and CO/NHCO groups (IR spectra in the SI). The NMR spectra of **2** in CDCl_3_ showed two sets of ^1^H and ^13^C signals (Table 5): One set was similar to those of **1** and the other set resembled signals from **5**, although it lacked COCH_3_ signals [41]. Analyses of the ^1^H and ^13^C-NMR spectra with the DEPT, GOESY, ^1^H-^1^H COSY, HMQC, and HMBC techniques (Table S3 in the SI) established two structural parts in **2**: The down-half part corresponding to **1** and the up-half part corresponding to the skeletal unit of **5** (Figure 1). The connection of the two structural parts between N-6 and C-2′ was established by the HMBCs on H-5a/C-2′, H-2′/C-5a, and H-2′/C-6a. Thus, the planar structure of **2** was deduced. The ^1^H and ^13^C-NMR spectra of **3** in CDCl_3_ closely resembled those of **2** except an additional hydroxyl proton 16-OH signal was detected. There were also changes in several ^1^H and ^13^C signals ascribed to the benzene ring numbered 13–18 (Table 5). These data revealed that **3** is a hydroxylated derivative of **2**, and this was confirmed by analyses of the DEPT, GOESY, ^1^H–^1^H COSY, HMQC, and HMBC spectra (Table S4 in the SI). The 16-OH group in **3** was eventually attached to C-16 by the NOEs on 16-OH/H-15 and 16-OH/H-17 to complete its planar structure.

In GOESY, NOESY, and ROESY experiments, both **2** and **3** gave NOEs on H-3/H-11a, H-11a/Hα-11, Hα-11/H-10, Hβ-11/H-20, Hβ-11/H_3_-19a, Hβ-11/H_3_-19b, H_3_-19a/H-5a, and H_3_-19b/H-5a. These analyses established the relative stereochemistries of the indoline diketopiperazine rings in **2** and **3** as shown in Figure 1. The long-range couplings of H-3 and H-11a (1.4 Hz for **2**, 1.3 Hz for **3**) supported the *cis* relationship between these protons [46].

**Table 5:** 600 MHz ^1^H and 150 MHz ^13^C-NMR data of **2** and **3** in CDCl_3_^a^.

No.	2		3	
	δ_C_	δ_H_ (*J* in Hz)	δ_C_	δ_H_ (*J* in Hz)
1	167.9 s	—	168.2 s	—
2 (NH)	—	5.702 s	—	5.99 br s
3	56.1 d	4.30 ddd (9.3, 3.3, 1.4)	56.2 d	4.22 ddd (8.7, 3.4, 1.3)
4	164.1 s	—	164.1 s	—
5a	79.8 d	5.96 br s	79.4 d	5.96 br s
6a	148.1 s	—	148.2 s	—
7	108.8 d	6.71 d (7.5)	108.6 d	6.70 d (7.8)
8	128.8 d	7.10 td (7.5, 0.8)	128.8 d	7.10 td (7.8, 0.9)
9	119.8 d	6.78 t (7.5)	119.8 d	6.77 t (7.8)
10	125.3 d	7.13 br d (7.5)	125.3 d	7.13 br d (7.8)
10a	130.0 s	—	130.0 s	—
10b	60.7 s	—	60.7 s	—
11	38.4 t	Hα 2.45 dd (12.2, 5.5) Hβ 2.14 dd (12.2, 11.6)	38.5 t	Hα 2.42 dd (12.3, 5.5) Hβ 2.089 dd (12.3, 11.5)
11a	58.6 d	3.95 ddd (11.6, 5.5, 1.4)	58.3 d	3.92 ddd (11.5, 5.5, 1.3)
12	37.5 t	Ha 3.37 dd (14.4, 3.3) Hb 2.90 dd (14.4, 9.3)	36.7 t	Ha 3.20 dd (14.4, 3.4) Hb 2.86 dd (14.4, 8.7)
13	135.4 s	—	126.6 s	—
14	129.2 d	7.17 br d (7.2)	130.5 d	6.99 d (8.6)
15	129.3 d	7.31 br t (7.2)	116.1 d	6.76 d (8.6)
16	127.6 d	7.26 br t (7.2)	155.5 d	—
16-OH	—	—	—	6.55 br s
17	129.3 d	7.31 br t (7.2)	116.1 d	6.76 d (8.6)
18	129.2 d	7.17 br d (7.2)	130.5 d	6.99 d (8.6)
19	41.1 s	—	41.1 s	—
19a	22.2 q	1.03 s	22.2 q	1.02 s
19b	23.0 q	0.96 s	23.0 q	0.94 s
20	143.4 d	5.90 dd (17.3, 10.9)	143.4 d	5.88 dd (17.4, 10.7)
21	114.6 t	H *cis* 5.11 d (10.9)H*trans* 5.06 d (17.3)	114.6 t	H *cis* 5.10 d (10.7)H*trans* 5.04 d (17.4)
2′	89.9 d	5.69 d (9.5)	89.8 d	5.67 d (9.0)
3′	49.7 d	5.55 br s	49.5 d	5.59 br s
3′-NH	—	6.13 br s	—	6.21 br s
4′	38.2 t	Hα 2.12 dd (13.5, 9.6)Hβ 2.76 dd (13.5, 8.8)	38.1 t	Hα 2.096 dd (13.3, 9.7)Hβ 2.69 dd (13.3, 8.5)
5′	74.7 s	—	74.6 s	—
6′	57.4 d	3.40 dd (3.3, 2.7)	57.4 d	3.38 dd (3.3, 2.7)
7′	58.3 d	3.29 dd (3.4, 3.3)	58.3 d	3.28 dd (3.4, 3.3)
8′	66.4 s	—	66.4 s	—
8′-OH	—	4.63 s	—	4.66 br s
9′	57.9 d	3.23 dd (3.4, 3.2)	57.9 d	3.22 dd (3.4, 3.2)
10′	57.6 d	3.50 dd (3.2, 2.7)	57.6 d	3.47 dd (3.2, 2.7)
11′	47.1 t	2H 3.07 s	47.1 t	2H 3.06 s
12′	211.2 s	—	211.1 s	—
13′	31.7 q	3H 2.27 s	31.7 q	3H 2.26 s
1"	166.2 s	—	166.4 s	—
2"	121.2 d	5.70 br d (15.3)	121.1 d	5.70 br d (15.2)
3"	151.4 d	6.73 dd (15.3, 8.4)	151.6 d	6.73 dd (15.2, 8.2)
4"	34.1 d	2.40‒2.31 m	34.1 d	2.40‒2.31 m
5"	44.0 t	Ha 1.34‒1.30 mHb 1.12‒1.06 m	44.0 t	Ha 1.34‒1.29 mHb 1.12‒1.06 m
6"	30.3 d	2H 1.42‒1.34 m	30.4 d	2H 1.41‒1.32 m
7"	37.4 t	Ha 1.26‒1.15 mHb 1.08‒1.03 m	37.4 t	Ha 1.26‒1.15 mHb 1.08‒1.03 m
8"	26.8 t	2H 1.26‒1.15 m	26.8 t	2H 1.26‒1.15 m
9"	29.7 t	2H 1.26‒1.15 m	29.7 t	2H 1.26‒1.15 m
10"	31.9 t	2H 1.26‒1.15 m	31.9 t	2H 1.26‒1.15 m
11"	22.6 t	2H 1.30‒1.24 m	22.6 t	2H 1.30‒1.24 m
12"	14.1 q	3H 0.87 t (7.1)	14.1 q	3H 0.87 t (7.0)
13"	20.4 q	3H 0.99 d (6.7)	20.4 q	3H 0.98 d (6.7)
14"	19.4 q	3H 0.81 d (6.5)	19.4 q	3H 0.81 d (6.4)

^a^ The *δ*_C_ and *δ*_H_ values were recorded using internal TMS signals (*δ*_C_ and *δ*_H_: 0.00) as references, respectively.

**Figure 3:**
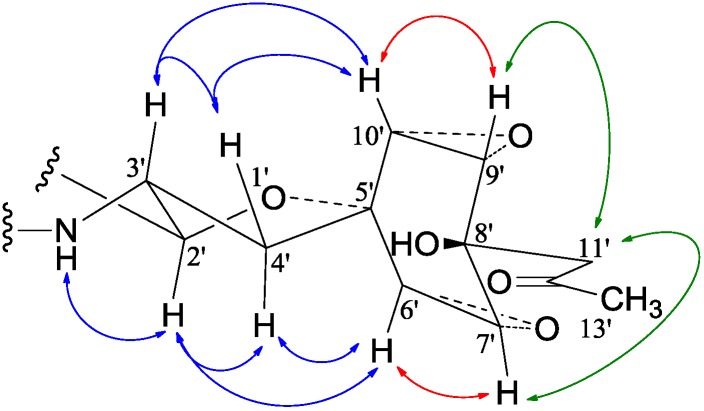
Relative stereochemistry of the spiro ring system in **2** and **3**. Key NOE interactions are indicated by arrows.

The relative stereochemistries of the spiro ring systems in **2** and **3** were established by the NOE interactions (Figure 3) and the couplings of related protons. The NOEs of H-3′/H-10′ and H-2′/H-6′ established the 2′,3′-*trans* relationship. The couplings of H-2′ and H-3′ (9.5 Hz for **2**, 9.0 Hz for **3**) indicated a dihedral angle greater than 160° between H-2′ and H-3′ [42,43]. These data defined the conformation of the five-membered ring as shown in Figure 3. The boat conformation of the cyclohexane ring was inferred from the four-bond couplings of H-6′/H-10′ and H-7′/H-9′ (Table 5 and Tables S3 and S4 in the SI) [42,43], as seen in **1**. The 8′-CH_2_COCH_3_ and 8′-OH in **2** and **3** were disposed similarly to the 8-CH_2_COCH_3_ and 8-OH in **1**. The well-matched NMR data of these groups in **1**–**3** (Table 4 and Table 5) indicated the same spatial orientations of these groups. The co-generation of **1**–**3** in the BD-1-3 mutant further provided additional support for a similar biogenetic origin. The large couplings of H-2′′/H-3′′ (15.3 Hz for **2**, 15.2 Hz for **3**) and the NOEs on H-2′′/H-4′′ and H-2′′/H_3_-13′′ defined the *E* configuration of the 2′′,3′′-double bond. The absolute configuration at C-3, 5a, 10b, and 11a in **2** and **3** was tentatively assigned as 3*S*,5a*S*,10b*S*,11a*S*, the same as in **5** [41], in view of a supposed common biogenetic origin of **2**, **3** and **5** in the BD-1-3 mutant.

Although some diketopiperazine dimers have been reported [46,47,48,49,50], their structures were composed of similar diketopiperazine monomers and differed from the structures of **2** and **3**. To our knowledge, **2** and **3** are novel structures with dimerized indoline diketopiperazine and 1-oxaspiro[4,5]decane ring moieties. It seems likely that the condensation of **1** and **4** between C-2 in **1** and N-1 in **4**, along with inverted prenyl transference from N-1 to C-3 coupled with cyclization between C-2 and N-12 in **4** by enzymatic processes should produce **2**, which underwent further oxidation at C-16 to give **3**. The condensation of **1** and **5** between C-2 in **1** and N-1 in **5** may be another possible way to produce **2** and **3**. Although the condensation of **1** and **5** without enzymatic process may probably produce **2** as an artificial product in the separation procedure, it seems unlikely that **2** further underwent selective oxidation at C-16 to give **3** in the extraction conditions. Furthermore, **1**–**5** were all detected in the mutant BD-1-3 extract as described in Section 2.6. Therefore, the structures of **2** and **3** may originate from mixed biosynthetic pathways involving the formation of the indoline diketopiperazine skeleton seen in **5** and the spiro ring moiety in **1**. Because **2** and **3** were both generated by the BD-1-3 mutant together with **1**, **4** and **5**, it is possible that more than one pathway was activated by the DES mutagenesis.

Penicimutatin (**4**), a white crystalline powder (MeOH), mp 275–276 °C, 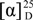 −112.0 (*c* 0.025, MeOH), was assigned the molecular formula C_25_H_27_N_3_O_2_ by HRESIMS. The UV spectrum gave an absorption curve the same as that of *cyclo*-(l-Trp-l-Phe) [47]. The IR spectrum gave absorptions due to NHCO and CH_3_/CH_2_ groups. The ^1^H and ^13^C-NMR spectra of **4** in DMSO-*d*_6_ resembled those of *cyclo*-(l-Trp-l-Phe) except for the appearance of additional signals from an isoprenyl group instead of the NH proton H-1 signal [51]. Analyses of the ^1^H–^1^H COSY, HMQC, and HMBC spectra (Table 6) confirmed the structural parts of the *cyclo*-(Trp-Phe) unit and the isoprenyl group in **4**. The isoprenyl group was linked to N-1 by the HMBCs on H_2_-1′/C-2 and H_2_-1′/C-9. Then, the stereochemistry of **4**, including absolute configurations, was established as follows. The NOEs on H-11/H-14 in the NOESY spectrum indicated the spatial proximity of the two protons. This indicated that the diketopiperazine ring was in a boat conformation with the boat-head carbons C-11/C-14 carrying *pseudo*-axial H-11/H-14. In this boat conformation, the dihedral angles between H-11 and H-16 and H-13 and H-14 were 110–120°, which matched the couplings of H-11/H-16 (^3^*J*_HH_ = 2.5 Hz) and H-13/H-14 (^3^*J*_HH_ = 2.4 Hz) [52]. The absolute configuration of **4** was determined by CD data. The CD spectrum of **4** in MeOH showed positive Cotton effects at 230–240 nm and negative Cotton effects at 240–300 nm. The signs of the Cotton effects coincided well with those reported for *cyclo*-(l-Trp-l-Phe) [51]. Thus, the absolute configuration at C-11 and C-14 in **4** was defined as *S*,*S*, the same as *cyclo*-(l-Trp-l-Phe) [51].

The structure of **4** was simple, but unique, in carrying a regular prenyl group at N-1. A similar *cyclo*-(l-Trp-l-Phe) with a regular or reverse prenyl group at C-2 has been reported [53,54]. A few of the tryptophan-derived simple cyclic dipeptides were known to carry regular prenyl groups at N-1 [55]. They were all synthesized by chemo-enzymatic [53,54] methods or chemical reactions [55], and have not been isolated from natural sources. Therefore, it is of interest that **4** was produced in the BD-1-3 mutant, which was acquired by mutagenesis of the fungal G59 strain.

**Table 6:** 400 MHz ^1^H and 100 MHz ^13^C-NMR data of **4** in DMSO-*d*_6_^a^.

Position	δ_C_^b,c^	δ_H_ (*J* in Hz) ^b^	COSY ^d^	NOE ^e^	HMBC ^f^
2	127.5 d	6.98 s		H_2_-10, H-11,16, 5′	C-3, 8, 9
3	108.3 s	—	—	—	—
4	119.2 d	7.49 br d (7.8)	H-5	H_2_-10, H-11, 16	C-3, 6, 8, 9
5	118.6 d	7.01 ddd (7.8, 7.0, 0.9)	H-4, H-6		C-7, 8
6	121.0 d	7.12 ddd (8.2, 7.0, 1.0)	H-5, H-7		C-4, 9
7	109.8 d	7.34 br d (8.2)	H-6	H-5′	C-5, 8
8	128.1 s	—	—	—	—
9	135.7 s	—	—	—	—
10	29.6 t	Ha 2.78 dd (14.6, 4.3) Hb 2.51 dd (14.6, 5.6)	Hb-10, H-11 Ha-10, H-11	H-2, 4, 16 H-2, 4	C-2, 3, 8, 11 C-2, 3, 8, 11
11	55.3 d	3.96 m	H_2_-10, H-16	H-2, 4, 14, 16, H_2_-10	
12	166.8 s	—	—	—	—
13 (NH)	—	7.74 d (2.5)	H-14	H-19, 23	C-11, 15
14	55.6 d	3.82 m	H_2_-17, H-13	H-11	
15	166.2 s	—	—	—	—
16 (NH)	—	7.97 d (2.4)	H-11	H-2, 4, Ha-10	C-12, 14
17	40.0 t	Ha 2.44 dd (13.5, 4.5) Hb 1.83 dd (13.5, 7.0)	H-14, Hb-17 H-14, Ha-17	H-19,23 H-19,23	C-14, 18, 19, 23 C-14, 18, 19, 23
18	136.6 s	—	—	—	—
19	129.7 d	6.66 dd (7.4, 2.0)	H-20, H-21	H-14, H_2_-17	C-21, 23
20	128.0 d	7.19–7.13 m			C-18, 22
21	126.4 d	7.19–7.13 m			C-19, 23
22	128.0 d	7.19–7.13 m			C-18, 20
23	129.7 d	6.66 dd (7.4, 2.0)	H-21, H-22	H-14, H_2_-17	C-19, 21
1′	43.4 t	2H 4.69 d (6.9)	H-2′, H-4′,5′		C-2, 9, 2′, 3′
2′	120.6 d	5.25 br t (6.9)	H_2_-1′, H-4′,5′	H-4′	
3′	135.1 s	—	—	—	—
4′	25.3 q	1.63 s	H_2_-1′, H-2′	H-2′, H-5′	C-2′, 3′, 5′
5′	17.8 q	1.78 s	H_2_-1′, H-2′	H-2,7, 4′	C-2′, 3′, 4′

^a^ Signal assignments were based on the results of ^1^H–^1^H COSY, HMQC, HMBC, NOESY, and 1D difference NOE experiments; ^b^ Chemical shift values (δ_H_ and δ_C_) were recorded using the DMSO-*d*_6_ signals (δ_H_ 2.50 and δ_C_ 39.50) as references, respectively; ^c^ Multiplicities of the carbon signals determined by HMQC are shown as s (singlet), d (doublet), t (triplet) and q (quartet), respectively; ^d^ Numbers in each line of this column indicate the protons that correlated with the proton in the corresponding line in ^1^H–^1^H COSY; ^e^ Numbers in each line of this column indicate the protons that showed NOE correlations with the proton in the corresponding line in NOESY and difference NOE experiments; ^f^ Numbers in each line of this column indicate the carbons that showed HMBC correlations with the proton in the corresponding line in the HMBC experiments optimized for the 8.3 Hz of long-range *J*_CH_ value.

### 2.8. Inhibitory Effect of 1–5 on Several Human Cancer Cell Lines

The antitumor activities of **1**–**5** were tested using MTT assay and human cancer cell lines K562, HL-60, HeLa, BGC-823, and MCF-7. Compounds **1**–**3** inhibited these cell lines with half-inhibitory concentration (IC_50_) values lower than 20 µM (Table 7); **4** and **5** also showed inhibitory effects on several cell lines by IR% values at 100 µg/mL: 22.6% (K562), 17.9% (HeLa) and 26.5% (MCF-7) for **4**, and 20.8% (K562), 55.3% (HeLa), 65.6% (MCF-7), and 34.8% (BGC-823) for **5**. The positive control 5-fluorouracil inhibited these cell lines with the IR% values of 48.5% (K562), 38.2% (HL-60), 37.4% (HeLa), 47.8% (BGC-823), and 47.4% (MCF-7) at 100 µg/mL.

**Table 7:** IC_50_ (µM) values of **1**–**3** on human cancer cell lines.

Compound	K562	HL-60	HeLa	BGC-823	MCF-7
**1**	17.4	4.2	10.9	12.6	8.6
**2**	11.4	5.4	9.5	8.0	5.4
**3**	19.9	12.1	17.7	16.6	8.0

In summary, we have demonstrated a practical strategy to activate silent secondary metabolite production in fungi using a marine-derived fungal G59 strain and a modified method of DES mutagenesis. By this strategy, four new antitumor compounds have been discovered by activating silent secondary metabolites in the fungal G59 strain, including **1**, **2** and **3** with novel structures. These discoveries provide rationale for increased use of chemical mutagenesis strategies in silent fungal metabolite studies.

It is noteworthy that there are two major advantages of the mutation-based strategy presented here. First, the mutants with activated pathways are obtained by single colony isolation without any complex gene manipulations. Therefore, this strategy is suited to microbial chemists for their simple application. Second, metabolites produced by the activated pathways are obtained by general fermentation without supplementation of additional agents into the culture medium. This differs from chemical epigenetic methodologies, which involve adding small molecule epigenetic modifiers to cultures to activate silent pathways [14,15,16] and restrict utilization for fungal isolates that are not resistant to the small molecule epigenetic modifiers [15]. On the other hand, the same medium compositions and fermentation conditions that have been used for the original strains were used in the mutant fermentation for the activated secondary metabolite production in the present strategy. This differs from the OSMAC strategies [13], which require changes of the medium compositions and/or fermentation conditions for activating silent pathways.

Finally, the strategy presented here could be easily extended to more mutagens and could be combined with other bioassays to improve more efficiently the diversity of bioactive metabolites of fungal origin.

## 3. Experimental Section

### 3.1. General Experimental

Melting points were measured on a Beijing Tiandiyu X-4 exact micro melting point apparatus and the temperatures were not corrected. Optical rotations were measured on an Optical Activity Limited polAAr 3005 spectropolarimeter or a Rudolph Research Autopol II spectropolarimeter. ESIMS was recorded on an Applied Biosystems API 3000 LC-MS spectrometer. HRESIMS was measured on an Agilent 6520 Q-TOF LC-MS spectrometer or Agilent 6520 Q-TOP mass spectrometer. UV data were recorded on a GBC Cintra 20 spectrophotometer. IR spectra were taken on a Bruker hyperion ATR-objective spectrophotometer. CD data were taken on a JASCO J-815 spectropolarimeter or a Biologic Science MOS 450 CD spectropolarimeter. All 1D and 2D NMR spectra were obtained on a JEOL JNM-GX 400 (400 MHz ^1^H and 100 MHz ^13^C-NMR) or Bruker-600 (600 MHz ^1^H and 150 MHz ^13^C-NMR) NMR spectrometer.

Precoated silica gel GF_254_ plates (10 cm × 20 cm, 0.25 mm thickness; Yantai Chemical Industrial Institute, Yantai, China) were used in TLC and spots were detected under UV light (254 and 365 nm) or by using Vaughan’s reagent [20] or 10% sulfuric acid reagent. Silica gel H (100–200 mesh, Yantai Chemical Industrial Institute, Yantai, China) and Sephadex^™^ LH-20 (GE Healthcare, Uppsala, Sweden) were used for column chromatography. HPLC was performed on Waters HPLC systems equipped with Waters 600 controller, Waters 600 pump, Waters 2414 refractive index detector, Waters 2996 (for analytical HPLC) or 2998 (for preparative HPLC) photodiode array detector and Waters Empower™ software (Waters, Milford, USA). Venusil MP C18 (5 μm, 100 Å, 4.6 mm × 250 mm; Agela Technologies, Tianjin, China), Capcell Pak C18 (UG80Å, 4.6 mm × 250 mm; Shiseido Co., Ltd., Tokyo, Japan), and Capcell Pak C18 (UG120Å, 4.6 mm × 250 mm; Shiseido Co., Ltd., Tokyo, Japan) columns were used in analytical HPLC, and Capcell Pak C18 (UG80Å, 10 mm × 250 mm; Shiseido Co., Ltd., Tokyo, Japan) and Capcell Pak C18 (UG120Å, 20 mm × 250 mm; Shiseido Co., Ltd., Tokyo, Japan) columns were used in semi-preparative or preparative HPLC.

ZHWY-2102 rotary shakers (Shanghai ZhiCheng Analyzing Instrument Manufactory Co., Ltd., Shanghai, China) were used for fermentation. The VERSAmax-BN03152 micro plate reader (Molecular Devices, San Francisco, USA) was used to read the optical density (OD), and the AE31 EF-INV inverted microscope (Motic China Group Co., Ltd., Xiamen, China) was used for morphological examination of tumor cells.

Human chronic myelogenous leukemia K562 cell line was provided by Lili Wang (Beijing Institute of Pharmacology and Toxicology, Beijing, China). Human acute promyelocytic leukemia HL-60, human cervical cancer HeLa, Human gastric adenocarcinoma BGC-823, and human breast cancer MCF-7 cell lines were provide by Wenxia Zhou (Beijing Institute of Pharmacology and Toxicology). Fetal bovine serum was purchased from Tianjin Hao Yang Biological Manufacture Co., Ltd. (Tianjin, China). The RPMI-1640 medium was purchased from Gibco (lot No. 1403238, Grant Island, USA) and MTT from Amresco (lot No. 0793, Solon, USA). Streptomycin (lot No. 071104) and penicillin (lot No. X1103302) were both purchased from North China Pharmaceutical Group Corporation, Beijing, China. The 5-fluorouracil (5-FU, lot No. 5402) was purchased from Aladdin Chemistry Co., Ltd. (Shanghai, China).

### 3.2. MTT Assay

EtOAc extracts and fractions were dissolved in MeOH at 10 mg/mL and the MeOH solutions were used in MTT assays. Pure compounds and 5-FU were dissolved in MeOH and DMSO to prepare 10.0 mg/mL stock solutions, respectively, and serial dilutions were made for MTT assay. The 5-FU was used as positive control, and MeOH and DMSO were used as blank controls, respectively.

The MTT assay was performed according to our previous procedure [24], and exponentially growing K562, HL-60, HeLa, BGC-823 and MCF-7 cells were treated with samples at 37 °C for 24 h. The assay was run in triplicate, and the OD value was read at 570 nm. The IR% was calculated using OD mean values by the formula, IR% = (OD_control_ − OD_sample_)/OD_control_ × 100%, and the IC_50_ value for a sample was obtained from IR% values of the samples at different concentrations.

### 3.3. Activating Silent Metabolites in Penicillium purpurogenum G59 by DES Mutagenesis

#### 3.3.1. Initial Strain and Preparation of the Spore Suspension

*Penicillium purpurogenum* G59, a marine-derived wild-type fungal strain used as the initial strain in the present study, was initially isolated by our group [38], and has been deposited at the China General Microbiological Culture Collection Center under the accession number CGMCC No.3560. The spore suspension was prepared using fresh spores by the method that we previously reported [20].

#### 3.3.2. DES Mutagenesis of Strain G59 and Mutant Selection

To each of four sterilized 2 mL Eppendorf tubes was added 1600 μL of the G59 spore suspension, respectively, and 400 μL fresh DMSO was added to each tube. Then, 0, 10, 20, 40 μL of DES was added to the four tubes, respectively, to obtain a series of spore suspensions in 20% (v/v) DMSO with 0%, 0.5%, 1% and 2% (v/v) of DES. The one without DES was used as a control, and the other three were used as the test groups. Similarly, to each of four sterilized 2 mL Eppendorf tubes was added 1000 µL of the G59 spore suspension, respectively, and 1000 µL fresh DMSO was added to each tube. Then, 0, 10, 20, 40 μL of DES was added to the tubes, respectively, to obtain a series of spore suspensions in 50% (v/v) DMSO with 0%, 0.5%, 1% and 2% (v/v) of DES. The one without DES was used as a control, and the other three were used as the test groups. The G59 spores in the 20% (v/v) and 50% (v/v) DMSO tubes were treated at 4 °C for 1–30 days. During the treatment period, each 80 μL portion of the treated spore suspensions was sampled and spread on PDA plates at 1, 2, 7, 10, 15, and 30 days of treatment followed by incubation at 28 °C for 5–7 days. Mutants from the test groups in 20% (v/v) and 50% (v/v) DMSO were obtained by selection of colonies with different appearances.

#### 3.3.3. Fermentation and Preparation of EtOAc Extract for MTT Assay and Chemical Analysis

For the first round MTT test, the initial G59 strain and all of the 42 mutants were inoculated onto PDA plates from their PDA tube slants stocked at 4 °C and activated by incubation at 28 °C for 3–5 days. The activated, fresh G59 strain and 42 mutants were inoculated into a Erlenmeyer (500 mL) containing 200 mL of liquid medium (glucose 2%, maltose 1%, mannitol 2%, glutamic acid 1%, peptone 0.5%, and yeast extract 0.3% in distilled water) and fermented at 28 °C for 12 days on a rotary shaker at 200 rpm. To each 200 mL of the fermentation broth was added 400 mL acetone and then extracted by ultra-sonication for 2 h. Then, the aqueous acetone solution obtained by filtration was concentrated under reduced pressure to remove acetone, and the remaining water suspension was extracted three times with equal volumes of EtOAc (3 × 200 mL). The EtOAc solutions were combined, and the EtOAc removed *in vacuo*, followed by freeze-drying. These afforded the EtOAc extracts for the first round MTT assay.

An additional two rounds of individual fermentations and extractions were carried out for the control G59 strain and the 31 mutants that showed antitumor activities in the EtOAc extracts from the first round MTT test. The fermentations and extractions were performed with the same conditions as the first round, for each of the control G59 and the 31 mutant strains. The EtOAc extracts from the fermentations were subjected to MTT tests to confirm the activities. The EtOAc extracts from the third round of fermentation were used for HPLC-PDAD-UV and HPLC-ESI-MS analyses.

#### 3.3.4. HPLC-PDAD-UV Analysis of EtOAc Extracts of the G59 Strain and 31 Mutants

The HPLC-PDAD-UV analysis was performed using an analytical Venusil MP C18 column (5 µm, 100 Å, 4.6 mm × 250 mm; Agela Technologies) on the same HPLC system previously described. Active extracts were dissolved in MeOH (10.0 mg/mL) and injected (10 µL) into the column and eluted with a MeOH-H_2_O linear gradient (20%→100% MeOH in 60 min followed by 30 min with isocratic 100% MeOH) mobile phase (1 mL/min flow rate). The acquired photodiode array data (PDAD) were processed by Empower PDA software (Waters, Milford, USA).

#### 3.3.5. HPLC-ESI-MS Analysis of EtOAc Extracts of the G59 Strain and four Mutants

The EtOAc extracts of the control G59 strain and four bioactive mutants, AD-1-2, AD-2-1, BD-1-3 and BD-1-6, were subjected to HPLC-ESI-MS analysis. The HPLC-ESI-MS analysis was performed on an LC-MS equipment equipped with an Agilent 1100 HPLC system, AB Sciex API 3000 LC-MS/MS system, and AB Sciex Analyst 1.4 software (AB SCIEX, Framingham, USA). The EtOAc extracts of the G59 strain and the four mutants dissolved in MeOH (10 mg/mL) were used for the HPLC-ESI-MS analysis. HPLC was carried out on a Venusil MP C18 column (5 µm, 100 Å, 4.6 mm × 250 mm; Agela Technologies, Tianjin, China) using the same MeOH-H_2_O linear gradient. The mass detector was set to scan a range from *m*/*z* 150–1500 in both positive and negative modes. The acquired data were processed by Analyst 1.4 software.

### 3.4. Experiments for Investigation on Compounds 1–5 from Mutant BD-1-3

#### 3.4.1. Mutant Information

The mutant BD-1-3 was obtained by treatment of G59 spores in 20% (v/v) DMSO with 1% (v/v) DES at 4 °C for 1 day, its EtOAc extract inhibited the K562 cells with the IR% value of 58.1% (Table 3). This mutant was deposited at the China General Microbiological Culture Collection Center under the accession number CGMCC No. 4284.

#### 3.4.2. Large-Scale Fermentation and EtOAc Extract Preparation

The mutant BD-1-3 was inoculated onto PDA plates from a PDA slant stock (stored at 4 °C) and activated by incubation at 28 °C for 4 days. The activated mutant BD-1-3 was inoculated into a Erlenmeyer (500 mL) containing 200 mL of liquid medium (glucose 2%, maltose 1%, mannitol 2%, glutamic acid 1%, peptone 0.5% and yeast extract 0.3% in distilled water, adjusted to pH 6.0 prior to sterilization) and cultured at 28 °C for 48 h on a rotary shaker at 200 rpm. Each 10 mL of the culture broth was inoculated into fifteen Erlenmeyers (500 mL) containing 200 mL of the same liquid medium and further cultured under the same condition for 48 h to obtain a seed culture (3000 mL). Each 10 mL of the seed culture was inoculated into 200 Erlenmeyers (500 mL) with 200 mL of the same liquid medium. Then, these were cultured with shaking (200 rpm) at 28 °C for 12 days to obtain 40 L of fermentation broth.

The whole broth (40 L) was filtered to separate into the filtrate and the mycelial cake. The filtrate (36 L) was extracted three times with equal volumes of EtOAc (3 × 36 L). The mycelial cake was extracted three times with 80% (v/v) aqueous acetone (3 × 10 L) by ultra-sonication for 2 h followed by extraction at room temperature for 12 h in still. The aqueous acetone solution obtained by filtration was evaporated under reduced pressure to remove the acetone. The remaining water layer (5 L) was extracted three times with equal volumes of EtOAc (3 × 5 L). Both the extracts from the filtrate and mycelia showed the same spots on TLC examinations and thus were combined to afford a total of 40 g of EtOAc extract. The EtOAc extract inhibited the K562 cells (an IR% of 68.7% at 100 µg/mL), which was used for the isolation of **1**–**5**.

The parent G59 strain was fermented under the same conditions using three Erlenmeyers (500 mL) with 200 mL of the same liquid medium. Extraction of the whole broth (600 mL) as described above for mutant BD-1-3 provided an EtOAc extract (572 mg), which did not show an inhibitory effect on K562 cells (an IR% value of 6.8% at 100 µg/mL). This extract was used in the MTT assays, TLC analyses, in the HPLC-PDAD-UV and HPLC-ESI-MS analyses, as a negative control, during separation of **1**–**5**.

#### 3.4.3. Isolation of Compounds **1**–**5**

The EtOAc extract (40 g) of the mutant BD-1-3 was subjected to silica gel column (silica gel 180 g, bed 7.5 cm × 14 cm) chromatography by stepwise elution with b.p. 60–90 °C petroleum ether (P)-chloroform (C)-methanol (M) (PC 2:1→0:1 and then CM 99:1→0:1) to obtain 13 fractions: **Fr-1** (0.9 g, eluted by PC 2:1), **Fr-2** (1.3 g, eluted by PC 1:2), **Fr-3** (9.3 g, eluted by chloroform), **Fr-4** (2.1 g, eluted by chloroform), **Fr-5** (1.6 g, eluted by CM 99:1), **Fr-6** (2.1 g, eluted by CM 99:1), **Fr-7** (0.6 g, eluted by CM 98:2), **Fr-8** (6.2 g, eluted by CM 97:3), **Fr-9** (3.6 g, eluted by CM 95:5), **Fr-10** (2.7 g, eluted by CM 93:7), **Fr-11** (2.6 g, eluted by CM 9:1), **Fr-12** (3 g, eluted by CM 8:2), and **Fr-13** (eluted by methanol). Among them, ten fractions showed inhibitory effects on the K562 cells with the IR% values of 45.7% (**Fr-1**), 41.9% (**Fr-2**), 91.0% (**Fr-3**), 17.9% (**Fr-4**), 54.9% (**Fr-5**), 90.7% (**Fr-6**), 78.9% (**Fr-7**), 48.6% (**Fr-8**), 54.5% (**Fr-9**) and 46.1% (**Fr-10**) at 100 µg/mL. TLC analysis by direct comparison with the G59 control extract indicated that metabolites **1**–**5** were newly produced by the mutant and contained in three fractions: **1** in fraction **Fr-5**, **2**–**4** in fraction **Fr-6**, and **5** in fraction **Fr-4**. The other fractions, **Fr-1**–**Fr-3** and **Fr-7**–**Fr-10**, also contained newly produced antitumor metabolites and these metabolites are being investigated further.

**Fr-5** (1.6 g) was subjected to a Sephadex LH-20 column (bed 2 cm × 110 cm) in CHCl_3_-MeOH (1:1) and eluted with CHCl_3_-MeOH (1:1) to afford seven fractions: **Fr-5-1** (45 mg), **Fr-5-2** (90 mg), **Fr-5-3** (490 mg), **Fr-5-4** (860 mg), **Fr-5-5** (70 mg), **Fr-5-6** (20 mg) and **Fr-5-7** (20 mg). Among them, **Fr-5-3** inhibited the K562 cells with the IR% value of 81.5% at 100 μg/mL and was shown to contain **1** by TLC examination. **Fr-5-3** (490 mg) was subjected again to the same Sephadex LH-20 column (bed 2 cm × 110 cm, in CHCl_3_–MeOH 1:1). Elution of the column with CHCl_3_-MeOH (1:1) and careful collection of the eluent containing **1** gave a fraction (82 mg) with a large amount of this metabolite. This fraction (82 mg) was further subjected to silica gel (2.2 g) column chromatography in stepwise elution with chloroform-acetone to obtain crude **1** (25 mg, eluted by chloroform-acetone 5:1). The crude **1** (25 mg) was purified by semi-preparative HPLC (column: Capcell Pak C18, UG80Å, 10 mm × 250 mm, room temperature; mobile phase: MeOH-H_2_O 80:20; flow rate: 3.0 mL/min; detecting wave length: 210 nm) to give pure **1** (21 mg, *t*_R_ = 21.3 min).

**Fr-6** (2.1 g) was separated by Sephadex LH-20 column (bed 4.5 cm × 50 cm) chromatography eluted with EtOH to afford nine fractions in the order of elution: **Fr-6-1** (30 mg), **Fr-6-2** (45 mg), **Fr-6-3** (85 mg), **Fr-6-4** (300 mg), **Fr-6-5** (460 mg), **Fr-6-6** (236 mg), **Fr-6-7** (280 mg), **Fr-6-8** (210 mg) and **Fr-6-9** (440 mg). Among them, **Fr-6-4** (300 mg) was shown to contain **2** and **3** by TLC examination, which inhibited the K562 cells with the IR% of 51.9% at 100 µg/mL, while **Fr-6-8** (210 mg) contained **4** and inhibited the K562 cells with the IR% of 29.7% at 100 µg/mL. **Fr-6-4** (300 mg) was subjected again to a Sephadex LH-20 column (bed 1.5 cm × 80 cm, in CHCl_3_–MeOH 1:1). Elution of the column with CHCl_3_-MeOH (1:1) and collection of the eluent containing **2** and **3** gave a fraction (190 mg) containing the two metabolites as major components, which inhibited the K562 cells with the IR% of 54.2% at 100 µg/mL. This fraction (190 mg) was subjected to preparative HPLC (column: Capcell Pak C18, UG120Å, 20 mm × 250 mm, room temperature; mobile phase: MeOH-H_2_O 80:20; flow rate: 10.0 mL/min; detecting wave length: 210 nm) to give crude **2** (*t*_R_ = 29.9 min) and crude **3** (*t*_R_ = 66.2 min). Further purification of crude **2** and crude **3** by semi-preparative HPLC (column: Capcell Pak C18, UG80Å, 10 mm × 250 mm, room temperature; mobile phase: MeOH–H_2_O 86:14 for **2** and 82:18 for **3**; flow rate: 3.0 mL/min; detecting wave length: 210 nm) gave pure **2** (13 mg, *t*_R_ = 43.7 min) and **3** (5.5 mg, *t*_R_ = 37.0 min). **Fr-6-8** (210 mg) containing **4** was subjected to a Sephadex LH-20 column (bed 1.5 cm × 80 cm, in CHCl_3_-MeOH 1:1) and elution of the column with CHCl_3_-MeOH (1:1) afforded three fractions in the order of elution **Fr-6-8-1** (115 mg), **Fr-6-8-2** (50 mg) and **Fr-6-8-3** (30 mg). **Fr-6-8-2** (50 mg) was shown to contain **4** by TLC examination, which inhibited the K562 cells with an IR% of 25.4% at 100 µg/mL. Further separation of **Fr-6-8-2** (50 mg) by semi-preparative HPLC (column: Capcell Pak C18, UG80Å, 10 mm × 250 mm, room temperature; mobile phase: MeOH–H_2_O 75:25; flow rate: 3.0 mL/min; detecting wave length: 210 nm) afforded pure **4** (5.5 mg, *t*_R_ = 18.4 min).

**Fr-4** (2.1 g) was subjected to a silica gel column (26 g silica gel, bed 1.5 cm ×19 cm) and stepwise elution of the column with b.p. 60–90 °C petroleum ether (P)-EtOAc (E) gave four fractions: **Fr-4-1** (542 mg, eluted by PE 4:1), **Fr-4-2** (387 mg, eluted by PE 4:1), **Fr-4-3** (240 mg, eluted by PE 4:1) and **Fr-4-4** (853 mg, eluted by PE 1:1). Among them, **Fr-4-4** (853 mg) was shown to contain **5** by TLC examination. **Fr-4-4** (853 mg) was subjected to Sephadex LH-20 column (bed 2 cm × 110 cm) chromatography using CHCl_3_-MeOH (1:1) as eluting solvent. Careful collection of the eluent, tracing **5** by TLC, gave crude **5**. Further purification of the crude **5** by preparative HPLC (column: Capcell Pak C18, UG120Å, 20 mm × 250 mm, room temperature; mobile phase: MeOH-H_2_O 68:32; flow rate: 10.0 mL/min; detecting wave length: 210 nm) gave pure **5** (85 mg, *t*_R_ = 33 min).

#### 3.4.4. Physicochemical and Spectroscopic Data of **1**–**5**

Penicimutanolone (**1**): White crystalline powder (MeOH), m.p. 114–115 °C, 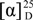 −11.9 (*c* 1.0, MeOH). Positive ESI-MS *m*/*z*: 480 [M + H]^+^, 502 [M + Na]^+^, 518 [M + K]^+^; negative ESI-MS *m*/*z*: 524 [M + HCO_2_]^−^. Positive HR-ESI-MS *m*/*z*: measured 480.2974 [M + H]^+^, calculated for C_26_H_42_NO_7_ [M + H]^+^ 480.2961; measured 502.2793 [M + Na]^+^, calculated for C_26_H_41_NO_7_Na [M + Na]^+^ 502.2781; measured 518.2530 [M + K]^+^, calculated for C_26_H_41_NO_7_K [M + K]^+^ 518.2520. UV λ_max_ nm (log ε) in MeOH: 215 (4.21). IR ν_max_ cm^−1^ (diamond ATR crystal): 3314, 2935, 2869, 1701, 1669, 1625, 1510, 1459, 1421, 1377, 1363, 1326, 1269, 1151, 1124, 1038, 986, 933, 859, 735. CD *∆*ε (nm) in MeOH: 0 (190.6), −1.12 (192.5), −1.07 (194.0), −1.37 (196.5), −1.82 (204.5), −2.21 (208.5), −2.13 (215), −2.07 (217.5), −1.89 (220.5), −1.04 (227.5), −0.25 (239.5), 0 (241.7), 0 (272.1), +0.18 (282.5), +0.15 (285.0), +0.18 (297), +0.16 (300.5), +0.15 (306.5), +0.29 (319.0), +0.29 (320.0), +0.26 (328.5), +0.27 (330.5), +0.34 (339.5), +0.37 (343.0), +0.44 (351.0), +0.29 (359.5), +0.27 (369.0), +0.24 (371.5), +0.22 (375.5), +0.32 (387.0), 0 (399.8). ^1^H and ^13^C-NMR data: in Table 4; see also Tables S1 and S2 in the SI.

Penicimutanin A (**2**): Yellowish crystalline solid, m.p. 131–132 °C, 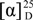 −99.7 (*c* 0.5, MeOH). Positive ESI-MS *m*/*z*: 863 [M + H]^+^, 885 [M + Na]^+^, 901 [M + K]^+^; negative ESI-MS *m*/*z*: 861 [M − H]^−^, 897 [M + Cl]^−^, 907 [M + HCO_2_]^−^. Positive HR-ESI-MS *m*/*z*: measured 863.4969 [M + H]^+^, calculated for C_51_H_67_N_4_O_8_ [M+H]^+^ 863.4959; measured 885.4780 [M + Na]^+^, calculated for C_51_H_66_N_4_O_8_Na [M + Na]^+^ 885.4778; measured 901.4518 [M + K]^+^, calculated for C_51_H_66_N_4_O_8_K [M + K]^+^ 901.4518. UV λ_max_ nm (log ε) in MeOH: 298 (3.47), 244 (4.10), 209(4.69). IR ν_max_ cm^−1^ (diamond ATR crystal): 3292, 3080, 2933, 2873, 1667, 1607, 1528, 1489, 1454, 1380, 1365, 1335, 1304, 1273, 1200, 1106, 1077, 1058, 1032, 983, 931, 895, 862, 815, 748, 703. CD *∆*ε (nm) in MeOH: −11.26 (195.5), 0 (205.2), +2.66 (208.5), 0 (210.2), −16.80 (215.5), 0 (225.6), +3.32 (232.0), 0 (237.0), −17.80 (249.5), 0 (315.1), +0.41 (319.0), +0.42 (320.0), +0.39 (321.5), +0.53 (328.5), +0.55 (330.0), +0.50 (331.5), +0.61 (340.0), +0.65 (344.0), +0.70 (351.0), +0.38 (358.0), +0.40 (359.0), +0.37 (361.5), +0.48 (367.5), +0.49 (369.0), +0.48 (371.5), +0.48 (374.5), +0.48 (375.5), +0.24 (381.5), +0.57 (387.5), 0 (390.9), +0.12 (394.0), +0.17 (398.0), 0 (399.9). ^1^H and ^13^C-NMR data: in Table 5; see also Tables S3 in the SI.

Penicimutanin B (**3**): Yellowish crystalline solid, m.p. 125–126 °C, 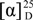 −65.8 (*c* 0.2, MeOH). Positive ESI-MS *m*/*z*: 879 [M + H]^+^; negative ESI-MS *m*/*z*: 877 [M − H]^−^, 913 [M + Cl]^−^. Positive HR-ESI-MS *m*/*z*: measured 879.4902 [M + H]^+^, calculated for C_51_H_67_N_4_O_9_ [M + H]^+^ 879.4908; measured 901.4824 [M + Na]^+^, calculated for C_51_H_66_N_4_O_9_Na [M + Na]^+^ 901.4728; measured 917.4656 [M+K]^+^, calculated for C_51_H_66_N_4_O_9_K [M + K]^+^ 917.4467. UV λ_max_ nm (log *ε*) in MeOH: 297 (3.42), 243sh, 209 (4.64). IR *ν*_max_ cm^−1^ (diamond ATR crystal): 3291, 2937, 2874, 1667, 1519, 1489, 1450, 1379, 1367, 1335, 1304, 1272, 1242, 1204, 1177, 1112, 1058, 1033, 984, 933, 896, 862, 751. CD *Δε* (nm) in MeOH: −16.04 (196.5), 0 (202.3), +6.05 (207.0), 0 (211.3), −10.85 (216.0), 0 (232.6), +0.87 (235.5), 0 (238.1), −11.04 (251.5), 0 (273.7), +0.55 (282.5), +0.24 (290.0), +0.33 (297.5), 0 (301.6), +0.60 (318.5), +0.46 (322.0), +0.49 (324.5), +0.71 (328.5), +0.72 (329.5), +0.73 (330.5), +0.74 (331.5), +0.80 (339.5), +0.79 (344.0), +0.76 (346.5), +0.84 (351.0), +0.64 (360.5), +0.65 (361.5), +0.55 (366.5), +0.60 (368.5), +0.42 (376.5), +0.41 (377.5), +0.51 (387.5), +0.19 (394.0), +0.14 (396.5), 0 (399.1). ^1^H and ^13^C-NMR data: in Table 5; see also Table S4 in the SI.

Penicimutatin (**4**): White crystalline powder (MeOH), m.p. 275–276 °C, 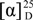 −112.0 (*c* 0.025, MeOH). Positive ESI-MS *m*/*z*: 402 [M + H]^+^, 424 [M + Na]^+^, 440 [M + K]^+^; negative ESI-MS *m*/*z*: 400 [M − H]^−^. Positive HR-ESI-MS *m*/*z*: measured 402.2208 [M + H]^+^, calculated for C_25_H_28_N_3_O_2_ [M + H]^+^ 402.2182; measured 424.2022 [M + Na]^+^, calculated for C_25_H_27_N_3_O_2_Na [M + Na]^+^ 424.2001; measured 440.1767 [M + K]^+^, calculated for C_25_H_27_N_3_O_2_K [M + K]^+^ 440.1740. UV (MeOH) λ_max_ (log ε): 289 (3.65), 221 (4.39), 207 (4.41). IR ν_max_ cm^−1^ (diamond ATR crystal): 3340, 3179, 3035, 2972, 2942, 2912, 2879, 1666, 1456, 1375, 1326, 1269, 1196, 1173, 1129, 848, 741, 702. CD *∆*ε (nm) in MeOH: −24.26 (214.5), 0 (230.6), +3.00 (235.0), 0 (250.1), −0.20 (253.5), −0.22 (264.5), −0.44 (271.5), −0.47 (280.0), −0.75 (288.0), −0.75 (295.0), 0 (306.8). ^1^H and ^13^C-NMR data: in Table 6.

Fructigenine A (**5**): White crystalline powder (MeOH), m.p. 94–95 °C, 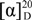 −129.3 (*c* 0.30, MeOH). Positive ESI-MS *m*/*z*: 444 [M + H]^+^, 466 [M + Na]^+^, 482 [M + K]^+^; negative ESI-MS *m*/*z*: 442 [M − H]^−^. CD *∆*ε (nm) in MeOH: −54.55 (216.5), 0 (234.0), +15.17 (251.0), 0 (270.6), −2.11 (283.5), 0 (301.8). ^1^H and ^13^C-NMR data: in Table 8. The ^1^H and ^13^C-NMR data of **5** are identical with those of fructigenine A in the literature [41], and the positive CD sign of **5** in the 245–255 nm region (*∆*ε_251.0_ +15.17) was consistent with that reported for 20,21-dihydrofructigenine A ([θ]_247_ +27400) [41].

**Table 8:** 400 MHz ^1^H and 100 MHz ^13^C-NMR data of **5** in CDCl_3_^a^.

Position	δ_C_	δ_H_ (*J* in Hz)	Position	δ_C_	δ_H_ (*J* in Hz)
1	164.8	—	11a	59.1	3.79 br dd (11.4, 5.6)
2 (NH)	—	5.77 s	12	36.1	3.54 dd (14.4, 2.5)/2.82 dd (14.4, 10.1)
3	55.9	4.24 dd (10.1, 2.5)	13	135.3	—
4	168.1	—	14	129.2	7.19 dd (6.7, 1.7)
5	—	—	15	129.3	7.37–7.24 m
5a	79.4	6.04 br s	16	127.7	7.37–7.24 m
6 (N)	—	—	17	129.3	7.37–7.24 m
6a	143.0		18	129.2	7.19 dd (6.7, 1.7)
7	124.5	8.01 br s	19	40.3	—
8	129.1	7.37–7.24 m	19a	23.6	1.13 3H, s
9	119.2	7.13 td (7.6, 0.8)	19b	23.2	0.97 3H, s
10	129.1	7.37–7.24 m	20	143.3	5.76 dd (17.3, 10.8)
10a	132.0	—	21	114.6	5.13 d (10.8)/5.11 d (17.3)
10b	60.9	—	22	170.1	—
11	36.9	2.56 dd (12.6, 5.6)/2.24 dd (12.6, 11.4)	23	22.4	2.66 3H, s

^a^ The δ_C_ and δ_H_ values were recorded using internal TMS signals (δ_C_ and δ_H_: 0.00) as references, respectively.

#### 3.4.5. HPLC-PDAD-UV/HPLC-ESI-MS Analyses of G59 and BD-1-3 Extracts for Detecting **1**–**5**

HPLC-PDAD-UV and HPLC-ESI-MS analyses of EtOAc extracts of the control G59 and the mutant BD-1-3 strains for detecting **1**–**5** were performed with the same conditions given as in sections 3.3.5 and 3.3.6 for HPLC-PDAD-UV and HPLC-ESI-MS, respectively. Compounds **1**–**5** in MeOH at 10 mg/mL were used as references both in the HPLC-PDAD-UV and HPLC-ESI-MS analyses.

In the HPLC-PDAD-UV analysis, compounds **1**–**5** were eluted as peaks with retention times (*t*_R_) of, 57.53 min for **1**, 65.05 min for **2**, 61.88 min for **3**, 52.65 min for **4**, and 50.03 min for **5**, and their examination were carried out using both retention times and UV absorption spectra. In the HPLC-ESI-MS analysis, both positive and negative ion peaks of **1**–**5** appeared at shorter retention times (*t*_R_: 54.14 min for **1**, 61.38 min for **2**, 58.30 min for **3**, 48.44 min for **4**, and 46.78 min for **5**) than those in their HPLC-PDAD-UV analysis because of the shortened flow length from the outlet of the HPLC column to the inlet of MS in HPLC-ESI-MS. Examination of **1**–**5** in the control G59 and the mutant BD-1-3 extracts were performed using selective ion ([M + Na]^+^ for **1**–**3**, [M − H]^−^ for **4** and **5**) monitoring with both chromatograms and related MS spectra.

## 4. Conclusions

The present study has presented a practical strategy for microbial chemists to simply access silent metabolites in fungi. Using this strategy, four new compounds **1**–**4** were discovered, together with a known fructigenine A (**5**), by activating silent metabolites in a marine-derived fungus *Penicillium purpurogenum* G59. Three new compounds **1**–**3** showed promising antitumor effects on several human cancer cell lines with IC_50_ values lower than 20 µM; **4** and **5** also inhibited some of the tested cell lines to different extents. Both bioassays and chemical investigations demonstrated the effectiveness of this strategy and the potential for discovering new compounds with antitumor activities from silenced fungal metabolic pathways.

## Supplementary Files

Supplementary File 1 Supplementary Information (PDF, 5588 KB)
