# Supplementary material for: A Practical Strategy to Discover New Antitumor Compounds by Activating Silent Metabolite Production in Fungi by Diethyl Sulphate Mutagenesis
Source: Mar Drugs. 2014 Mar 27;12(4):1788–814. doi: 10.3390/md12041788 (PMC4012455; doi:10.3390/md12041788)
Supplement: Supplementary File 1 — Supplementary Information (PDF, 5588 KB) [file marinedrugs-12-01788-s001.pdf]

Supplementary Information

Table of Contents

Tables

Table S1. A: 600 MHz ^{1}H and 150 MHz ^{13}C-NMR data of **1** in $CDCl_3$ (data from the spectra measured soon after dissolving **1** in $CDCl_3$, **1a**:**1b** = approx. 5.5:1)……………… S2

Table S1. B: 600 MHz ^{1}H and 150 MHz ^{13}C-NMR data of **1** in $CDCl_3$ (data from the spectra measured after over a week in $CDCl_3$, **1a**:**1b** = approx. 6.3:1)…………………… S4

Table S2. 400 MHz ^{1}H and 100 MHz ^{13}C-NMR data of **1** in CD_3OD (**1a**:**1b** = approx. 1.8:1)……………………………………………………………… S5

Datas for Calculating Ratios of **1a** and **1b** in $CDCl_3$ and CD_3OD Solutions………………… S7

Table S3. 600 MHz ^{1}H and 150 MHz ^{13}C-NMR data of **2** in $CDCl_3$………………………… S8

Table S4. 600 MHz ^{1}H and 150 MHz ^{13}C-NMR data of **3** in $CDCl_3$………………………… S10

Figures

Figure S1. Phenotypes of the parent strain G59 and its mutants growing on PDA plates by incubation at 28 ℃ for 5 days…………………………………………………… S12

Figure S2. HPLC-PDAD-UV analysis of the EtOAc extracts of the control G59 strain and selected mutants…………………………………………………………… S13

Figure S3. HPLC-ESI-MS analysis of the EtOAc extracts of the control G59 strain and selected mutants…………………………………………………………… S17

Figure S4. HPLC-PDAD-UV analysis of the EtOAc extracts of the strain G59 and the mutant BD-1-3 for detecting **1**–**5**………………………………………………… S21

Figure S5. HPLC-ESI-MS analysis of the EtOAc extracts of the strain G59 and the mutant BD-1-3 for detecting **1**–**5**………………………………………………… S22

Appendix of Spectra……………………………………………………………………… S25

Table S1. A: 600 MHz ^{1}H and 150 MHz ^{13}C-NMR data of **1** in $CDCl_3$ (data from the spectra measured soon after dissolving in $CDCl_3$, **1a**:**1b** = approx. 5.5:1) [a].

No.	**1a** (2,3-*cis*)					**1b** (2,3-*trans*)				
	**δ_C [b,c]**	**δ_H [b] (*J* in Hz)**	**COSY [d]**	**NOE [e]**	**HMBC [f]**	**δ_C [b,c]**	**δ_H [b] (*J* in Hz)**	**COSY [d]**	**NOE [e]**	**HMBC [f]**
2	96.45 s	5.56 br t (4.4)	H-3, NH, Hα-4	2-OH, H-3,10, NH, Hβ-4	C-3,4,5	102.61 s	5.58 br d (3.5)	H-3, Hα-4	2-OH, H-3,6, NH, Hα-4	C-3,4,5
2-OH	—	4.98 [g] br s		H-2		—	4.80 [g] br s		H-2	
3	52.11 d	4.69 dtd (10.6, 8.3, 4.4)	H-2, NH, H_2-4	H-2, NH, Hβ-4, H-10	C-2,4,1'	57.21 d	4.56 m	H-2, NH, H_2-4	H-2, NH, Hβ-4, H-10	C-2,5,1'
3-NH	—	6.17 [h] dd (8.3, 2.4)	H-2, H-3	H-2,3,6, Hα,β-4, H-2'	C-2,3,1',2'	—	6.32 br s	H-3	H-2,3,6, Hα,β-4, H-2'	
4	37.06 t	Hβ 2.58 dd (13.0, 8.3)	H-3, Hα-4	H-2,3, NH, Hα-4, H-10, HO-8	C-2,3,6,10	38.50 t	Hβ 2.63 br dd (13.0, 6.5)	H-3, Hα-4	H-3, NH, Hα-4, H-10, HO-8	C-2,3,5,6,10
		Hα 2.01 ddd (13.0, 10.6, 4.4)	H-2, H-3, Hβ-4	NH, Hβ-4, H-6, HO-8	C-3,5,6,10		Hα 2.18 br dd (13.0, 3.5)	H-2, H-3, Hβ-4	H-2, NH, Hβ-4, H-6, HO-8	C-2,3,6,10
5	79.05 s	—	—	—	—	80.45 s	—	—	—	—
6	59.37 d	3.32 dd (ca. 3.0, 2.8)	H-7, H-10	NH, Hα-4	C-4,5,7,10	59.55 d	3.37 br s	H-7, H-10	H-2, NH, Hα-4	C-7,10
7	58.63 d	3.24 dd (3.4, 2.8)	H-6, H-9	H_2-11	C-5,6,8,9,11	58.63 d	3.22 br s	H-6, H-9	H_2-11	C-6,8,9
8	66.37 s	—	—	—	—	66.32 s	—	—	—	—
8-OH	—	4.67 [g] br s		H_2-11, Hα-4, Hβ-4	C-7,8,9,11	—	4.67 [g] br s		H_2-11, Hα-4, Hβ-4	C-7,8,9,11
9	58.08 d	3.26 dd (3.4, 2.7)	H-7, H-10	H_2-11	C-5,7,8,10,11	58.29 d	3.27 br s	H-7, H-10	H_2-11	C-7,8,10
10	57.42 d	3.19 dd (3.0, 2.7)	H-6, H-9	H-2, H-3, Hβ-4	C-4,5,6,9	57.81 d	3.18 br s	H-6, H-9	H-3, Hβ-4	C-6,9
11	47.29 t	Ha 3.08 d (17.9)	H_3-13	H-7,9, HO-8, H_3-13	C-7,8,9,12	47.33 t	Ha 3.08 d (17.9)	H_3-13	H-7,9, HO-8, H_3-13	C-7,8,9,12
		Hb 3.04 d (17.9)	H_3-13	H-7,9, HO-8, H_3-13	C-7,8,9,12		Hb 3.04 d (17.9)	H_3-13	H-7,9, HO-8, H_3-13	C-7,8,9,12
12	210.89 s	—	—	—	—	210.86 s	—	—	—	—
13	31.65 q	3H 2.28 s	H_2-11	H_2-11	C-11,12	31.67 q	3H 2.26 s	H_2-11	H_2-11	C-11,12
1'	166.12 s	—	—	—	—	166.16 s	—	—	—	—
2'	121.41 d	5.77 d (15.3)	H-3', H-4'	NH, H-4', H_3-13'	C-1', 3',4'	121.19 d	5.79 d (15.3)	H-3', H-4'	NH, H-4', H_3-13'	C-1',4'
3'	151.30 d	6.72 ddd (15.3, 8.3, 1.3)	H-2', H-4', H_3-13'	H-4', Ha-5', H_3-13'	C-1',2',4',5',13'	151.67 d	6.76 dd (15.3, 8.3)	H-2', H-4', H_3-13'	H-4', Ha-5', H_3-13'	C-1',2',4',5',13'
4'	34.13 d	2.44–2.33 m	H-2',3', H_2-5',H_3-13'	H-2',3', H_2-5', H_3-13', 14'	C-2',3',5',6',13'	34.11 d	2.12–2.04 m	H-2',3', H_2-5',H_3-13'	H-2',3', H_2-5', H_3-13', 14'	C-2',3',5',6',13'
5'	44.05 t	Ha 1.39–1.32 m	H-4', Hb-5', H-6'	H-4'	C-3',4',6',7',13',14'	44.05 t	Ha 1.39–1.32 m	H-4', Hb-5', H-6'	H-4'	C-3',4',6',7',13',14'
		Hb 1.13–1.07 m	H-4', Ha-5', H-6'	H-4'	C-3',4',6',7',13',14'		Hb 1.13–1.07 m	H-4', Ha-5', H-6'	H-4'	C-3',4',6',7',13',14'
6'	30.45 d	1.44–1.35 m	H_2-5', H_2-7', H_3-14'		C-4',5',7',8',14'	30.45 d	1.44–1.35 m	H_2-5', H_2-7', H_3-14'		C-4',5',7',8',14'

Table S1. A: *Cont.*

No.	**1a** (2,3-*cis*)					**1b** (2,3-*trans*)				
	δ_C [b,c]	δ_H [b] (*J* in Hz)	COSY [d]	NOE [e]	HMBC [f]	δ_C [b,c]	δ_H [b] (*J* in Hz)	COSY [d]	NOE [e]	HMBC [f]
7'	37.42 t	Ha 1.27–1.18 m	H-6', Hb-7', H_2-8'		C-5',6',8',9',14'	37.38 t	Ha 1.27–1.18 m	H-6', Hb-7', H_2-8'		C-5',6',8',9',14'
		Hb 1.11–1.05 m	H-6', Ha-7', H_2-8'		C-5',6',8',9',14'		Ha 1.11–1.05 m	H-6', Ha-7', H_2-8'		C-5',6',8',9',14'
8'	26.84 t	2H 1.29–1.21 m	H_2-7', H_2-9'		C-6',7',9',10'	26.84 t	2H 1.29–1.21 m	H_2-7', H_2-9'		C-6',7',9',10'
9'	29.71 t	2H 1.27–1.18 m	H_2-8', H_2-10'		C-7',8',10',11'	29.71 t	2H 1.27–1.18 m	H_2-8', H_2-10'		C-7',8',10',11'
10'	31.93 t	2H 1.29–1.21 m	H_2-9', H_2-11'		C-8',9',11',12'	31.93 t	2H 1.29–1.21 m	H_2-9', H_2-11'		C-8',9',11',12'
11'	22.67 t	2H 1.30–1.26 m	H_2-10', H_3-12'		C-9',10',12'	22.67 t	2H 1.32–1.28 m	H_2-10', H_3-12'		C-9',10',12'
12'	14.08 q	0.88 t (7.0)	H_2-11'		C-10',11'	14.08 q	0.83 t (7.0)	H_2-11'		C-10',11'
13'	20.47 q	1.03 dd (6.6, 1.3)	H-3', H-4'	H-2',3', 4'	C-3',4',5'	20.43 q	1.01 d (6.6)	H-4'	H-2',3', 4'	C-3',4',5'
14'	19.56 q	0.84 d (6.4)	H-6'	H-4'	C-5',6',7'	20.41 q	0.82 d (6.4)	H-6'	H-4'	C-5',6',7'

[a] Signals were assigned on the basis of DEPT, GOESY 1D difference NOE, 2D ^1H–^1H COSY, HMQC, HMBC, NOESY experiments. The ratio of **1a** and **1b** was approximate 5.5:1 (by integral values of their separated NH, H-2, 2-OH, H-6 and H_3-13 signals) in the ^1H-NMR spectrum measured soon after dissolving in $CDCl_3$. However, this ratio increased with disappearance of the 2-OH and 8-OH signals to become approximate 6.3:1 (by integral values of the NH, H-2, H-3, H-4α, H-4β, H-6 and H-10 signals that separated well with the disappearance of the two OH signals) in the ^1H-NMR spectrum measured after over a week in $CDCl_3$. All ^1H signals in this Table were taken from the ^1H-NMR spectrum measured soon after dissolving **1** in $CDCl_3$. [b] Chemical shift values (δ_H and δ_C) were recorded using the internal TMS signals (δ_H and δ_C both 0.00) as references, respectively. [c] Multiplicities of the carbon signals were determined by DEPT and are shown as s (singlet), d (doublet), t (triplet) and q (quartet), respectively. [d] Numbers in each line of this column indicate the protons that correlated with the proton in the corresponding line in ^1H–^1H COSY. [e] Numbers in each line of this column indicate the protons that showed NOE correlations with the proton in the corresponding line in 2D NOESY or 1D GOESY difference NOE experiments. The NOEs between two protons in a spin coupling relationship were detected by the 1D GOESY difference NOE experiments. [f] Numbers in each line of this column indicate the carbons that showed HMBC correlations with the proton in the corresponding line in the HMBC experiments optimized for the 8.3 Hz of long-range J_{CH} value. [g] This OH signal disappeared after over a week of time period in $CDCl_3$. [h] This NH signal came to be δ6.06 br d (J = 8.0 Hz) with the disappearance of OH signals after over a week of time period for various NMR experiments in the $CDCl_3$ solution.

Table S1. B: 600 MHz ^{1}H and 150 MHz ^{13}C-NMR data of **1** in $CDCl_3$ (data from the spectra measured after over a week in $CDCl_3$, **1a**:**1b** = approx. 6.3:1) [a].

No.	**1a (2,3-*cis*)**					**1b (2,3-*trans*)**				
	δ_C [b,c]	δ_H [b] (*J* in Hz)	COSY [d]	NOE [e]	HMBC [f]	δ_C [b,c]	δ_H [b] (*J* in Hz)	COSY [d]	NOE [e]	HMBC [f]
2	96.45 s	5.55 t (4.3)	H-3, NH, Hα-4	2-OH, H-3,10, NH, Hβ-4	C-3,4,5	102.61 s	5.57 br d (3.7)	H-3, Hα-4	2-OH, H-3,6, NH, Hα-4	C-3,4,5
2-OH	—	4.98 [g] br s		H-2		—	4.80 [g] br s		H-2	
3	52.11 d	4.72 dtd (10.7, 8.4, 4.3)	H-2, NH, H_2-4	H-2, NH, Hβ-4, H-10	C-2,4,1'	57.21 d	4.59 m	H-2, NH, H_2-4	H-2, NH, Hβ-4, H-10	C-2,5,1'
NH	—	6.06 br d (8.0)	H-2, H-3	H-2,3,6, Hα,β-4, H-2'	C-2,3,1',2'	—	5.95 br s	H-3	H-2,3,6, Hα,β-4, H-2'	
4	37.06 t	Hβ 2.58 dd (12.9, 8.4)	H-3, Hα-4	H-2,3, NH, Hα-4, H-10	C-2,3,6,10	38.50 t	Hβ 2.63 br dd (12.5, 6.2)	H-3, Hα-4	H-3, NH, Hα-4, H-10	C-2,3,5,6,10
		Hα 2.00 ddd (12.9, 10.7, 4.3)	H-2, H-3, Hβ-4	NH, Hβ-4, H-6	C-3,5,6,10		Hα 2.16 br d (12.5)	H-2, H-3, Hβ-4	H-2, NH, Hβ-4, H-6	C-2,3,6,10
5	79.05 s	—	—	—	—	80.45 s	—	—	—	—
6	59.37 d	3.31 dd (3.2, 2.6)	H-7, H-10	NH, Hα-4	C-4,5,7,10	59.55 d	3.36 br s	H-7, H-10	H-2, NH, Hα-4	C-7,10
7	58.63 d	3.25 dd (3.3, 2.5)	H-6, H-9	H_2-11	C-5,6,8,9,11	58.63 d	3.25 dd (3.2, 2.6)	H-6, H-9	H_2-11	C-6,8,9
8	66.37 s	—	—	—	—	66.32 s	—	—	—	—
8-OH	—	4.67 [g] br s		H_2-11	C-7,8,9,11	—	4.67 [g] br s		H_2-11	C-7,8,9,11
9	58.08 d	3.27–3.23 m	H-7, H-10	H_2-11	C-5,7,8,10,11	58.29 d	3.27-3.23 m	H-7, H-10	H_2-11	C-7,8,10
10	57.42 d	3.19 dd (3.2, 2.5)	H-6, H-9	H-2, H-3, Hβ-4	C-4,5,6,9	57.81 d	3.16 br s	H-6, H-9	H-3, Hβ-4	C-6,9
11	47.29 t	Ha 3.09 d (18.1)	H_3-13	H-7,9, HO-8, H_3-13	C-7,8,9,12	47.33 t	Ha 3.09 d (18.1)	H_3-13	H-7,9, HO-8, H_3-13	C-7,8,9,12
		Hb 3.05 d (18.1)	H_3-13	H-7,9, HO-8, H_3-13	C-7,8,9,12		Hb 3.05 d (18.1)	H_3-13	H-7,9, HO-8, H_3-13	C-7,8,9,12
12	210.89 s	—	—	—	—	210.86 s	—	—	—	—
13	31.65 q	3H 2.28 s	H_2-11	H_2-11	C-11,12	31.67 q	3H 2.26 s	H_2-11	H_2-11	C-11,12
1'	166.12 s	—	—	—	—	166.16 s	—	—	—	—
2'	121.41 d	5.76 d (15.3)	H-3', H-4'	NH, H-4', H_3-13'	C-1', 3',4'	121.19 d	5.74 d (15.0)	H-3', H-4'	NH, H-4', H_3-13'	C-1',4'
3'	151.30 d	6.73 br dd (15.3, 8.3)	H-2', H-4', H_3-13'	H-4', Ha-5', H_3-13'	C-1',2',4',5',13'	151.67 d	6.74 br dd (15.0, 8.0)	H-2', H-4', H_3-13'	H-4', Ha-5', H_3-13'	C-1',2',4',5',13'
4'	34.13 d	2.44–2.33 m	H-2',3', H_2-5',H_3-13'	H-2',3', H_2-5', H_3-13', 14'	C-2',3',5',6',13'	34.11 d	2.12–2.04 m	H-2',3', H_2-5',H_3-13'	H-2',3', H_2-5', H_3-13', 14'	C-2',3',5',6',13'
5'	44.05 t	Ha 1.39–1.33 m	H-4', Hb-5', H-6'	H-4'	C-3',4',6',7',13',14'	44.05 t	Ha 1.39–1.33 m	H-4', Hb-5', H-6'	H-4'	C-3',4',6',7',13',14'
		Hb 1.14–1.07 m	H-4', Ha-5', H-6'	H-4'	C-3',4',6',7',13',14'		Hb 1.14–1.07 m	H-4', Ha-5', H-6'	H-4'	C-3',4',6',7',13',14'
6'	30.45 d	1.44–1.36 m	H_2-5', H_2-7', H_3-14'		C-4',5',7',8',14'	30.45 d	1.44–1.36 m	H_2-5', H_2-7', H_3-14'		C-4',5',7',8',14'

Table S1. B: *Cont.*

No.	**1a (2,3-*cis*)**					**1b (2,3-*trans*)**				
	δ_C [b,c]	δ_H [b] (*J* in Hz)	COSY [d]	NOE [e]	HMBC [f]	δ_C [b,c]	δ_H [b] (*J* in Hz)	COSY [d]	NOE [e]	HMBC [f]
7'	37.42 t	Ha 1.27–1.18 m	H-6', Hb-7', H_2-8'		C-5',6',8',9',14'	37.38 t	Ha 1.27–1.18 m	H-6', Hb-7', H_2-8'		C-5',6',8',9',14'
		Hb 1.11–1.05 m	H-6', Ha-7', H_2-8'		C-5',6',8',9',14'		Ha 1.11–1.05 m	H-6', Ha-7', H_2-8'		C-5',6',8',9',14'
8'	26.84 t	2H 1.28–1.21 m	H_2-7', H_2-9'		C-6',7',9',10'	26.84 t	2H 1.28–1.21 m	H_2-7', H_2-9'		C-6',7',9',10'
9'	29.71 t	2H 1.27–1.18 m	H_2-8', H_2-10'		C-7',8',10',11'	29.71 t	2H 1.27–1.18 m	H_2-8', H_2-10'		C-7',8',10',11'
10'	31.93 t	2H 1.28–1.21 m	H_2-9', H_2-11'		C-8',9',11',12'	31.93 t	2H 1.28–1.21 m	H_2-9', H_2-11'		C-8',9',11',12'
11'	22.67 t	2H 1.31–1.26 m	H_2-10', H_3-12'		C-9',10',12'	22.67 t	2H 1.32–1.28 m	H_2-10', H_3-12'		C-9',10',12'
12'	14.08 q	0.88 t (7.0)	H_2-11'		C-10',11'	14.08 q	0.83 t (7.0)	H_2-11'		C-10',11'
13'	20.47 q	1.03 dd (6.6, 0.8)	H-3', H-4'	H-2',3', 4'	C-3',4',5'	20.43 q	1.01 d (6.6)	H-4'	H-2',3', 4'	C-3',4',5'
14'	19.56 q	0.84 d (6.4)	H-6'	H-4'	C-5',6',7'	20.41 q	0.82 d (6.4)	H-6'	H-4'	C-5',6',7'

[a] Signals were assigned on the basis of DEPT, GOESY 1D difference NOE, 2D ^{1}H–^{1}H COSY, HMQC, HMBC, and NOESY experiments. The approximate 6.3:1 ratio of **1a**:**1b** was calculated using the integral values of their NH, H-2, H-3, H-4α, H-4β, H-6 and H-10 signals in the ^{1}H-NMR spectrum measured after over a week of time period in $CDCl_3$. The ^{1}H signals in this Table except for the signals of 2-OH and 8-OH were from the ^{1}H-NMR spectrum measured after over a week of time period in $CDCl_3$, where the 2-OH and 8-OH proton signals have already disappeared. [b] Chemical shift values (δ_H and δ_C) were recorded using the internal TMS signals (δ_H and δ_C both 0.00) as references, respectively. [c] Multiplicities of the carbon signals were determined by DEPT experiments and are shown as s (singlet), d (doublet), t (triplet) and q (quartet), respectively. [d] Numbers in each line of this column indicate the protons that correlated with the proton in the corresponding line in ^{1}H–^{1}H COSY. [e] Numbers in each line of this column indicate the protons that showed NOE correlations with the proton in the corresponding line in NOESY or 1D difference NOE experiments. The NOEs between two protons in a spin coupling relationship were detected by the 1D difference NOE experiments. [f] Numbers in each line of this column indicate the carbons that showed HMBC correlations with the proton in the corresponding line in the HMBC experiments optimized for the 8.3 Hz of long-range J_{CH} value. [g] This OH signal was from the ^{1}H-NMR spectrum measured soon after dissolving samples in $CDCl_3$, which disappeared after over a week of time period in the $CDCl_3$ solution.

Table S2. 400 MHz ^{1}H and 100 MHz ^{13}C-NMR data of **1** in CD_3OD (**1a**:**1b** = approx. 1.8:1) [a].

No.	**1a** (2,3-*cis*)					**1b** (2,3-*trans*)				
	δ_C [b,c]	δ_H [b] (*J* in Hz)	COSY [d]	NOE [e]	HMBC [f]	δ_C [b,c]	δ_H [b] (*J* in Hz)	COSY [d]	NOE [e]	HMBC [f]
2	97.71 s	5.43 d (4.4)	H -3	H-3, H-10, H-2'	C-3,4,5	103.98 s	5.47 d (2.8)	H -3	H-3, Hα-4, H-6, H-2'	C-3,4,5
3	53.98 d	4.54 ddd (11.6, 8.4, 4.4)	H-2, H_2-4	H-2, Hβ-4, H-10, H-2'	C-2,4,1'	59.06 d	4.34 br d (6.8)	H-2, H_2-4	H-2, Hαβ-4, H-10, H-2'	C2,5,1'
4	37.20 t	Hβ 2.40 dd (12.8, 8.4)	H-3, Hα-4	H-3, Hα-4, H-10	C-2,3,6,10	39.38 t	Hβ 2.55 dd (14.4, 6.8)	H-3, Hα-4	H-3, Hα-4, H-10	C-2,3,5,5,10
		Hα 2.10 dd (12.8, 11.6)	H-3, Hβ-4	Hβ-4, H-6	C-3,5,6,10		Hα 2.07 dd (14.4, 1.6)	H-3, Hβ-4	H-2, H-3, Hβ-4, H-6	C-2,3,6,10
5	79.73 s	—	—	—	—	81.82 s	—	—	—	—
6	61.36 d	3.33–3.30	H-7, H-10	Hα-4	C-5,7,8,10	59.49 d	3.25 dd (3.4, 2.5)	H-7, H-10	H-2, Hα-4	C-5,7,8,10
7	60.03 d	3.31–3.30	H-6, H-9	H_2-11, H_3-13	C-5,6,8,9	59.99 d	3.29–3.26	H-6, H-9	H_2-11, H_3-13	C-5,6,8,9

Table S2. *Cont.*

No.	**1a** (2,3-*cis*)					**1b** (2,3-*trans*)				
	δ_C [b,c]	δ_H [b] (*J* in Hz)	COSY [d]	NOE [e]	HMBC [f]	δ_C [b,c]	δ_H [b] (*J* in Hz)	COSY [d]	NOE [e]	HMBC [f]
8	67.39 s	—	—	—	—	67.39 s	—	—	—	—
9	60.37 d	3.31–3.30	H-7, H-10	H_2-11, H_3-13	C-5,7,8,10	60.37 d	3.29–3.26	H-7, H-10	H_2-11, H_3-13	C-5,7,8,10
10	59.32 d	3.20 dd (3.6, 2.2)	H-6, H-9	H-2, H-3, Hβ-4	C-5,6,9	61.46 d	3.35–3.31	H-6, H-9	H-3, Hβ-4	C-5,6,9
11	50.26 t	2H 2.98 br s	H_3-13	H-7, H-9	C-7,8,9,12,13	50.26 t	2H 2.98 br s	H_3-13	H-7, H-9	C-7,8,9,12,13
12	209.89 s	—	—	—	—	209.89 s	—	—	—	—
13	33.01 q	3H 2.26 s	H_2-11	H-7, H-9	C-11,12	33.04 q	3H 2.26 s	H_2-11	H-7, H-9	C-11,12
1'	169.08 s	—	—	—	—	169.08 s	—	—	—	—
2'	123.36 d	6.01 dd (15.4, 0.8)	H-3', H-4'	H-2, H-3, H-4'	C-1', 4'	123.15 d	5.97 d (15.4)	H-3', H-4'	H-2, H-3, H-4'	C-1', 4'
3'	152.27 d	6.657 dd (15.4, 8.6)	H-2', H-4'		C-1',2',4',5',13'	152.40 d	6.665 dd (15.4, 8.6)	H-2', H-4'		C-1',2',4',5',13'
4'	35.92 d	2.48–2.38 m	H-2',3', H_2-5',H_3-13'	H-2',5',6', H_3-13', 14'	C-3', 5'	35.92 d	2.48–2.38 m	H-2',3', H_2-5',H_3-13'	H-2',5',6', H_3-13', 14'	C-3', 5'
5'	45.73 t	Ha 1.18–1.09 m	H-4', Hb-5', H-6'	H-4'	C-3',6',7'	45.73 t	Ha 1.18–1.09 m	H-4', Hb-5', H-6'	H-4'	C-3',6',7'
		Hb 1.45–1.35 m	H-4', Ha-5', H-6'	H-4'	C-3',6′ ,7'		Hb 1.45–1.35 m	H-4', Ha-5', H-6'	H-4'	C-3',6′ ,7'
6'	32.09 d	1.45–1.35 m	H_2-5', H_2-7', H_3-14'	H-4'	C-4',5',7',8',14'	32.09 d	1.45–1.35 m	H_2-5', H_2-7', H_3-14'	H-4'	C-4',5',7',8',14'
7'	39.15 t	Ha 1.18–1.09 m	H-6', Hb-7', H_2-8'		C-6',8',9',14'	39.07 t	Ha 1.18–1.09 m	H-6', Hb-7', H_2-8'		C-6',8',9',14'
		Hb 1.33–1.27 m	H-6', Ha-7', H_2-8'		C-6',8',9',14'		Ha 1.33–1.27 m	H-6', Ha-7', H_2-8'		C-6',8',9',14'
8'	31.23 t	2H 1.31–1.22 m	H_2-7', H_2-9'		C-6',7',9',10'	31.23 t	2H 1.31–1.22 m	H_2-7', H_2-9'		C-6',7',9',10'
9'	28.47 t	2H 1.31–1.22 m	H_2-8', H_2-10'		C-7',8',10',11'	28.47 t	2H 1.31–1.22 m	H_2-8', H_2-10'		C-7',8',10',11'
10'	33.59 t	2H 1.31–1.22 m	H_2-9', H_2-11'		C-8',9',11',12'	33.59 t	2H 1.31–1.22 m	H_2-9', H_2-11'		C-8',9',11',12'
11'	24.23 t	2H 1.36–1.29 m	H_2-10', H_3-12'		C-9',10',12'	24.23 t	2H 1.36–1.29 m	H_2-10', H_3-12'		C-9',10',12'
12'	14.97 q	0.899 t (6.7)	H_2-11'		C-10',11'	14.97 q	0.878 t (6.7)	H_2-11'		C-10',11'
13'	21.60 q	1.045 d (6.7)	H-4'	H-4'	C-3',4',5'	21.53 q	1.039 d (6.7)	H-4'	H-4'	C-3',4',5'
14'	20.25 q	0.873 d (6.4)	H-6'	H-4'	C-5',6',7'	20.29 q	0.865 d (6.4)	H-6'	H-4'	C-5',6',7'

[a] The ^1H and ^{13}C-NMR signals were assigned on the basis of DEPT, ^1H–^1H COSY, HMQC, HMBC, NOESY, and 1D difference NOE experiments. The approximate 1.8:1 ratio of **1a**:**1b** was calculated using integral values of their H-2 and H-3 signals. [b] Chemical shift values (δ_H and δ_C) were recorded using the solvent signals (CD_3OD: δ_H 3.31/δ_C 49.50) as references, respectively. [c] Multiplicities of the carbon signals were determined by DEPT experiments and are shown as s (singlet), d (doublet), t (triplet) and q (quartet), respectively. [d] Numbers in each line of this column indicate the protons that correlated with the proton in the corresponding line in ^1H–^1H COSY. [e] Numbers in each line of this column indicate the protons that showed NOE correlations with the proton in the corresponding line in NOESY or 1D difference NOE experiments. The NOEs between two protons in a spin coupling relationship were detected by the 1D difference NOE experiments. [f] Numbers in each line of this column indicate the carbons that showed HMBC correlations with the proton in the corresponding line in the HMBC experiments optimized for the 8.3 Hz of long-range J_{CH} value.

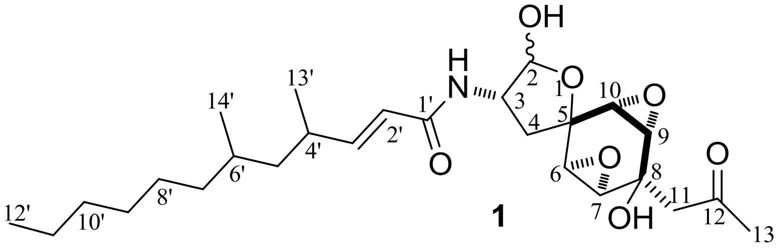

1

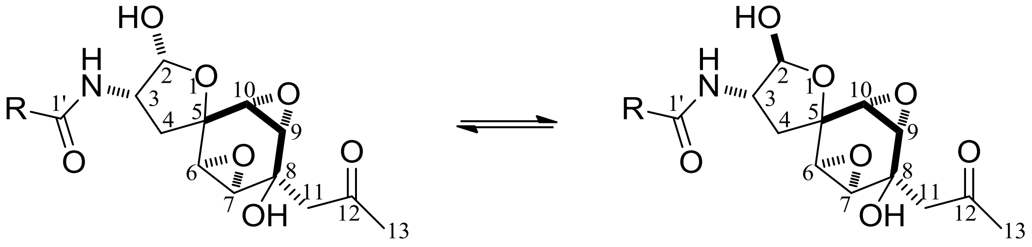

1a **1b**

The *cis-trans* isomerism of **1** in solution

The ratio of **1a** and **1b** is approximate 1.8:1 in CD_3OD and 5.5:1→6.3:1 in $CDCl_3$)

Data for Calculating Ratio of **1a** and **1b** in $CDCl_3$ Solution.

**[1]H Signal**	**Integral value**		**Ratio (1a:1b)**
	**1a**	**1b**	
NH	5.48	1.01	5.43:1
H-2	5.46	1.00	5.46:1
HO-2	5.28	0.96	5.50:1
H-6	5.56	1.01	5.50:1
H_3-13	15.11	2.76	5.47:1
**Mean Ratio (1a:1b)**			**5.5:1**

The data in this table were taken in the ^{1}H-NMR spectrum measured soon after dissolving **1** in $CDCl_3$.

Data for Calculating Ratio of **1a** and **1b** in $CDCl_3$ Solution.

**[1]H Signal**	**Integral value**		**Ratio (1a:1b)**
	**1a**	**1b**	
H-3'(**1a**+**1b**)	1.00		—
NH	0.80	0.12	6.67:1
H-3	0.94	0.17	5.53:1
H-4β	0.80	0.12	6.67:1
H-4α	0.89	0.16	5.56:1
H-6	0.80	0.12	6.67:1
H-10	0.80	0.12	6.67:1
**Mean Ratio (1a:1b)**			**6.3:1**

The data in this table were taken in the ^{1}H-NMR spectrum measured after over a week of time period in $CDCl_3$. The **1a**:**1b** ratio increased with the disappearance of 2-OH and 8-OH signals to become approximate 6.3:1 (by integral values of the given proton signals that separated well with the disappearance of the two OH signals) in the spectrum.

Data for Calculating Ratio of **1a** and **1b** in CD_3OD Solution.

^1H Signal	Integral value		Ratio (1a:1b)
	1a	1b	
H-2	1.78	1.00	1.8:1
H-3	1.83	1.00	1.8:1
**Mean Ratio (1a:1b)**			**1.8:1**

Table S3. 600 MHz ^1H and 150 MHz ^{13}C-NMR data of **2** in $CDCl_3$ [a].

Position	δ_C [b,c]	δ_H (J in Hz) [b]	COSY [d]	NOE [e]	HMBC [f]
1	167.9 s	—	—	—	—
2 (NH)	—	5.702 s	H-3	H-3, H_2-12, H-14,18	C-1, C-3, C-4, C-11a
3	56.1 d	4.30 ddd (9.3, 3.3, 1.4)	H-2, H_2-12, H-11a	H-2,11a,10', H_2-12, H-14,18	C-1, C-4, C-12, C-13
4	164.1 s	—	—	—	—
5 (N)	—	—	—	—	—
5a	79.8 d	5.96 br s		H-2', H-19a, H-19b	C-1',4,6a,10a,10b, 11,11a,19
6 (N)	—	—	—	—	—
6a	148.1 s	—	—	—	—
7	108.8 d	6.71 d (7.5)	H-8,9	H-2', H-3', H-10'	C-9, C-10a
8	128.8 d	7.10 td (7.5, 0.8)	H-7,9,10		C-6a, C-10
9	119.8 d	6.78 t (7.5)	H-7,8,10		C-7, C-10a
10	125.3 d	7.13 br d (7.5)	H-8,9	Hα-11, H-20	C-6a, C-8, C-10b,
10a	130.0 s	—	—	—	—
10b	60.7 s	—	—	—	—
11	38.4 t	Hα 2.45 dd (12.2, 5.5)	Hβ-11, H-11a	H-10, Hβ-11, H-11a	C-5a,C-10a, C-10b, C-11a
		Hβ 2.14 dd (12.2, 11.6)	Hα-11, H-11a	Hα-11, H-19a, H-19b, H-20	C-1, C-10a, C-10b, C-11a
11a	58.6 d	3.95 ddd (11.6, 5.5, 1.4)	H-3, Hα-11, Hβ-11	H-3, Hα-11	C-1, C-4, C-11
12	37.5 t	Ha 3.37 dd (14.4, 3.3)	H-3, Hb-12, H-14,18	NH, H-3, H-14,18	C-3, C-4, C-13, C-14,18
		Hb 2.90 dd (14.4, 9.3)	H-3, Ha-12, H-14,18	NH, H-3, H-14,18	C-3, C-4, C-13, C-14,18
13	135.4 s	—	—	—	—
14	129.2 d	7.17 br d (7.2)	H_2-12, H-15,16	NH, H-3, H_2-12	C-12, C-16, C-18
15	129.3 d	7.31 br t (7.2)	H-14,16,17		C-13, C-17
16	127.6 d	7.26 br t (7.2)	H-14,15,17,18		C-14, C-18
17	129.3 d	7.31 br t (7.2)	H-15,16,18		C-13, C-15
18	129.2 d	7.17 br d (7.2)	H_2-12, H-16,17	NH, H-3, H_2-12	C-12, C-14, C-16
19	41.1 s	—	—	—	—
19a	22.2 q	1.03 s		H-5a, H-11β, H-20	C-10b, C-19, C-19b, C-20
19b	23.0 q	0.96 s		H-5a, H-11β, H-20	C-10b, C-19, C-19a, C-20
20	143.4 d	5.90 dd (17.3, 10.9)	H*cis*-21, H*trans*-21	H-10, Hβ-11, H-19a,19b	C-10b, C-19, C-19a, C-19b
21	114.6 t	H*cis* 5.11 d (10.9)	H-20		C-10b, C-19, C-20
		H*trans* 5.06 d (17.3)	H-20		C-10b, C-19, C-20
2'	89.9 d	5.69 d (9.5)	H-3'	3'-NH, Hα-4', H-6',5a,7,3''	C-3', C-4', C-5', C-5a, C-6a
3'	49.7 d	5.55 br s	H-2', Hα-4', Hβ-4'	Hβ-4', H-7	
4'	38.2 t	Hα 2.12 dd (13.5, 9.6)	H-3', Hβ-4'	H-2', Hβ-4', H-6'	C-3', C-5', C-6', C-10'
		Hβ 2.76 dd (13.5, 8.8)	H-3', Hα-4'	H-3', Hα-4', H-10'	C-2', C-3', C-5', C-6', C-10'
5'	74.7 s	—	—	—	—
6'	57.4 d	3.40 dd (3.3, 2.7)	H-7',10'	H-2', Hα -4', H-7'	C-4', C-5', C-7', C-10'

Table S3. *Cont.*

Position	δ_C [b,c]	δ_H (J in Hz) [b]	COSY [d]	NOE [e]	HMBC [f]
7'	58.3 d	3.29 dd (3.4, 3.3)	H-6',9'	H-6', 8'-OH, H_2-11', H_3-13'	C-5', C-6', C-8', C-9', C-11'
8'	66.4 s	—	—	—	—
9'	57.9 d	3.23 dd (3.4, 3.2)	H-7',10'	8'-OH, H-10', H_2-11', H_3-13'	C-5', C-7', C-8', C-10', C-11'
10'	57.6 d	3.50 dd (3.2, 2.7)	H-6',9'	H-3, H-3', Hβ-4', H-9', H-7	C-4', C-5', C-6', C-9'
11'	47.1 t	2H 3.07 s	8'-OH, H_3-13'	8'-OH, H-7',9', H_3-13'	C-7', C-8', C-9', C-12'
12'	211.2 s	—	—	—	—
13'	31.7 q	3H 2.27 s	H_2-11'	H-7',9', H_2-11'	C-11', C-12', C-8'(week)
1"	166.2 s	—	—	—	—
2"	121.2 d	5.70 br d (15.3)	H-3", H-4"	H-4", H_3-13"	C-1", C-3", C-4"
3"	151.4 d	6.73 dd (15.3, 8.4)	H-2", H-4"	H_3-13"	C-1", C-4", C-5", 13"
4"	34.1 d	2.40–2.31 m	H-2",3", H_2-5",H_3-13"	H-2"	C-2", C-3", C-5", 13"
5"	44.0 t	Ha 1.34–1.30 m	H-4", H_2-6"		C-3", C-4", C-6", C-7", 13", 14"
		Hb 1.12–1.06 m	H-4", H_2-6"		C-3", C-4", C-6", C-7", 13", 14"
6"	30.3 d	2H 1.42–1.34 m	H_2-5", H_2-7", H_3-14"		C-7", C-8"
7"	37.4 t	Ha 1.26–1.15 m	H_2-6", Hb-7", H_2-8"		C-5", C-6", C-9"
		Hb 1.08–1.03 m	H_2-6", Ha-7", H_2-8"		C-6", C-8"
8"	26.8 t	2H 1.26–1.15 m	H_2-7", H_2-9"		C-7", C-9"
9"	29.7 t	2H 1.26–1.15 m			C-8", C-10"
10"	31.9 t	2H 1.26–1.15 m			C-8", C-9", C-11"
11"	22.6 t	2H 1.30–1.24 m	H_2-10", H_3-12"		C-9", C-10", C-12"
12"	14.1 q	3H 0.87 t (7.1)	H_2-11"		C-10", C-11"
13"	20.4 q	3H 0.99 d (6.7)	H-4"	H-2", H-3"	C-3", C-4", C-5"
14"	19.4 q	3H 0.81 d (6.5)	H-6"		C-5", C-6", C-7"
3'-NH	—	6.13 br s		H-2'	
8'-OH	—	4.63 s	H_2-11'	H-7',9', H_2-11'	C-8', C-7', C-9', C-11'

[a] ^{1}H and ^{13}C-NMR signals were assigned on the basis of DEPT, GOESY 1D difference NOE, 2D ^{1}H–^{1}H COSY, HMQC, HMBC, NOESY and ROESY experiments. [b] Chemical shift values (δ_H and δ_C) were recorded using the internal TMS signals (δ_H and δ_C both 0.00) as references, respectively. [c] Multiplicities of the carbon signals were determined by DEPT experiments and are shown as s (singlet), d (doublet), t (triplet) and q (quartet), respectively. [d] Numbers in each line of this column indicate the protons that correlated with the proton in the corresponding line in ^{1}H–^{1}H COSY. [e] Numbers in each line of this column indicate the protons that showed NOE correlations with the proton in the corresponding line in 2D NOESY or ROESY experiments or in 1D GOESY difference NOE experiments. The NOEs between two protons in a spin coupling relationship were detected by the GOESY 1D difference NOE experiments. [f] Numbers in each line of this column indicate the carbons that showed HMBC correlations with the proton in the corresponding line in the HMBC experiments optimized for the 4, 6, 8.3, or 12 Hz of long-range J_{CH} value.

Table S4. 600 MHz ^{1}H and 150 MHz ^{13}C-NMR data of **3** in $CDCl_3$ [a].

Position	δ_C [b,c]	δ_H (J in Hz) [b]	COSY [d]	NOE [e]	HMBC [f]
1	168.2 s	—	—	—	—
2 (NH)	—	5.99 br s	H-3	H-3, H_2-12, H-14,18	C-1, C-3, C-4, C-11a
3	56.2 d	4.22 ddd (8.7, 3.4, 1.3)	H-2, H_2-12, H-11a	H-2,11a,10', H_2-12, H-14,18	C-1, C-4, C-12, C-13
4	164.1 s	—	—	—	—
5 (N)	—	—	—	—	—
5a	79.4 d	5.96 br s		H-2', H_3-19a, H_3-19b	C-4, 6a, 10a, 10b, 11a, 19
6 (N)	—	—	—	—	—
6a	148.2 s	—	—	—	—
7	108.6 d	6.70 d (7.8)	H-8,9	H-2', H-3', H-10'	C-9, C-10a
8	128.8 d	7.10 td (7.8, 0.9)	H-7,9,10		C-6a, C-10
9	119.8 d	6.77 t (7.8)	H-7,8,10		C-7, C-10a
10	125.3 d	7.13 br d (7.8)	H-8,9	Hα-11, H-20, Hβ-11 (very weak)	C-6a, C-8, C-10b,
10a	130.0 s	—	—	—	—
10b	60.7 s	—	—	—	—
11	38.5 t	Hα 2.42 dd (12.3, 5.5)	Hβ-11, H-11a	H-10, Hβ-11, H-11a	C-5a,C-10a, C-10b, C-11a
		Hβ 2.089 dd (12.3, 11.5)	Hα-11, H-11a	H-10,5a,20, Hα-11, H_3-19a,19b	C-1, C-10a, C-10b, C-11a
11a	58.3 d	3.92 ddd (11.5, 5.5, 1.3)	H-3, Hα-11, Hβ-11	H-2, H-3, Hα-11	C-1, C-11
12	36.7 t	Ha 3.20 dd (14.4, 3.4)	H-3, Hb-12	NH, H-3, H-14,18	C-3, C-4, C-13, C-14,18
		Hb 2.86 dd (14.4, 8.7)	H-3, Ha-12	NH, H-3, H-14,18	C-3, C-4, C-13, C-14,18
13	126.6 s	—	—	—	—
14	130.5 d	6.99 d (8.6)	H-15	NH, H-3, H_2-12	C-12, C-15, C-16, C-18
15	116.1 d	6.76 d (8.6)	H-14		C-13, C-14, C-16, C-17
16	155.5 d	—	—	—	
17	116.1 d	6.76 d (8.6)	H-18		C-13, C-15, C-16, C-18
18	130.5 d	6.99 d (8.6)	H-17	NH, H-3, H_2-12	C-12, C-14, C-16, C-17
19	41.1 s	—	—	—	—
19a	22.2 q	1.02 s	H_3-19b	H-5a, Hβ-11, H-20	C-10b, C-19, C-19b, C-20
19b	23.0 q	0.94 s	H_3-19a	H-5a, Hβ-11, H-20	C-10b, C-19, C-19a, C-20
20	143.4 d	5.88 dd (17.4, 10.7)	H*cis*-21, H*trans*-21	H-10, Hα-11, Hβ-11, H_3-19a,19b	C-10b, C-19, C-19a,19b
21	114.6 t	H*cis* 5.10 d (10.7)	H-20		C-10b, C-19, C-20
		H*trans* 5.04 d (17.4)	H-20		C-10b, C-19, C-20
2'	89.8 d	5.67 d (9.0)	H-3', Hα-4', Hβ-4'	3'-NH, Hα-4', H-6', H-5a, H-7	C-3', C-4', C-5a, C-6a
3'	49.5 d	5.59 br s	H-2', Hα-4', Hβ-4'	Hβ-4', H-10', H-7	
4'	38.1 t	Hα 2.096 dd (13.3, 9.7)	H-2', H-3', Hβ-4'	H-2', Hβ-4', H-6'	C-3', C-5', C-6', C-10'
		Hβ 2.69 dd (13.3, 8.5)	H-2', H-3', Hα-4'	H-3', Hα-4', H-10'	C-2', C-3', C-6', C-10'
5'	74.6 s	—	—	—	—
6'	57.4 d	3.38 dd (3.3, 2.7)	H-7',10'	H-2', Hα-4', H-7'	C-4', C-5', C-7', C-10'
7'	58.3 d	3.28 dd (3.4, 3.3)	H-6',9'	H-6', H_2-11', H_3-13' (weak)	C-6', C-8', C-9'
8'	66.4 s	—	—	—	—
9'	57.9 d	3.22 dd (3.4, 3.2)	H-7',10'	H-10', H_2-11', H_3-13' (weak)	C-7', C-8', C-10'
10'	57.6 d	3.47 dd (3.2, 2.7)	H-6',9'	H-3, H-3', Hβ-4', H-9', H-7	C-5', C-6', C-9'
11'	47.1 t	2H 3.06 s	H_3-13'	H-7',9', H_3-13'	C-7', C-8', C-9', C-12'

Table S4. *Cont.*

Position	δ_C [b,c]	δ_H (*J* in Hz) [b]	COSY [d]	NOE [e]	HMBC [f]
12'	211.1 s	—	—	—	—
13'	31.7 q	3H 2.26 s	H_2-11'	H-7',9', H_2-11'	C-11', C-12'
1"	166.4 s	—	—	—	—
2"	121.1 d	5.70 br d (15.2)	H-3",4"	H-4", H_3-13"	C-1", C-3", C-4"
3"	151.6 d	6.73 dd (15.2, 8.2)	H-2", H-4"	H_3-13"	C-1", C-4", C-5", 13"
4"	34.1 d	2.40–2.31 m	H-2",3", H_2-5",H_3-13"	H-2"	C-2", C-3", C-5", 13"
5"	44.0 t	Ha 1.34–1.29 m	H-4", H_2-6"		C-3",4",6",7", C-13",14"
		Hb 1.12–1.06 m	H-4", H_2-6"		C-3",4",6",7", C-13",14"
6"	30.4 d	2H 1.41–1.32 m	H_2-5", H_2-7", H_3-14"		C-5", C-7", C-8"
7"	37.4 t	Ha 1.26–1.15 m	H_2-6", Hb-7", H_2-8"		C-5", C-6", C-9"
		Hb 1.08–1.03 m	H_2-6", Ha-7", H_2-8"		C-6", C-8", C-9"
8"	26.8 t	2H 1.26–1.15 m	H_2-7", H_2-9"		C-6", C-7", C-9"
9"	29.7 t	2H 1.26–1.15 m			C-8", C-10"
10"	31.9 t	2H 1.26–1.15 m			C-8", C-9", C-11"
11"	22.6 t	2H 1.30–1.24 m	H_2-10", H_3-12"		C-9", C-10", C-12"
12"	14.1 q	3H 0.87 t (7.0)	H_2-11"		C-10", C-11"
13"	20.4 q	3H 0.98 d (6.7)	H-4"	H-2", H-3"	C-3", C-4", C-5"
14"	19.4 q	3H 0.81 d (6.4)	H-6"		C-5", C-6", C-7"
3'-NH	—	6.21 br s		H-2'	
8'-OH	—	4.66 br s		H-7',9', H_2-11'	C-8', C-7', C-9', C-11'
16-OH	—	6.55 br s		H-15,17	

[a] ^{1}H and ^{13}C-NMR signals were assigned on the basis of DEPT, GOESY 1D difference NOE, 2D ^{1}H–^{1}H COSY, HMQC, HMBC and ROESY experiments. [b] Chemical shift values (δ_H and δ_C) were recorded using the internal TMS signals (δ_H and δ_C both 0.00) as references, respectively. [c] Multiplicities of the carbon signals were determined by DEPT experiments and are shown as s (singlet), d (doublet), t (triplet) and q (quartet), respectively. [d] Numbers in each line of this column indicate the protons that correlated with the proton in the corresponding line in ^{1}H–^{1}H COSY. [e] Numbers in each line of this column indicate the protons that showed NOE correlations with the proton in the corresponding line in 2D ROESY or 1D GOESY difference NOE experiments. The NOEs between two protons in a spin coupling relationship were detected by the GOESY 1D difference NOE experiments. [f] Numbers in each line of this column indicate the carbons that showed HMBC correlations with the proton in the corresponding line in the HMBC experiments optimized for the 8.3 Hz of long-range J_{CH} value.

Figure S1. Phenotypes of the parent strain G59 and its mutants growing on PDA plates by incubation at 28 ℃ for 5 days.

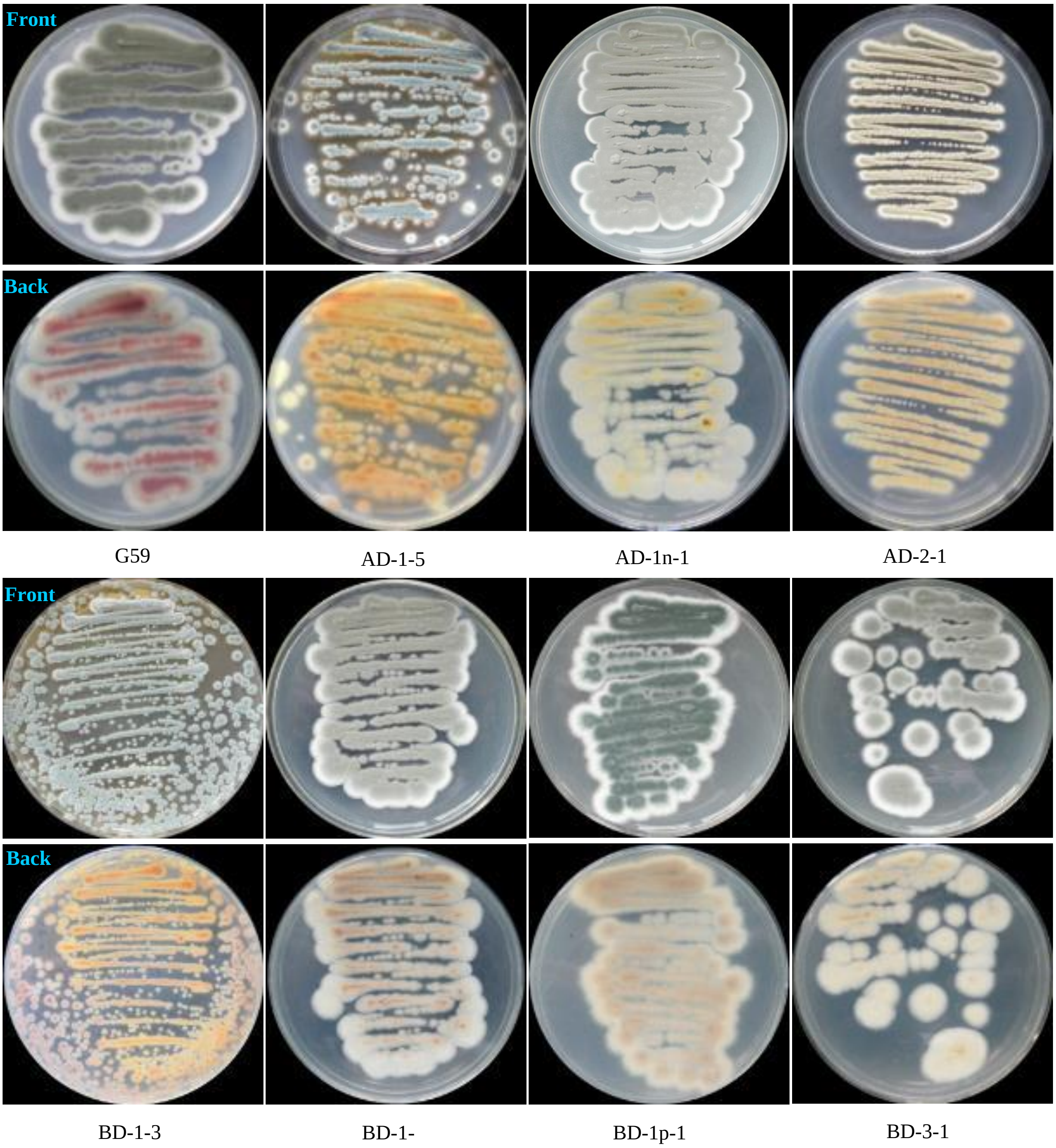

Figure S2 A. HPLC-PDAD-UV analysis of the EtOAc extracts of the control G59 strain and selected mutants. **A**: HPLC profiles detected at 210 nm.

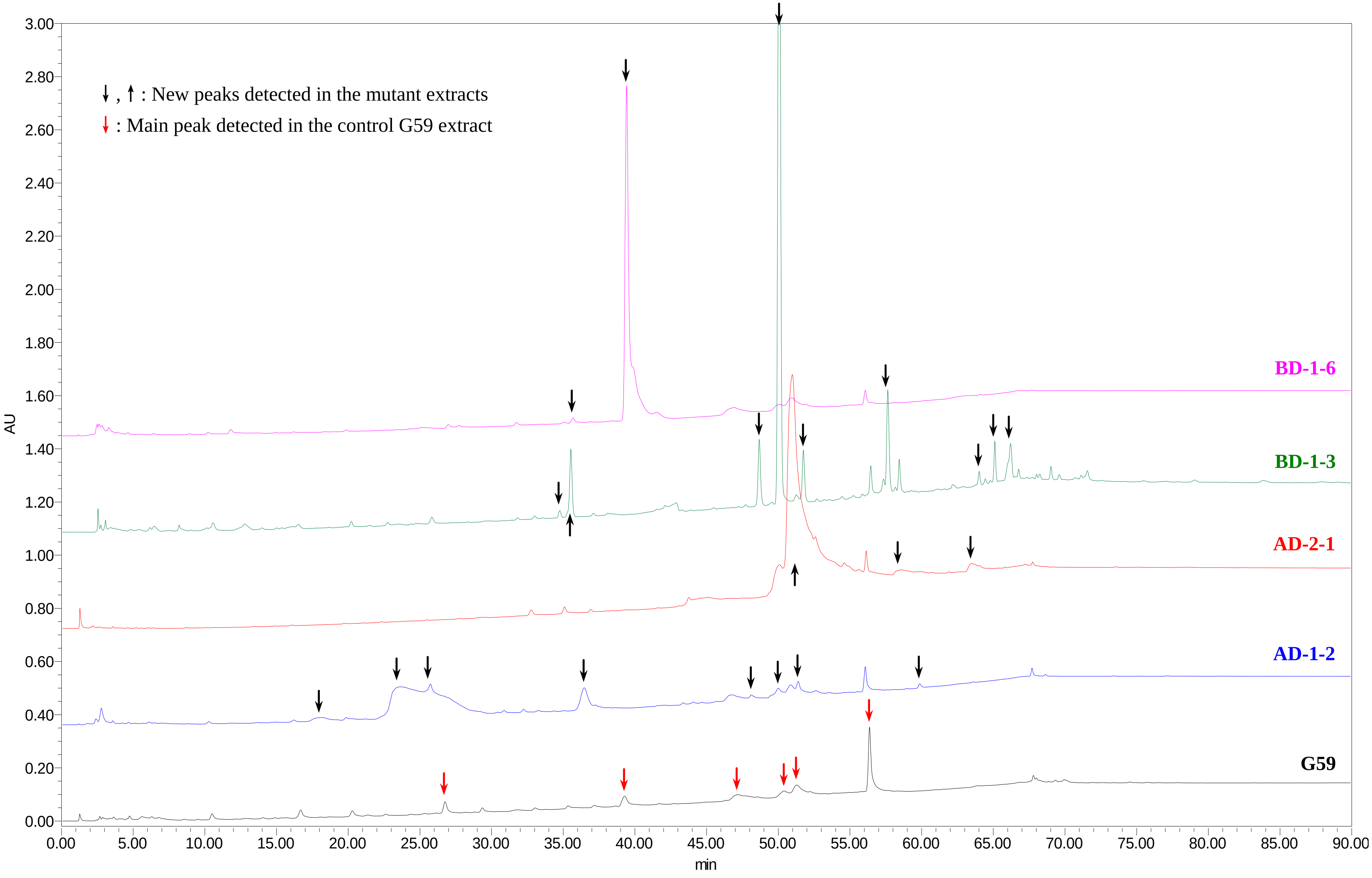

Figure S2 B. HPLC-PDAD-UV analysis of the EtOAc extracts of the control G59 strain and selected mutants. **B**: HPLC profiles detected at 254 nm.

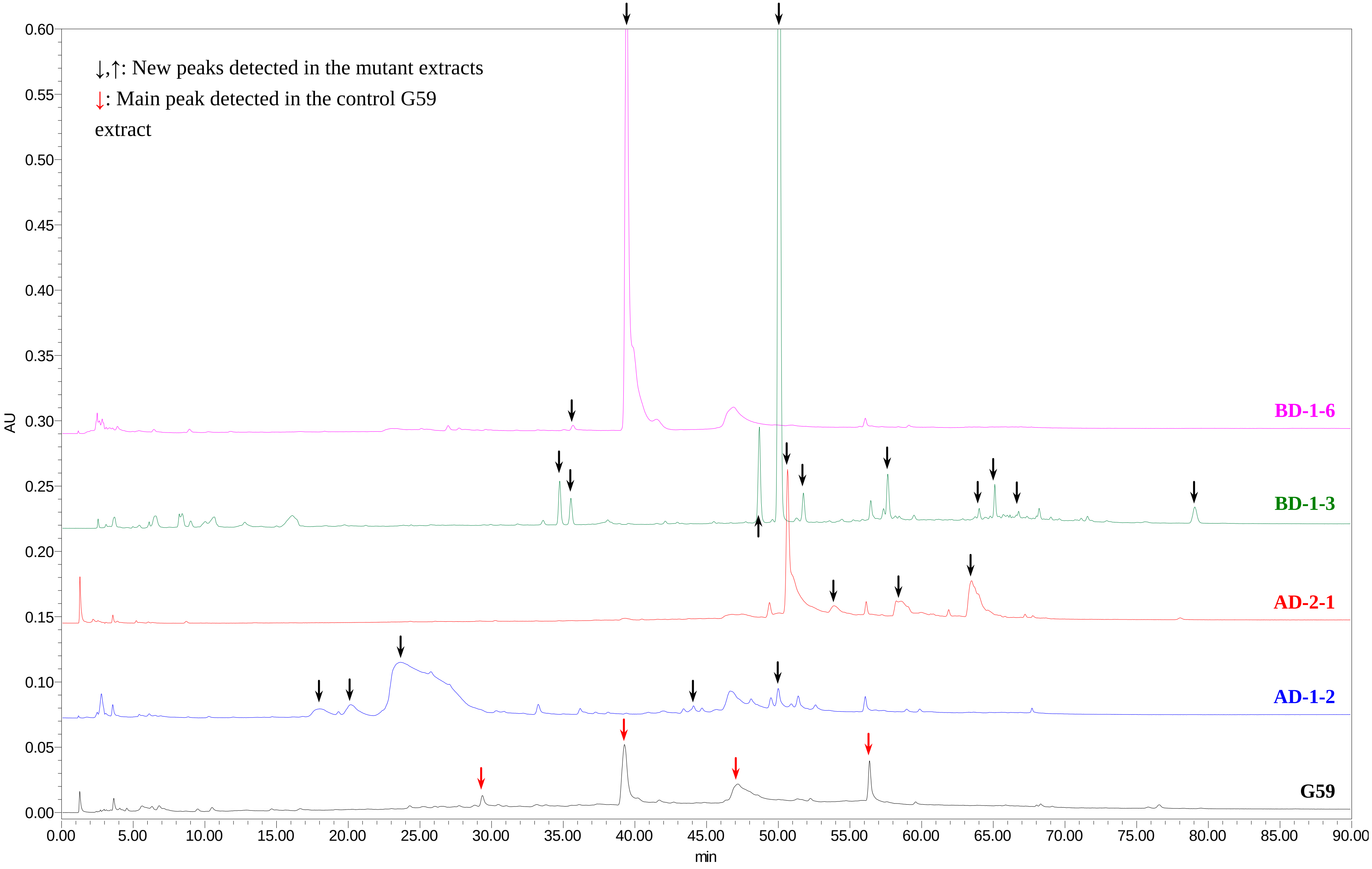

Figure S2 C. HPLC-PDAD-UV analysis of the EtOAc extracts of the control G59 strain and selected mutants. **C**: UV spectra of new peaks in the mutant AD-1-2 and AD-2-1 extracts, and UV absorptions of the control G59 extract at the corresponding retention times.

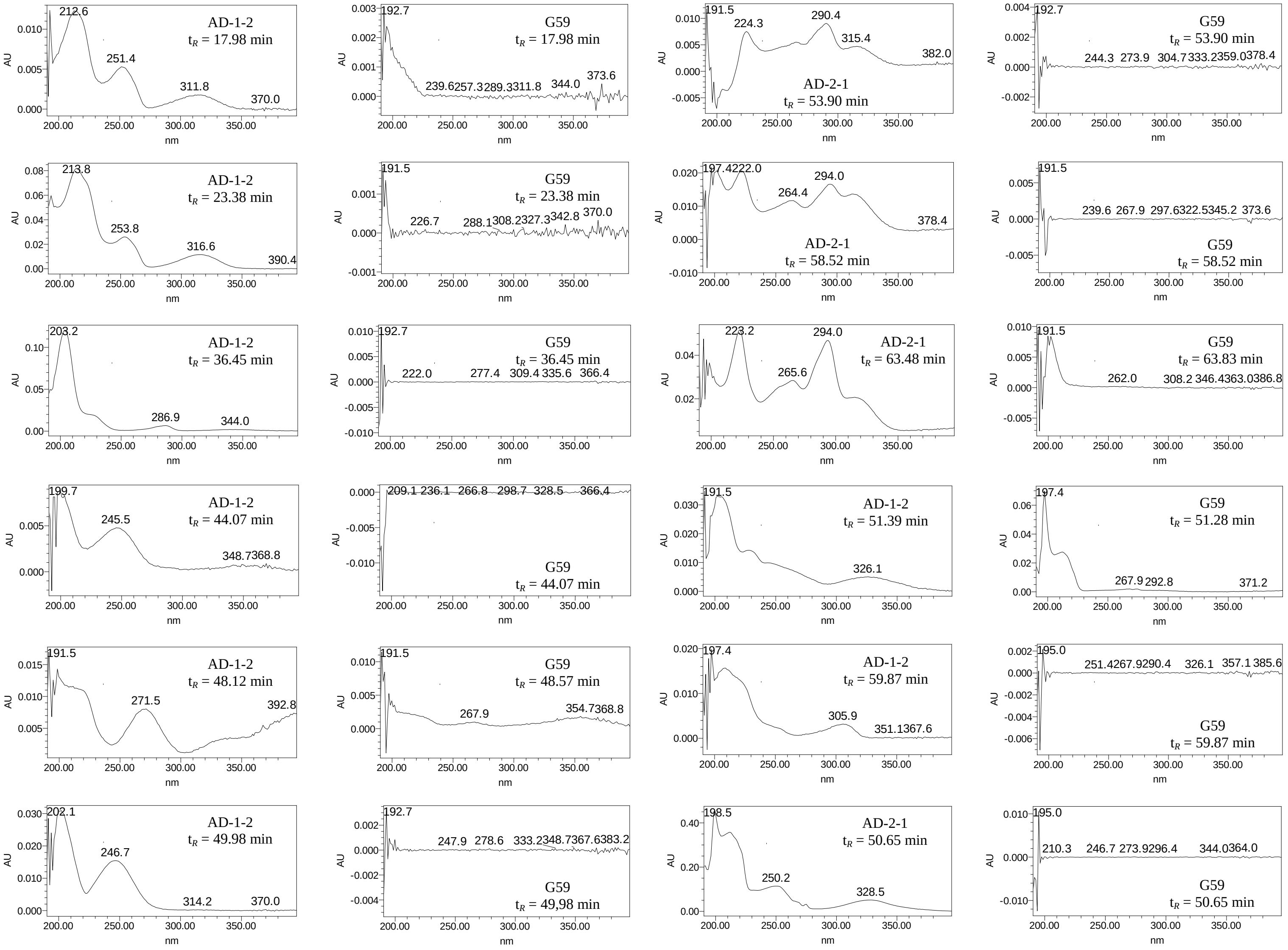

Figure S2 D. HPLC-PDAD-UV analysis of the EtOAc extracts of the control G59 strain and selected mutants. **D**: UV spectra of new peaks in the mutant BD-1-3 and BD-1-6 extracts, and UV absorptions of the control G59 extract at the corresponding retention times.

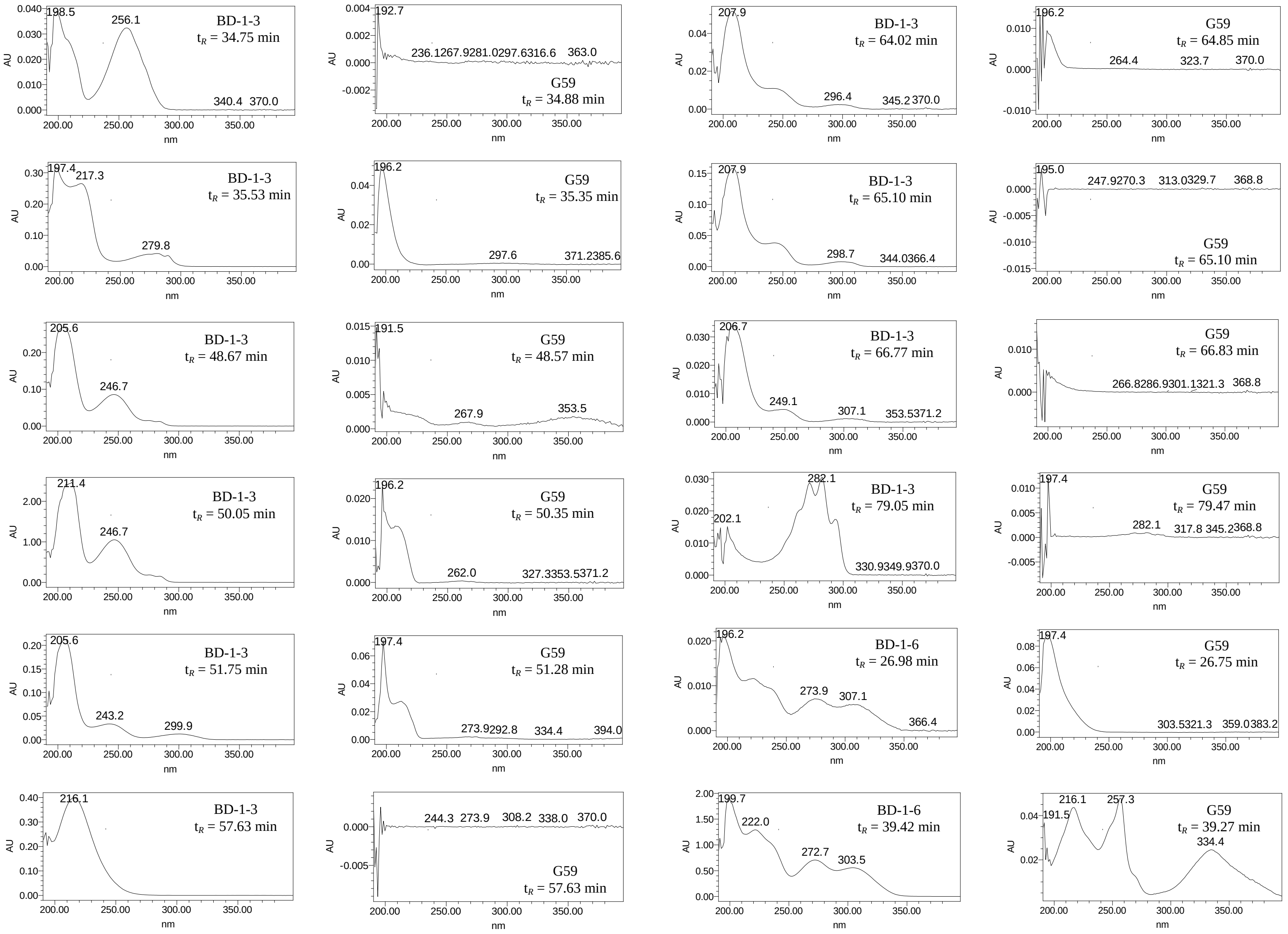

Figure S3 A. HPLC-ESI-MS analysis of the EtOAc extracts of the control G59 strain and selected mutants. **A**: Total ion chromatograms detected by positive ESI-MS.

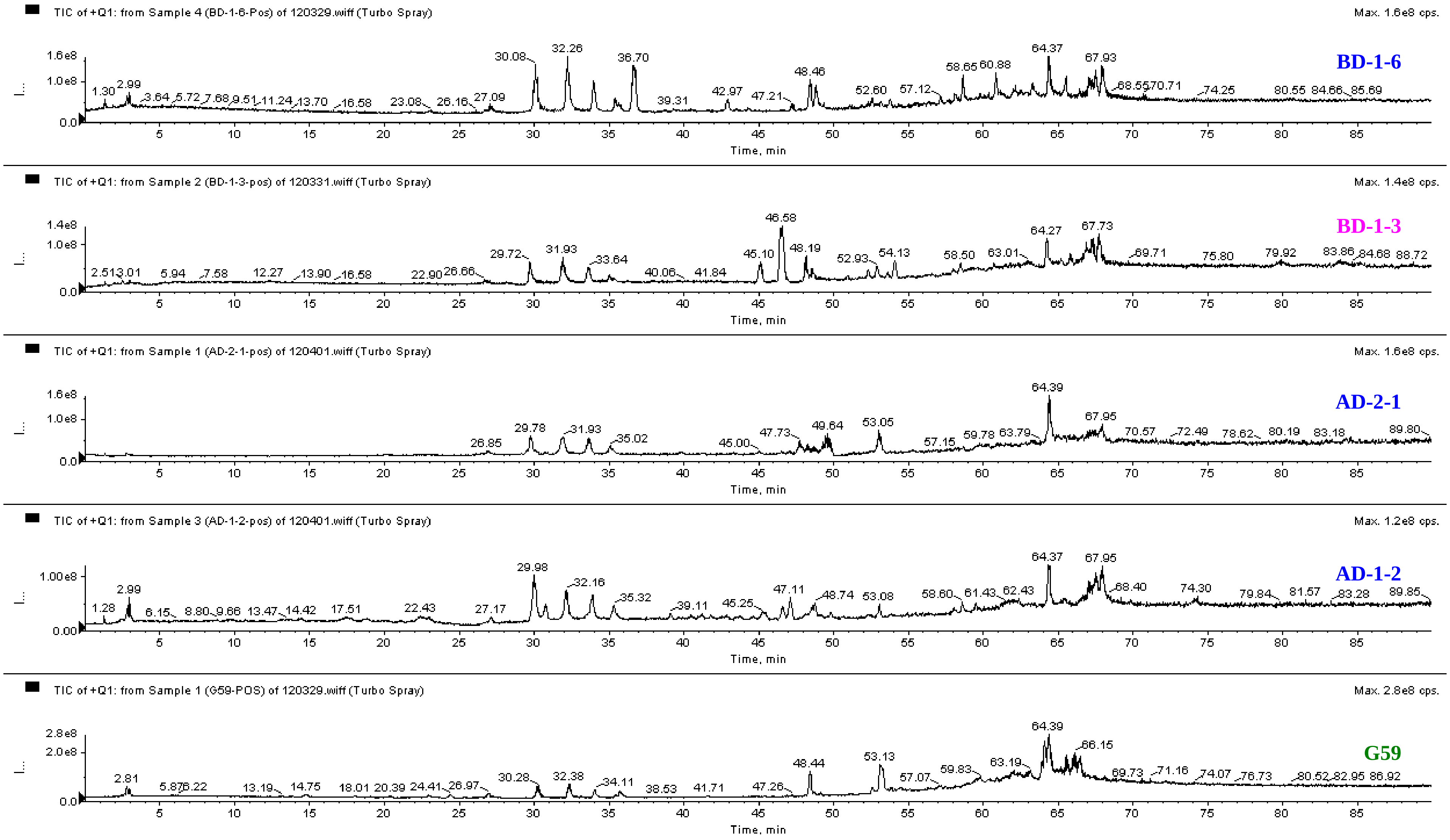

Figure S3 B. HPLC-ESI-MS analysis of the EtOAc extracts of the control G59 strain and selected mutants. **B**: Total ion chromatograms detected by negative ESI-MS.

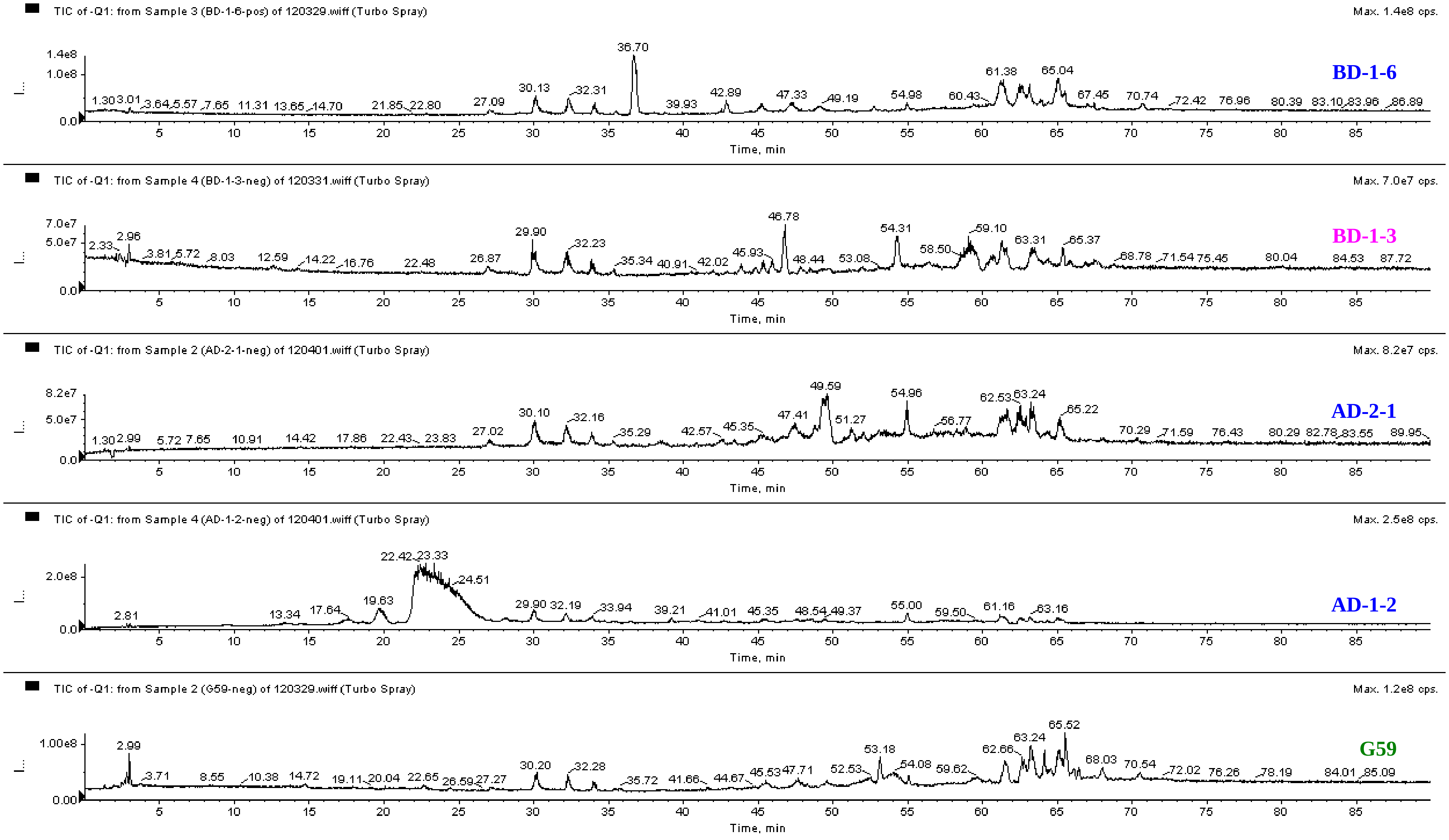

Figure S3 C. HPLC-ESI-MS analysis of the EtOAc extracts of the control G59 strain and selected mutants. **C**: Positive and negative ESI-MS spectra at the given retention times in total ion chromatograms (TICs) of the control G59 and the mutants BD-1-6 and BD-1-3.

ESIMS spectra in the range of 36.5–37.0 min retention times in TICs of G59 and BD-1-6

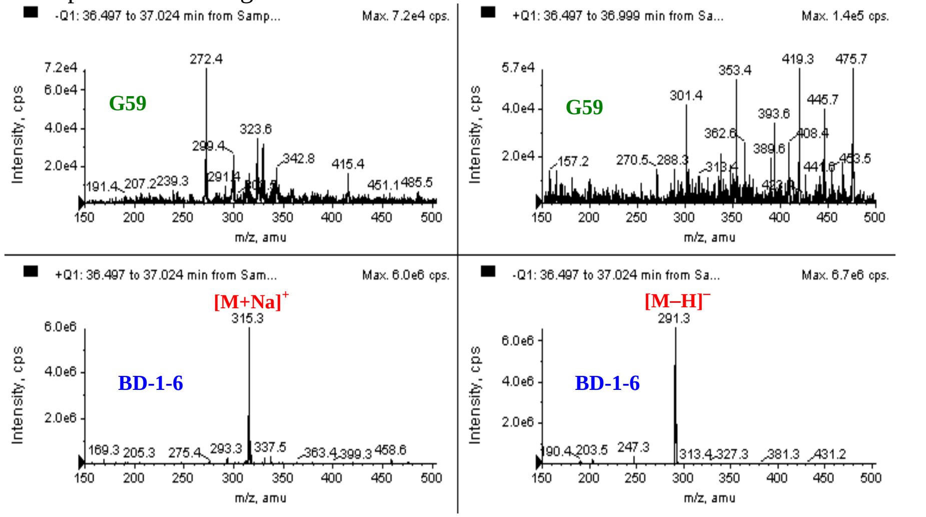

ESIMS spectra in the range of 42.7–43.1 min retention times in TICs of G59 and BD-1-6

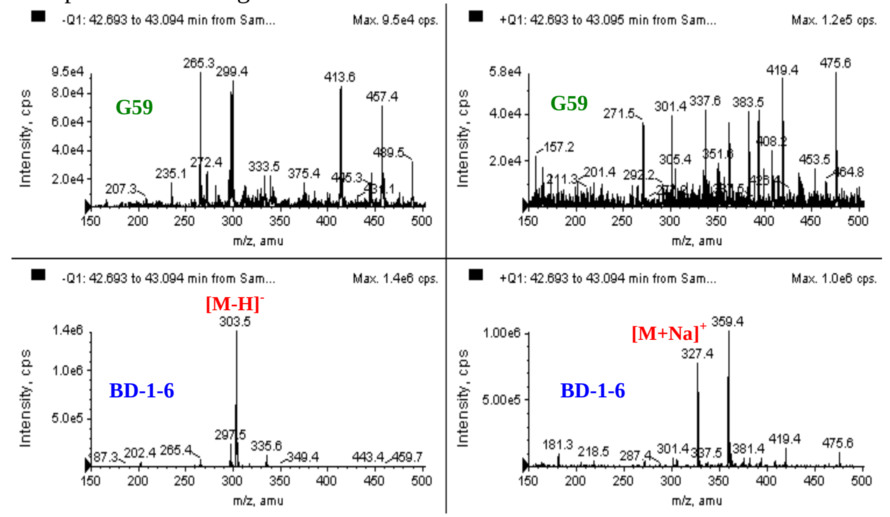

ESIMS spectra in the range of 44.8–45.4 min retention times in TICs of G59 and BD-1-3

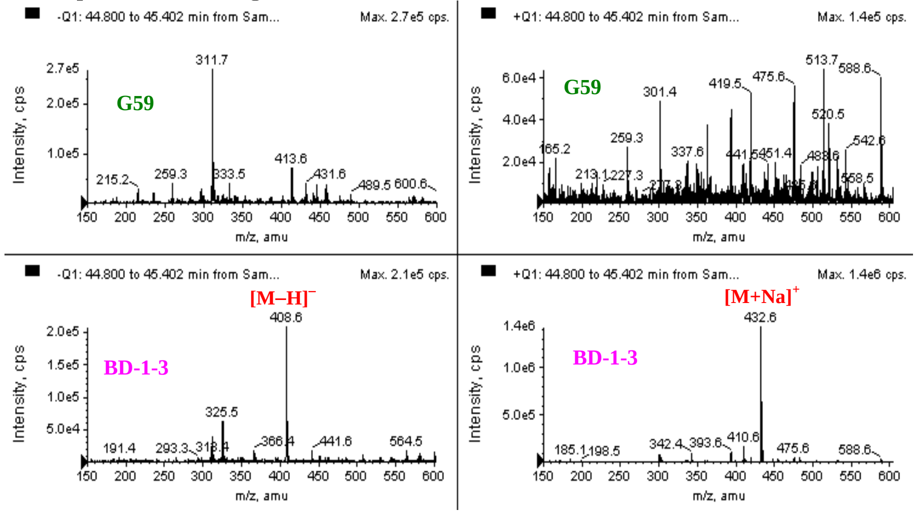

ESIMS spectra in the range of 46.5–47.0 min retention times in TICs of G59 and BD-1-3

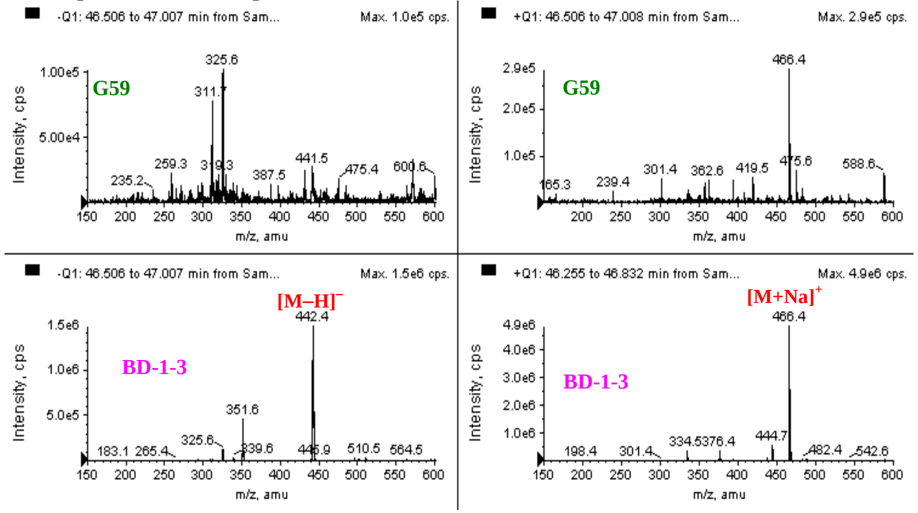

Figure S3 D. HPLC-ESI-MS analysis of the EtOAc extracts of the control G59 strain and selected mutants. **D**: Positive and negative ESI-MS spectra at the given retention times in total ion chromatograms (TICs) of the control G59 and the mutants AD-2-1 and Ad-1-2.

ESIMS spectra in the range of 49.0–50.0 min retention times in TICs of G59 and AD-2-1

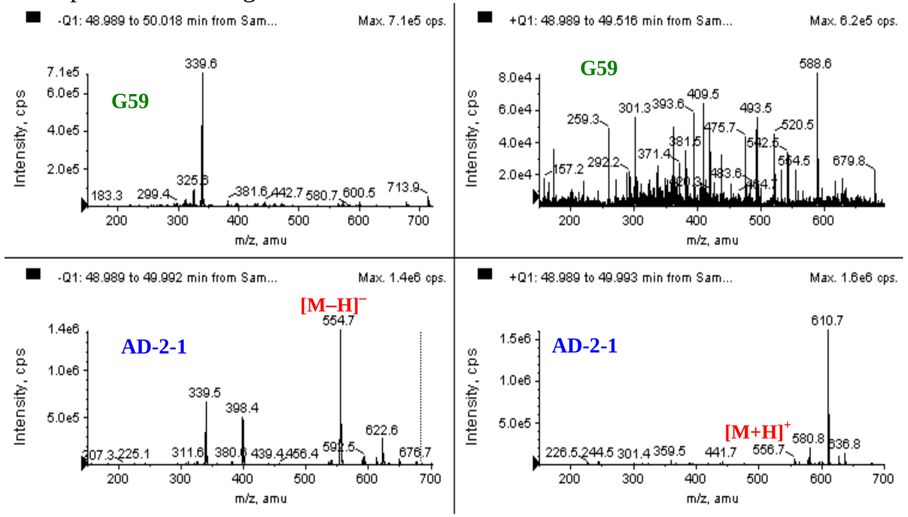

ESIMS spectra in the range of 64.0–65.0 min retention times in TICs of G59 and AD-2-1

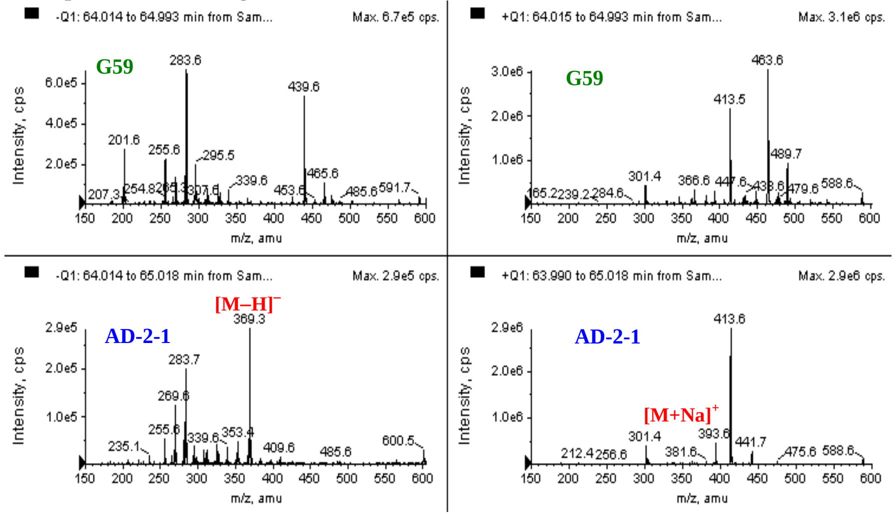

ESIMS spectra in the range of 21.5–26.5 min retention times in TICs of G59 and AD-1-2

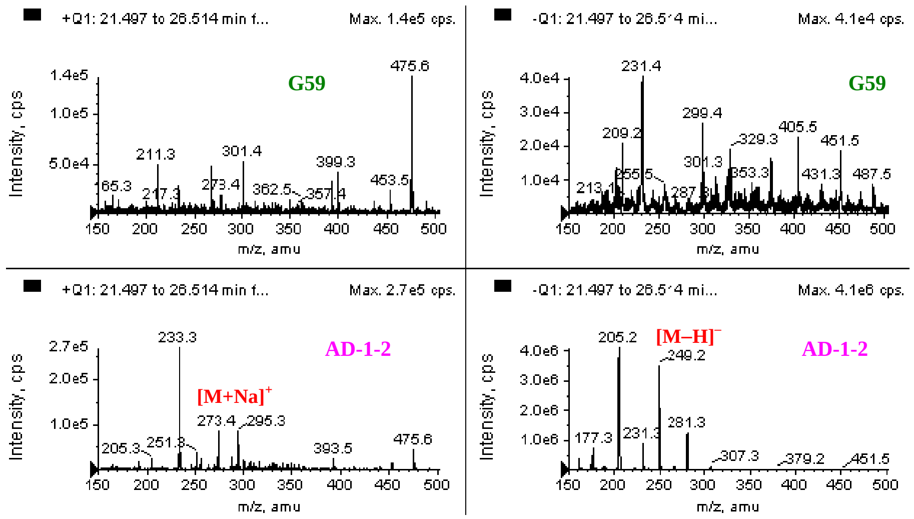

ESIMS spectra in the range of 48.4–49.0 min retention times in TICs of G59 and AD-1-2

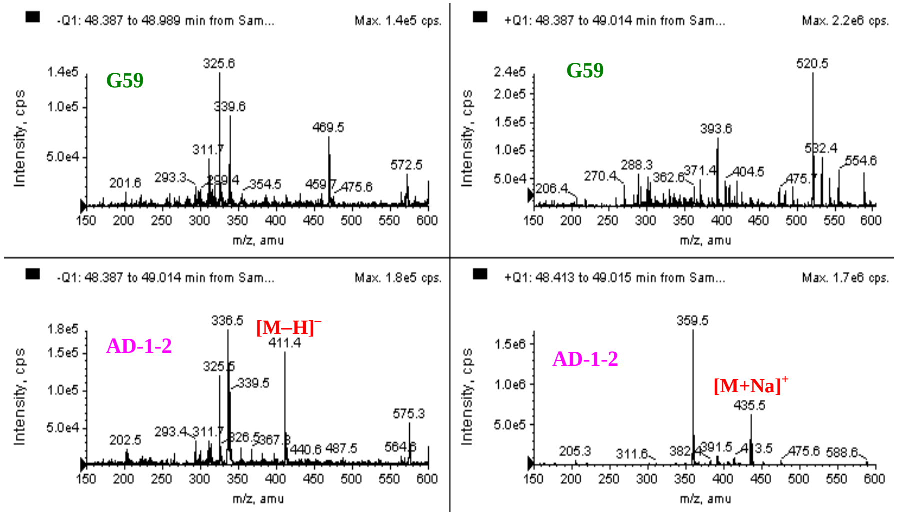

Figure S4. HPLC-PDAD-UV analysis of the EtOAc extracts of the strain G59 and the mutant BD-1-3 for detecting **1**–**5**.

HPLC profiles of **1**–**5** and the EtOAc extracts of the mutant BD-1-3 and the control G59 detected at 210 nm

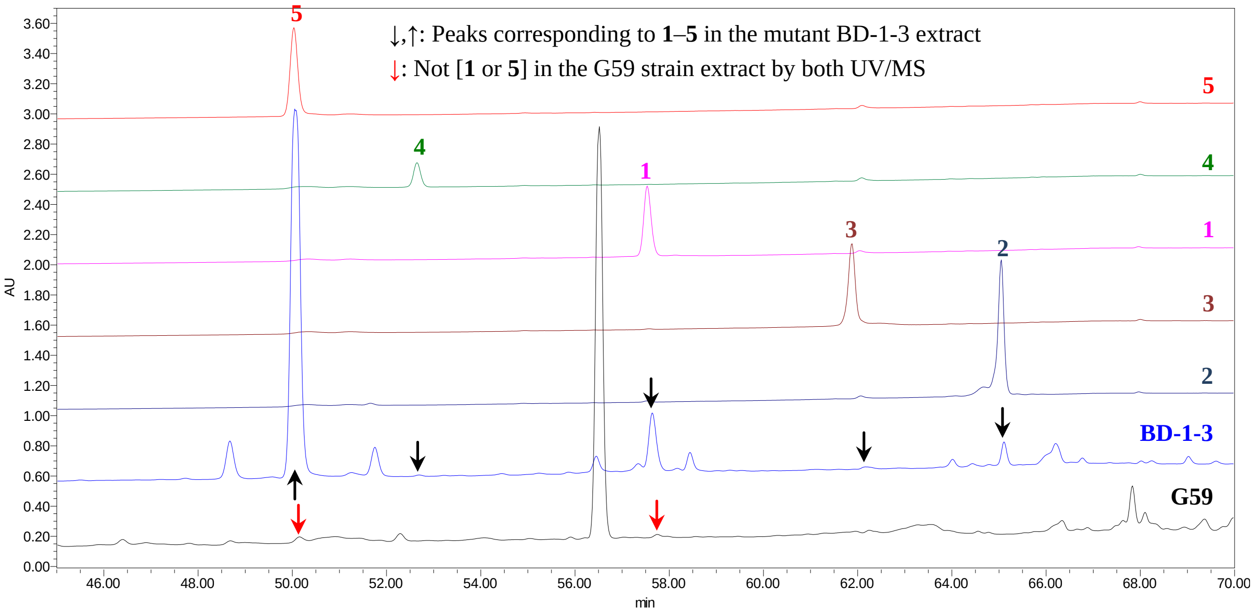

UV spectra of **1**–**5** and the corresponding peaks in the mutant BD-1-3 and control G59 extracts

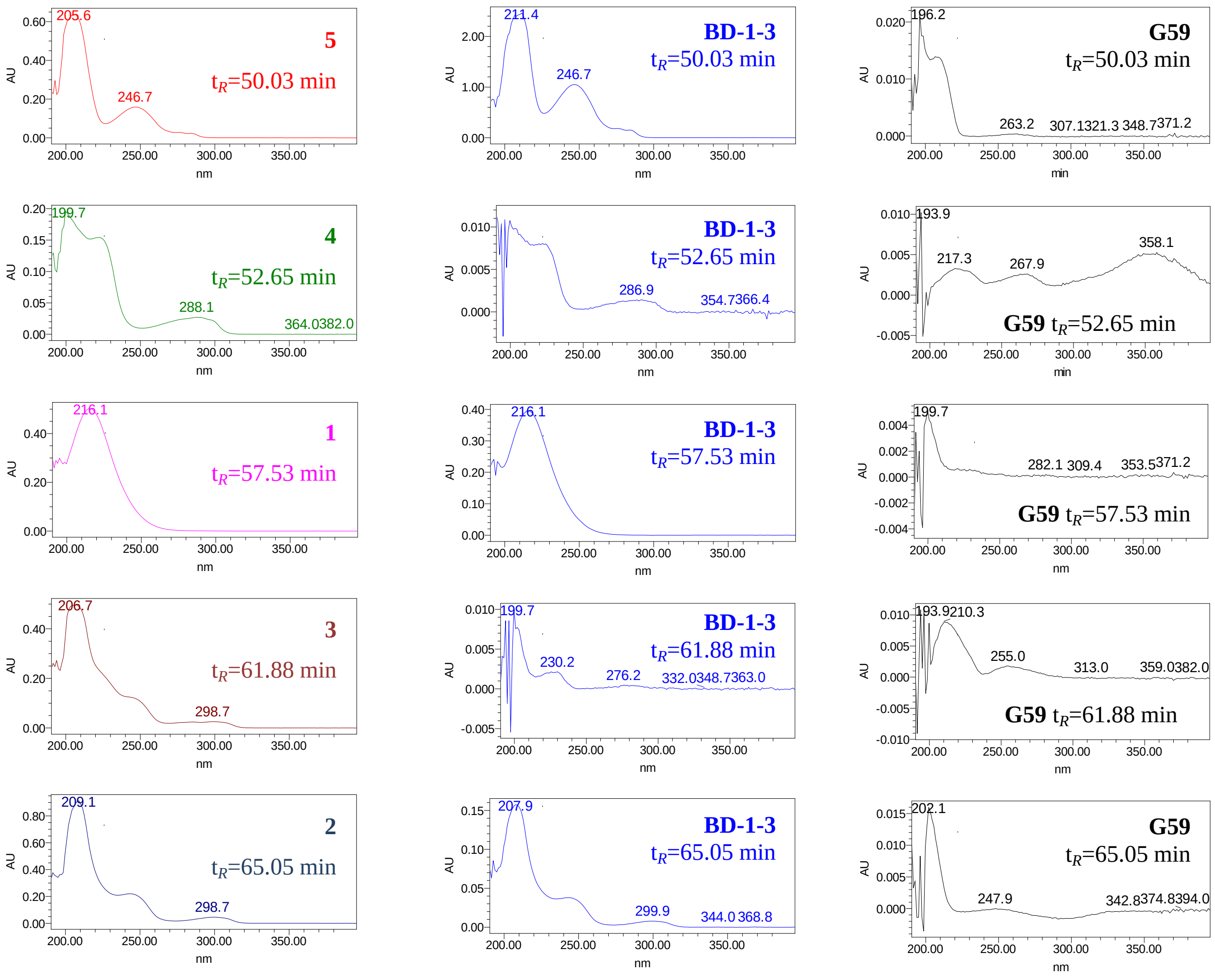

Figure S5 A. HPLC-ESI-MS analysis of the EtOAc extracts of the strain G59 and the mutant BD-1-3 for detecting 1–5. **A**: HPLC-Positive ion ESI-MS analysis (ESIMS *m/z*: 502 $[M+Na]^+$ for **1**).

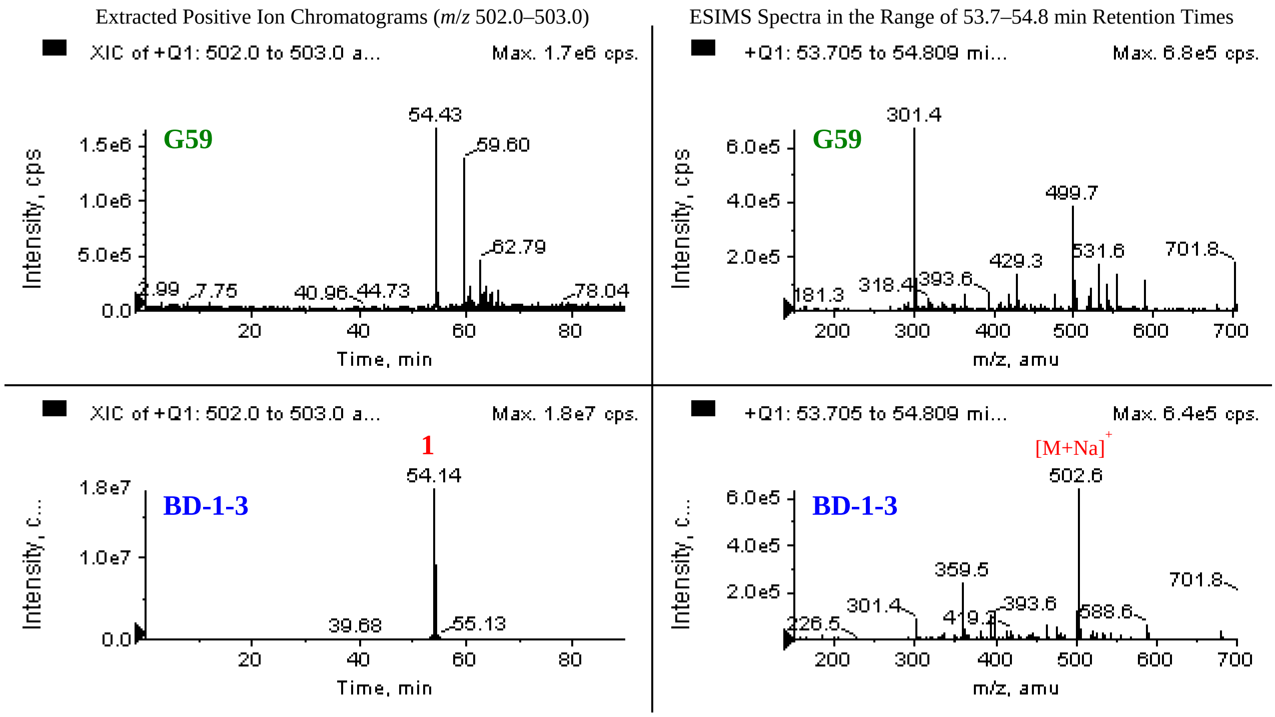

Figure S5 B. HPLC-ESI-MS analysis of the EtOAc extracts of the strain G59 and the mutant BD-1-3 for detecting **1**–**5**. **B**: HPLC-Positive ion ESI-MS analysis (ESIMS *m/z*: 885 $[M+Na]^+$ for **2**).

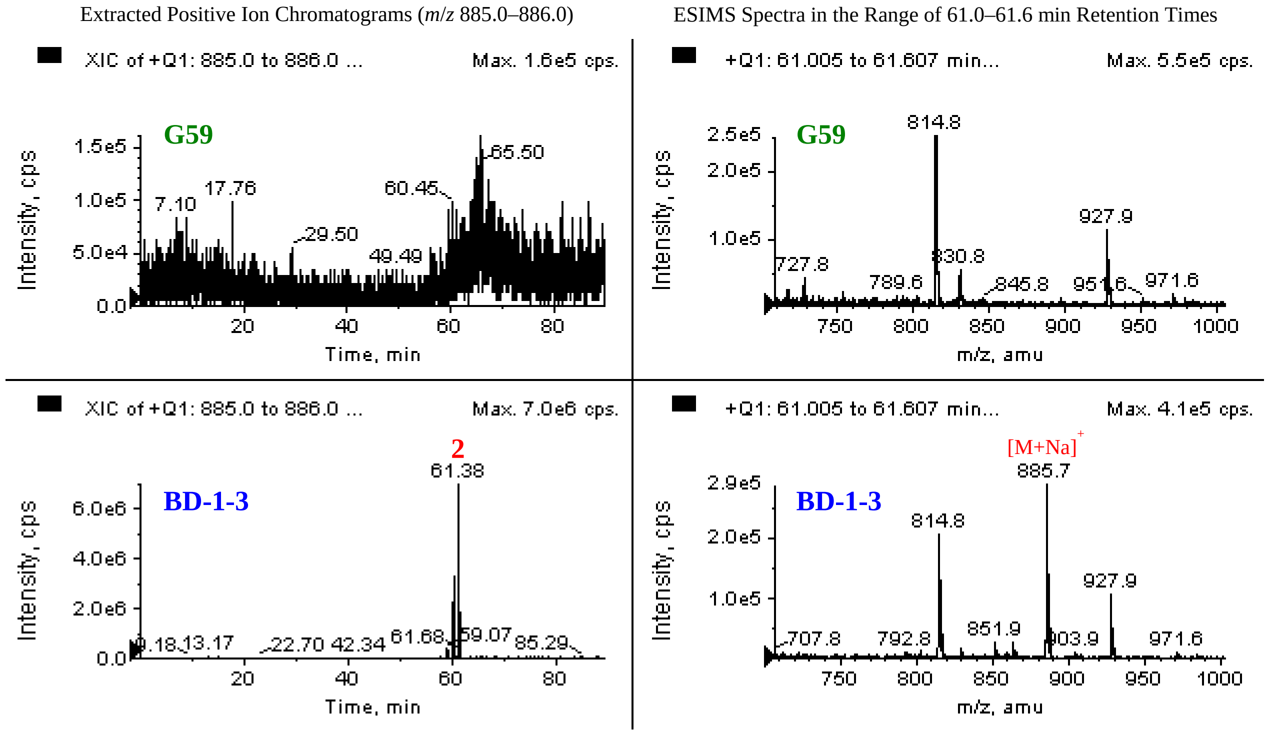

Figure S5 C. HPLC-ESI-MS analysis of the EtOAc extracts of the strain G59 and the mutant BD-1-3 for detecting **1**–**5**. **C**: HPLC-Positive ion ESI-MS analysis (ESIMS *m/z*: 901 [M+Na]$^+$ for **3**).

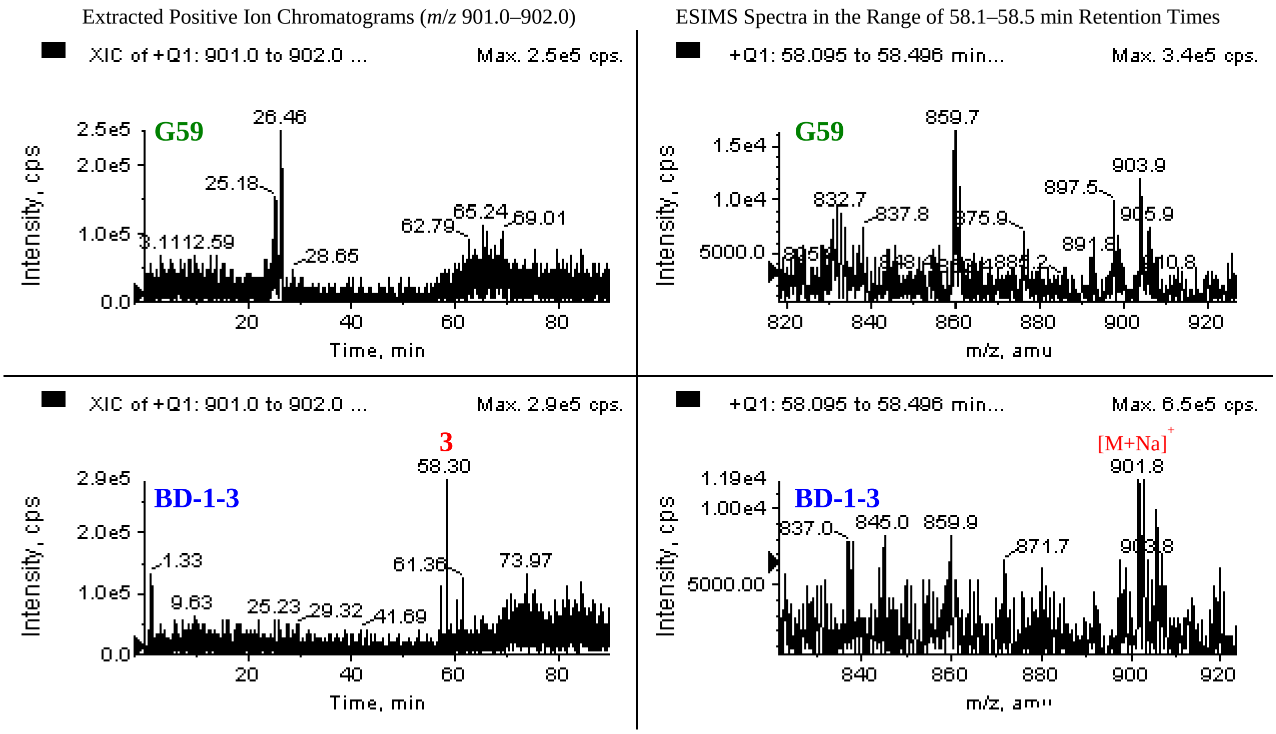

Figure S5 D. HPLC-ESI-MS analysis of the EtOAc extracts of the strain G59 and the mutant BD-1-3 for detecting **1**–**5**. **D**: HPLC-Negative ion ESI-MS analysis (ESIMS *m/z*: 400 [M-H]$^-$ for **4**).

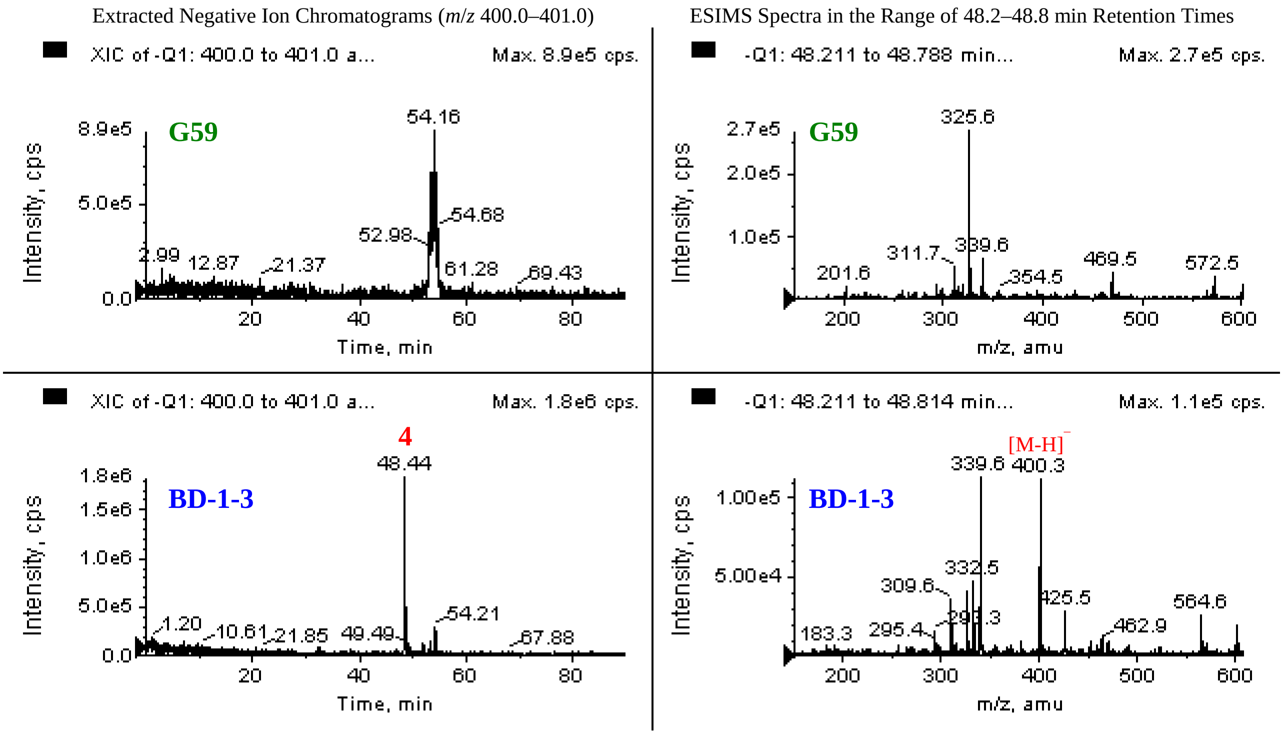

Figure S5 E. HPLC-ESI-MS analysis of the EtOAc extracts of the strain G59 and the mutant BD-1-3 for detecting **1**–**5**. **E**: HPLC-Negative ion ESI-MS analysis (ESIMS *m*/*z*: 442 [M-H]$^-$ for **5**).

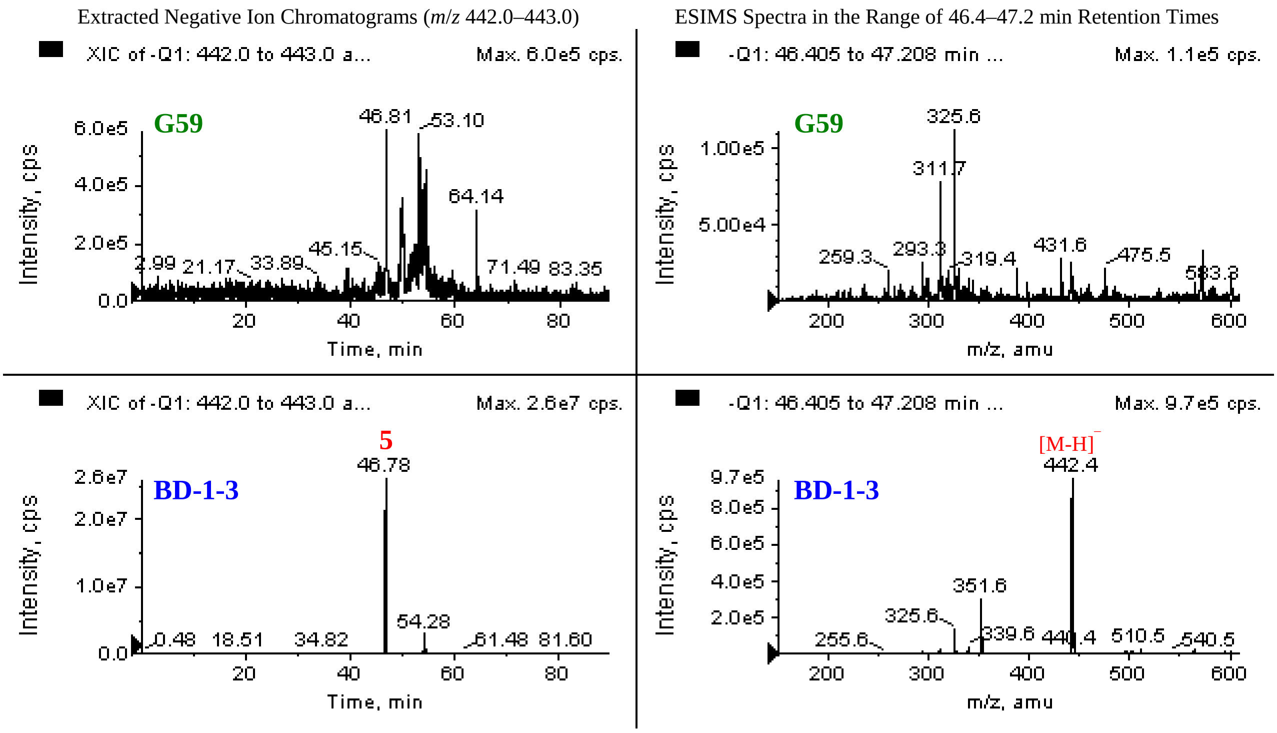

Appendix of Spectra

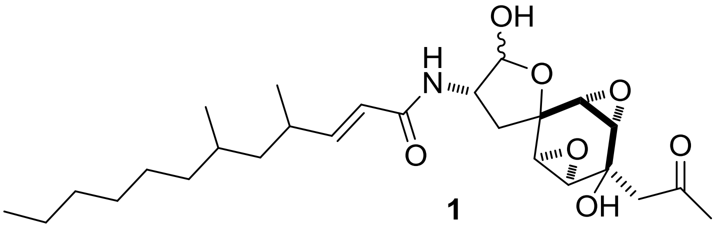

1

Figure SP1. A: Positive (A) and negative (B) ESIMS spectra of **1**.

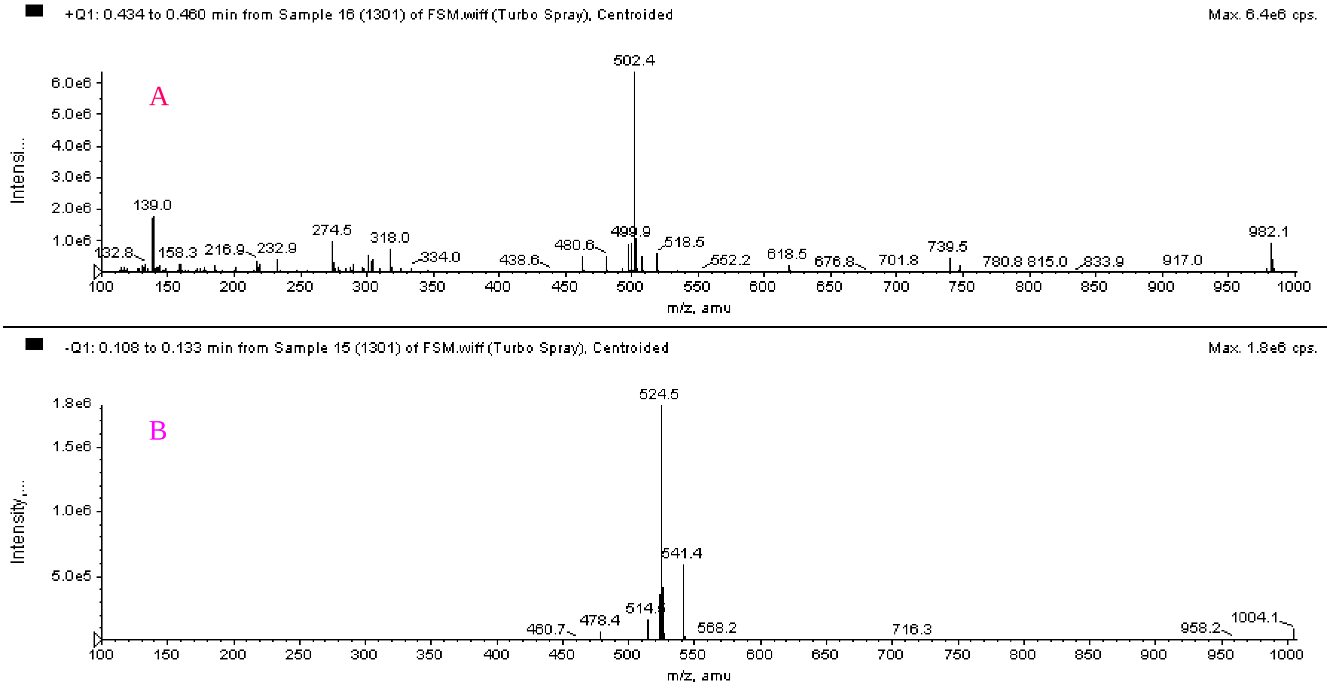

Figure SP1. B: Positive HRESIMS spectrum of **1**.

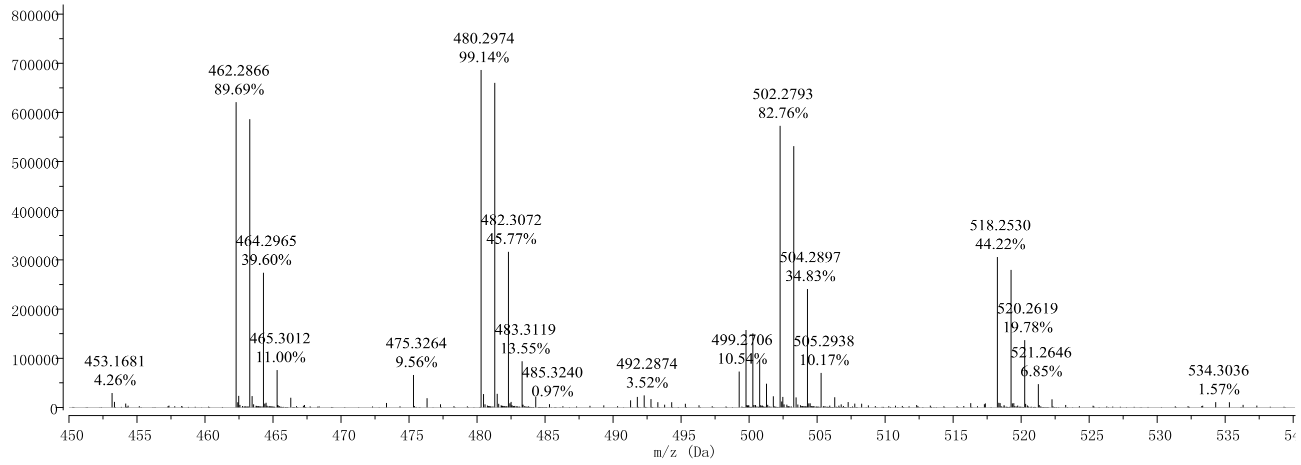

Figure SP1. C: IR spectrum of **1**.

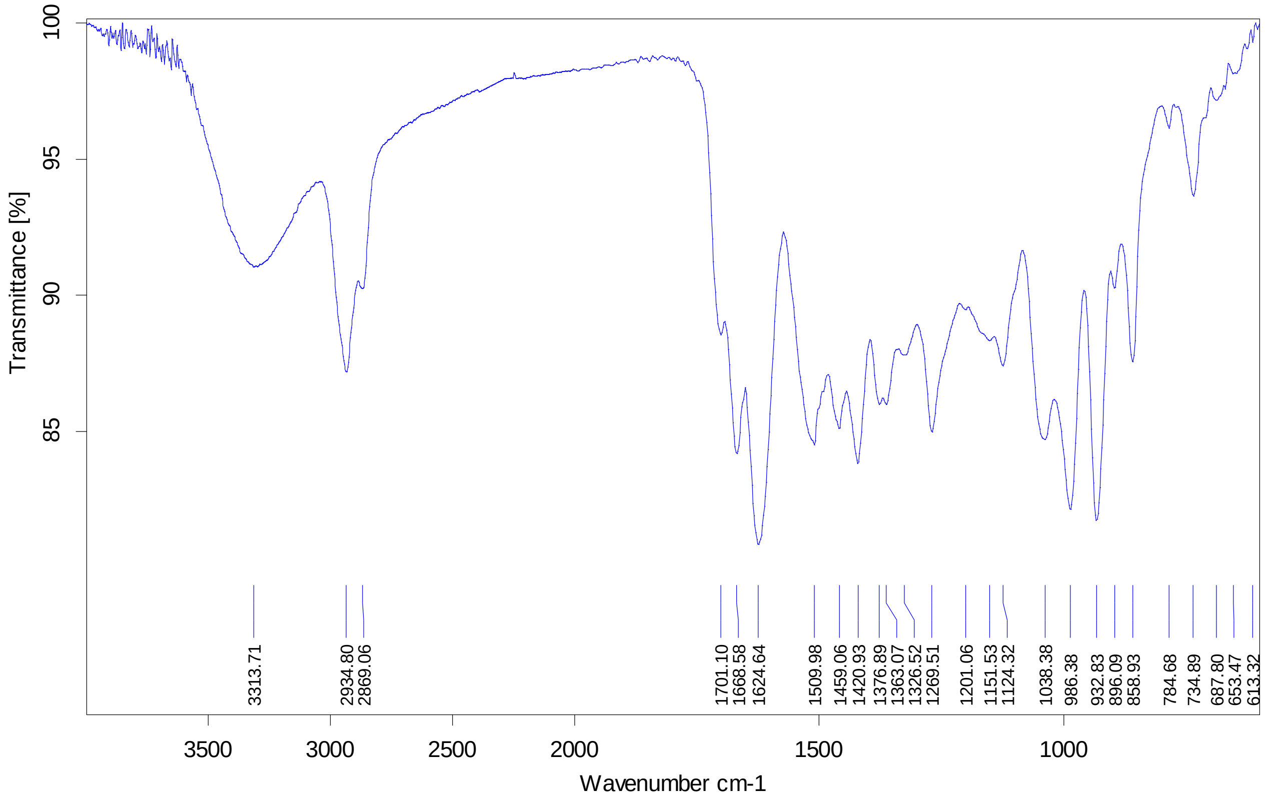

Figure SP1. D: UV spectrum of **1** in MeOH.

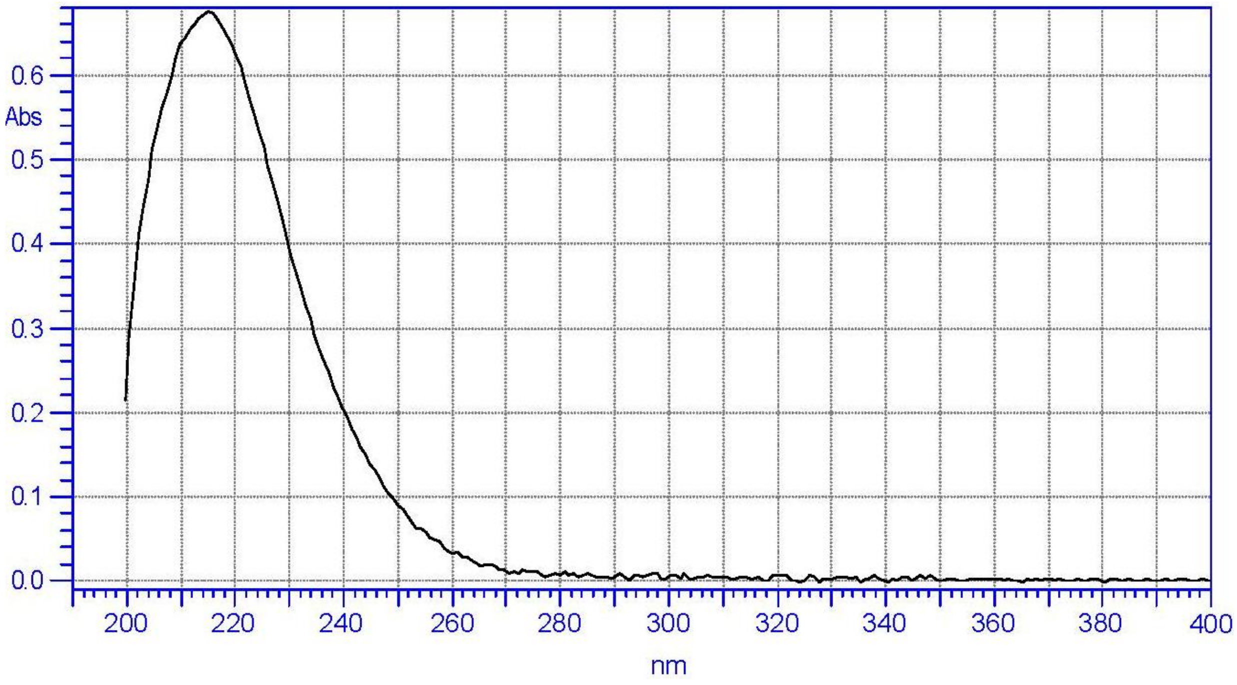

Figure SP1. E: 600 MHz ^{1}H-NMR spectrum of **1** (measured soon after dissolving **1** in $CDCl_3$).

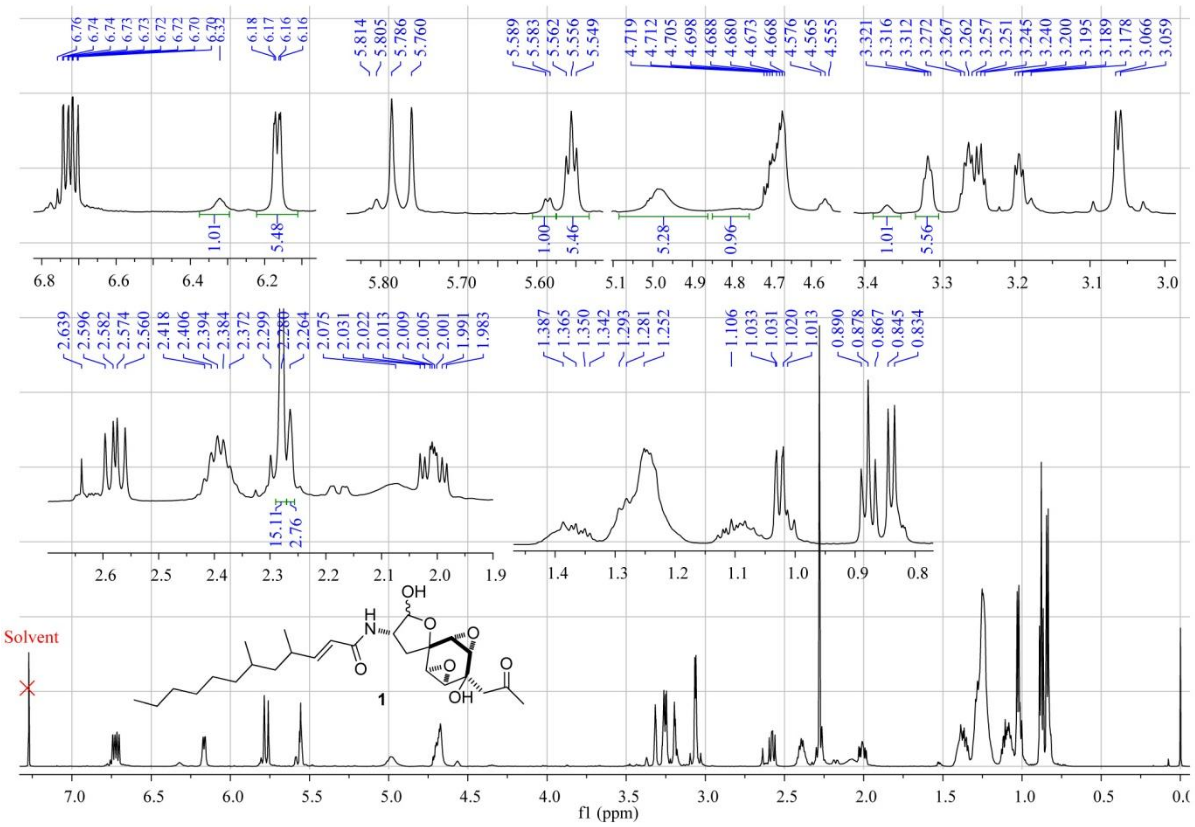

Figure SP1. F: 600 MHz ^{1}H-NMR spectrum of **1** (measured after over a week in the $CDCl_3$ solution).

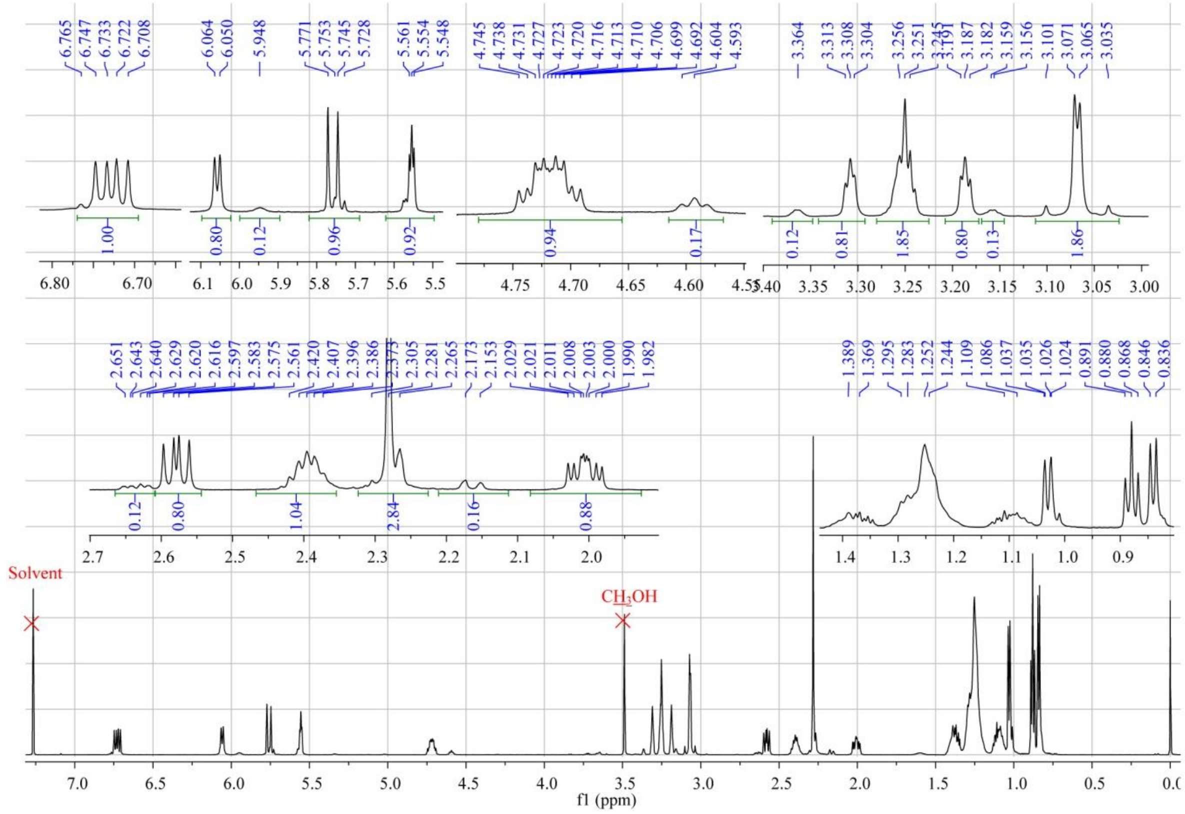

Figure SP1. G: 150 MHz ^{13}C-NMR spectrum of **1** in $CDCl_3$.

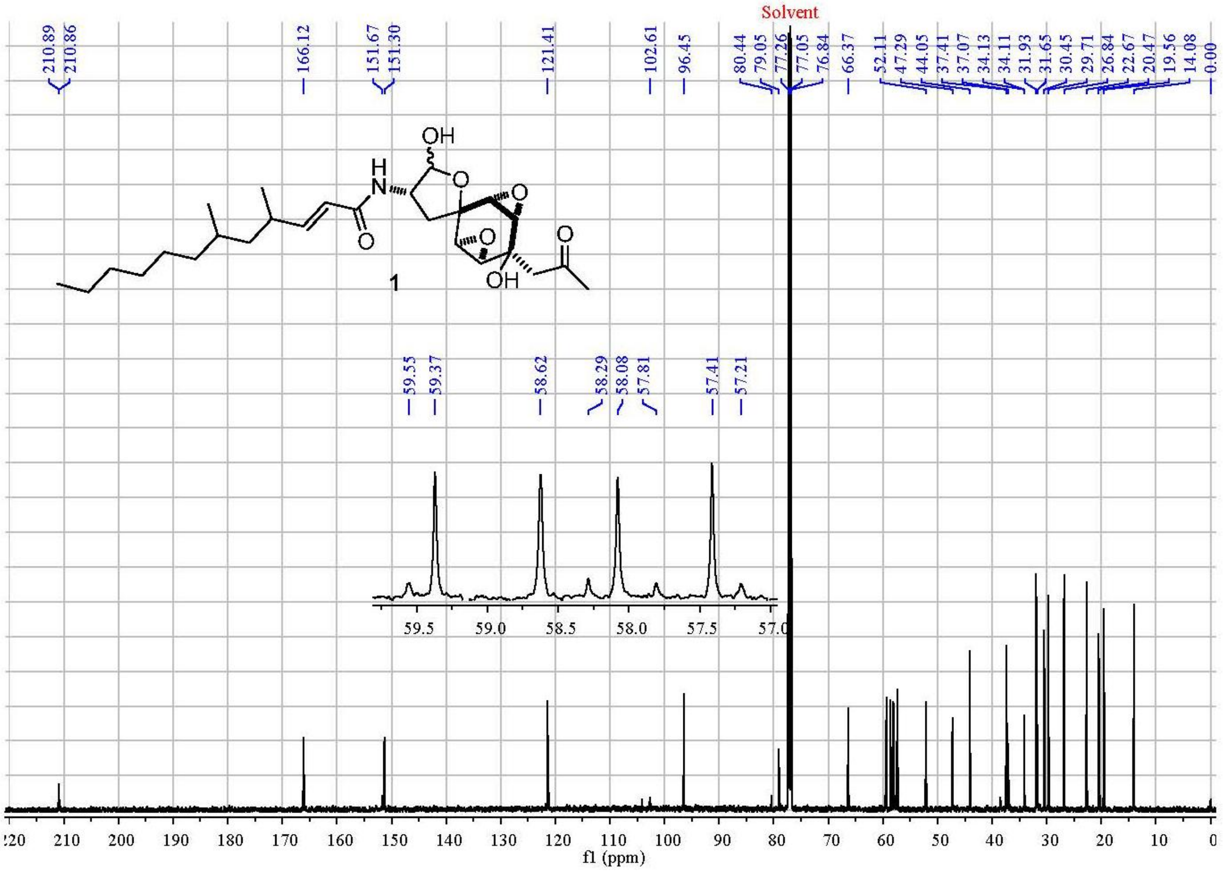

Figure SP1. H: DEPT spectra of **1** in $CDCl_3$.

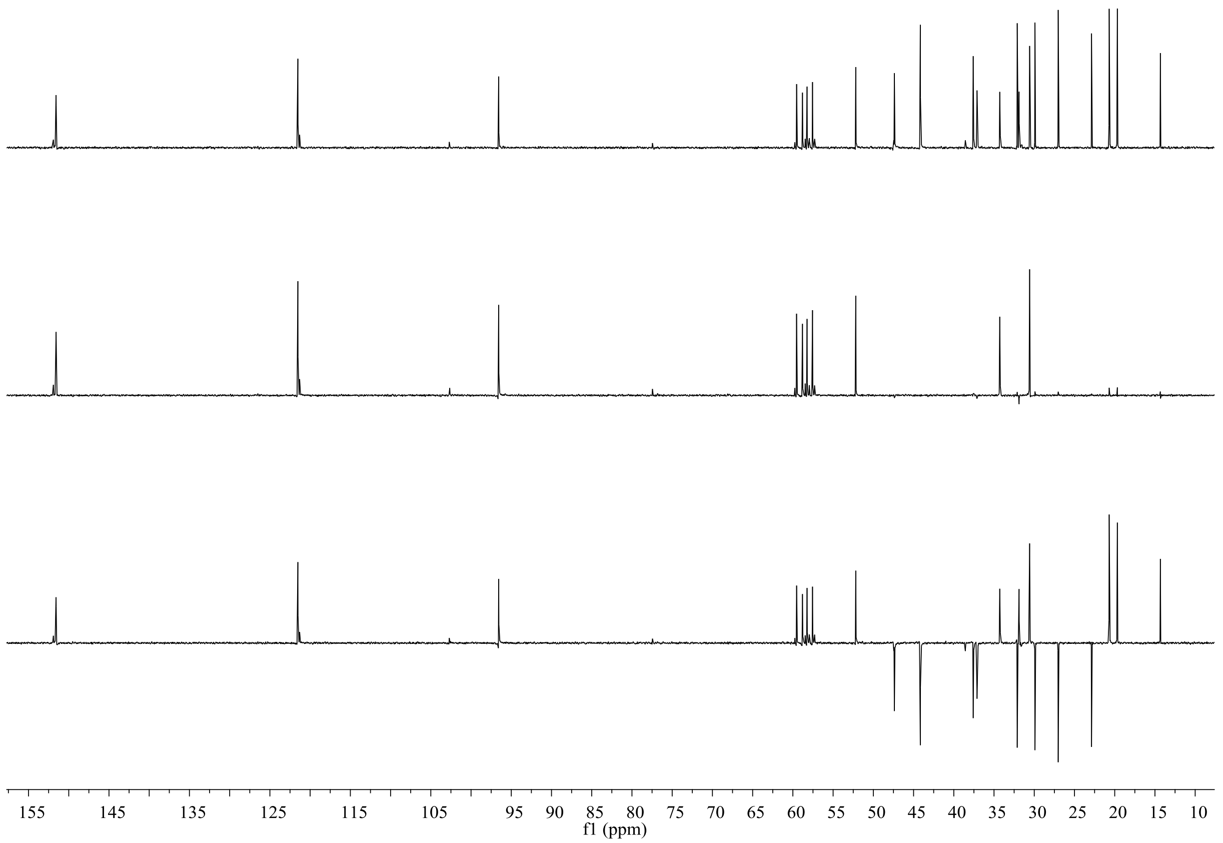

Figure SP1. I: ^{1}H-^{1}H COSY spectrum of **1** in $CDCl_3$.

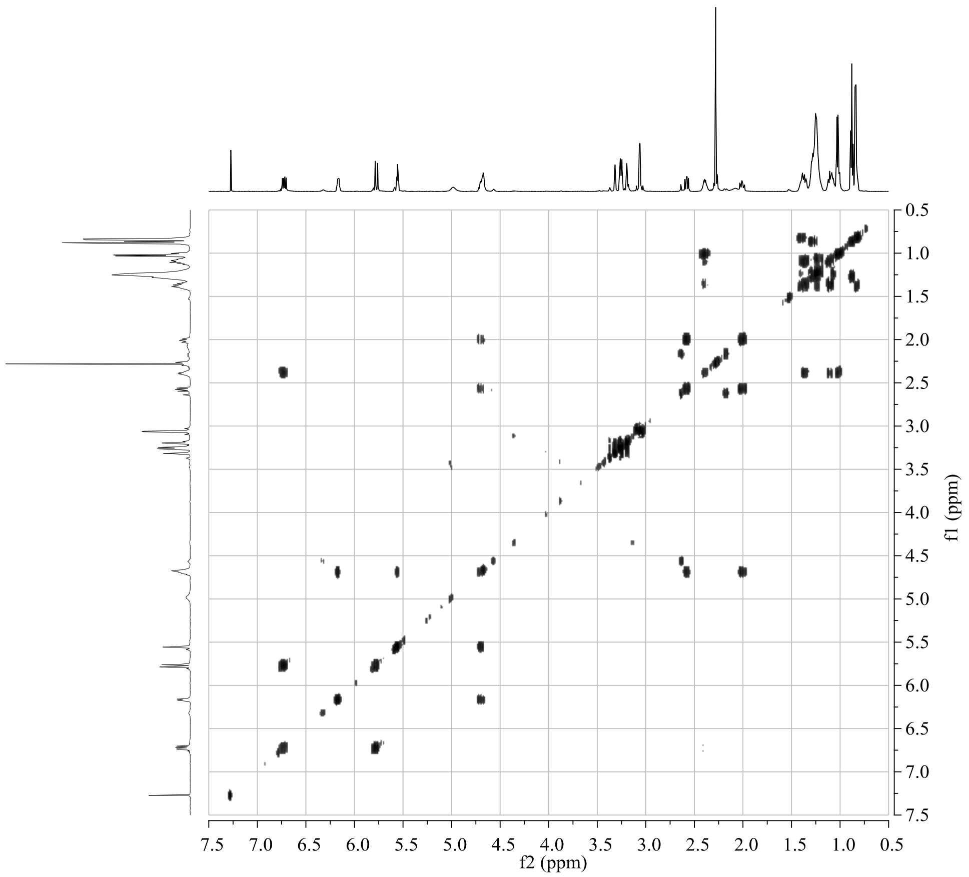

Figure SP1. J: HMQC spectrum of **1** in $CDCl_3$.

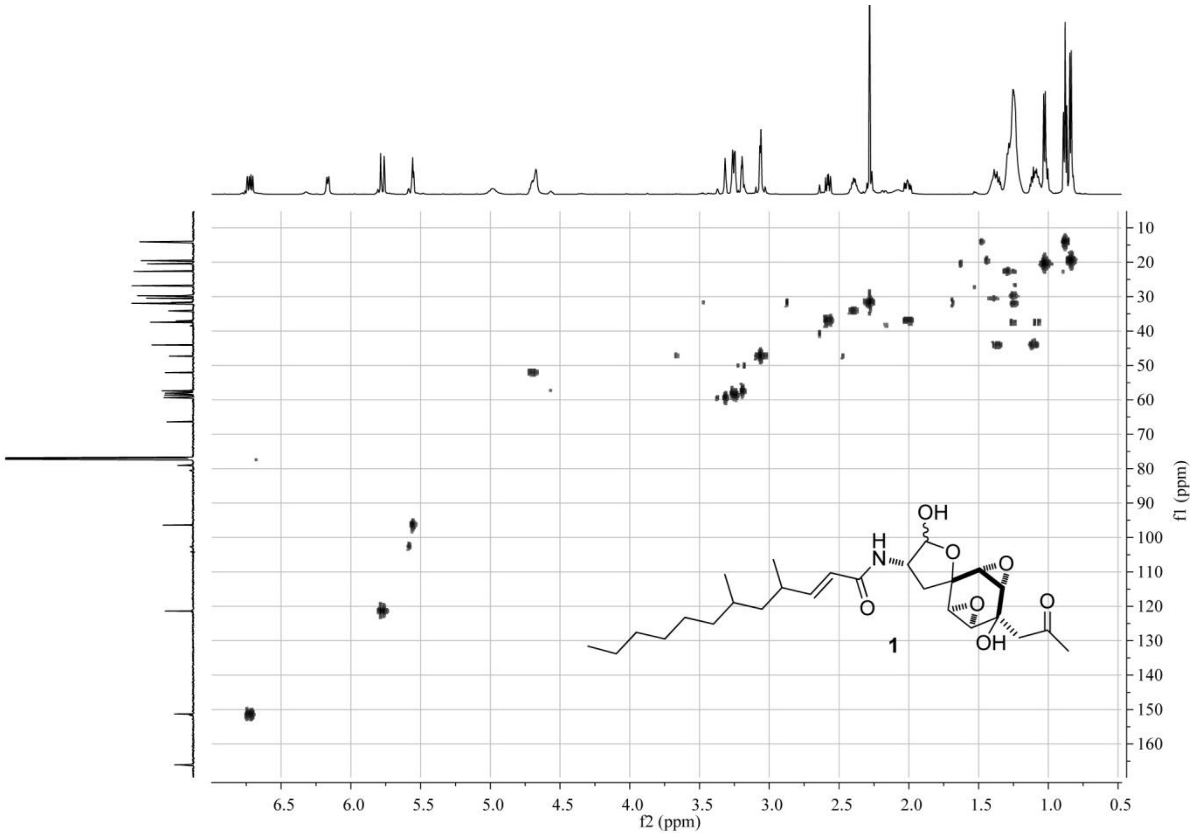

Figure SP1. K: HMBC spectrum of **1** in $CDCl_3$.

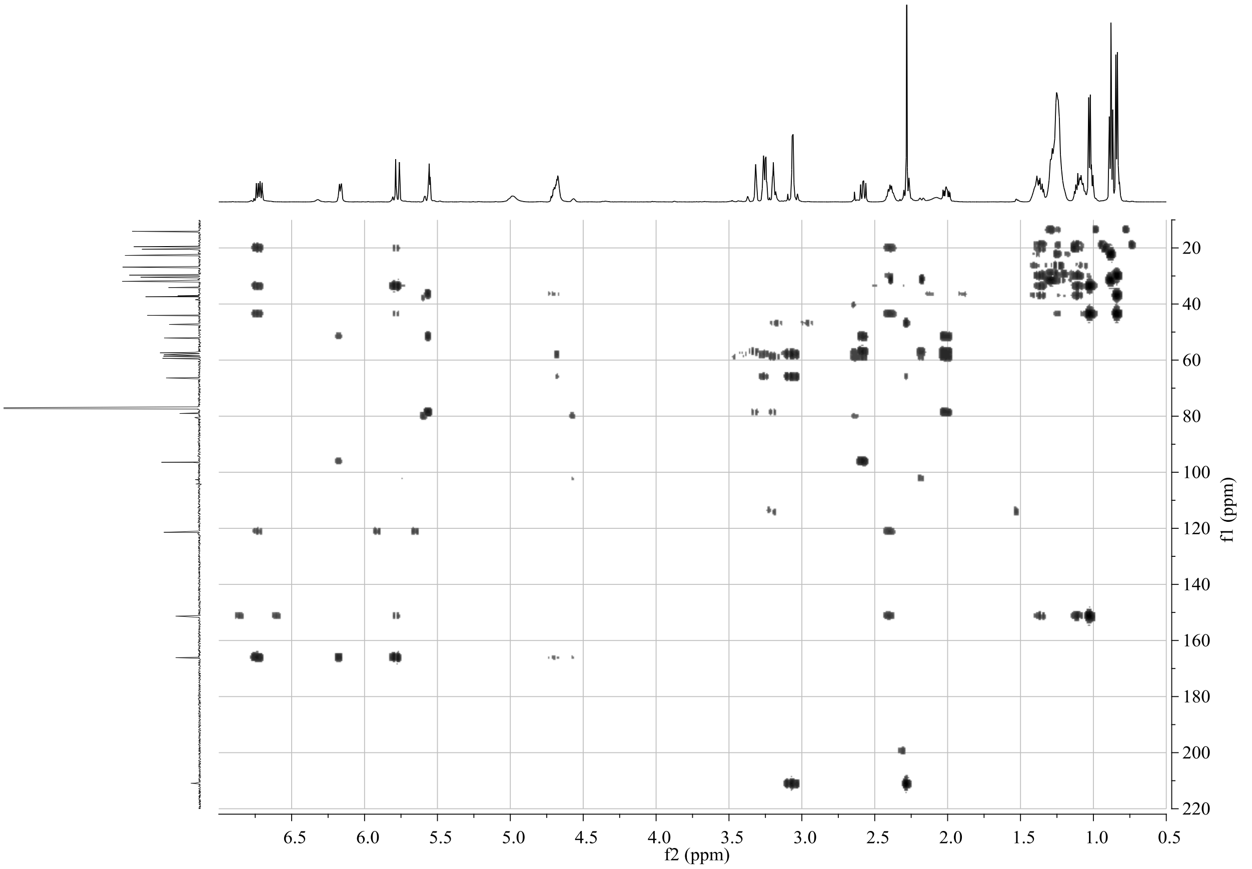

Figure SP1. L: NOESY spectrum of **1** in $CDCl_3$.

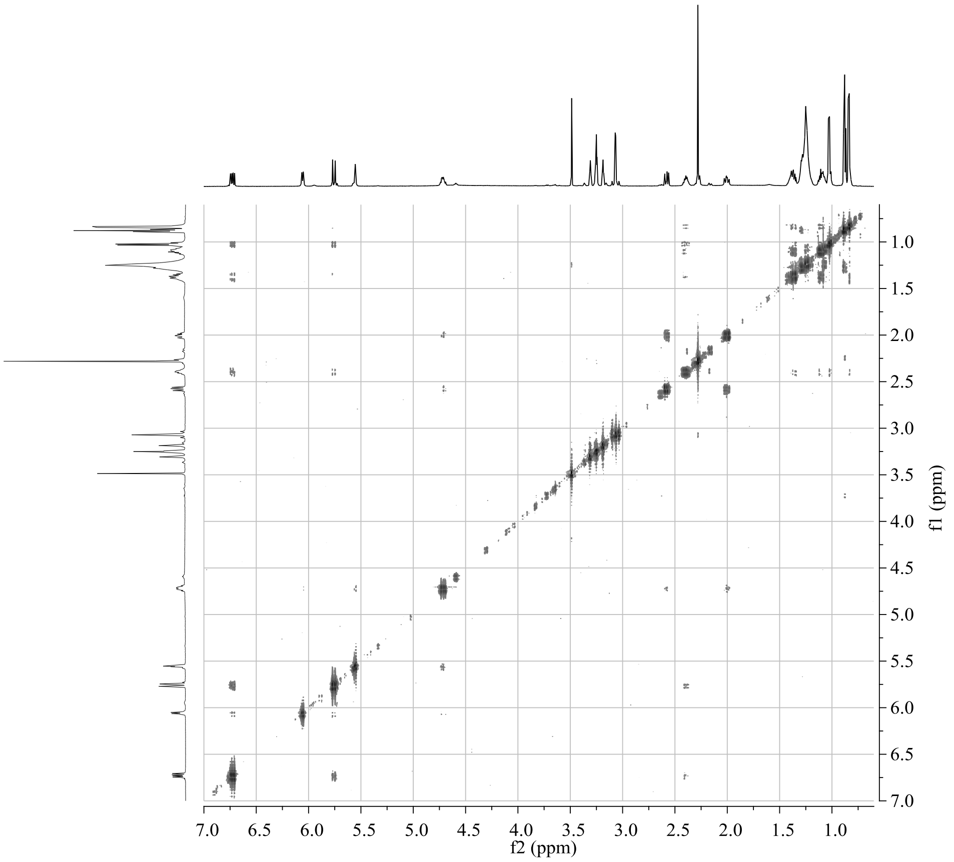

Figure SP1. M: 400 MHz ^{1}H-NMR spectrum of **1** in CD_3OD.

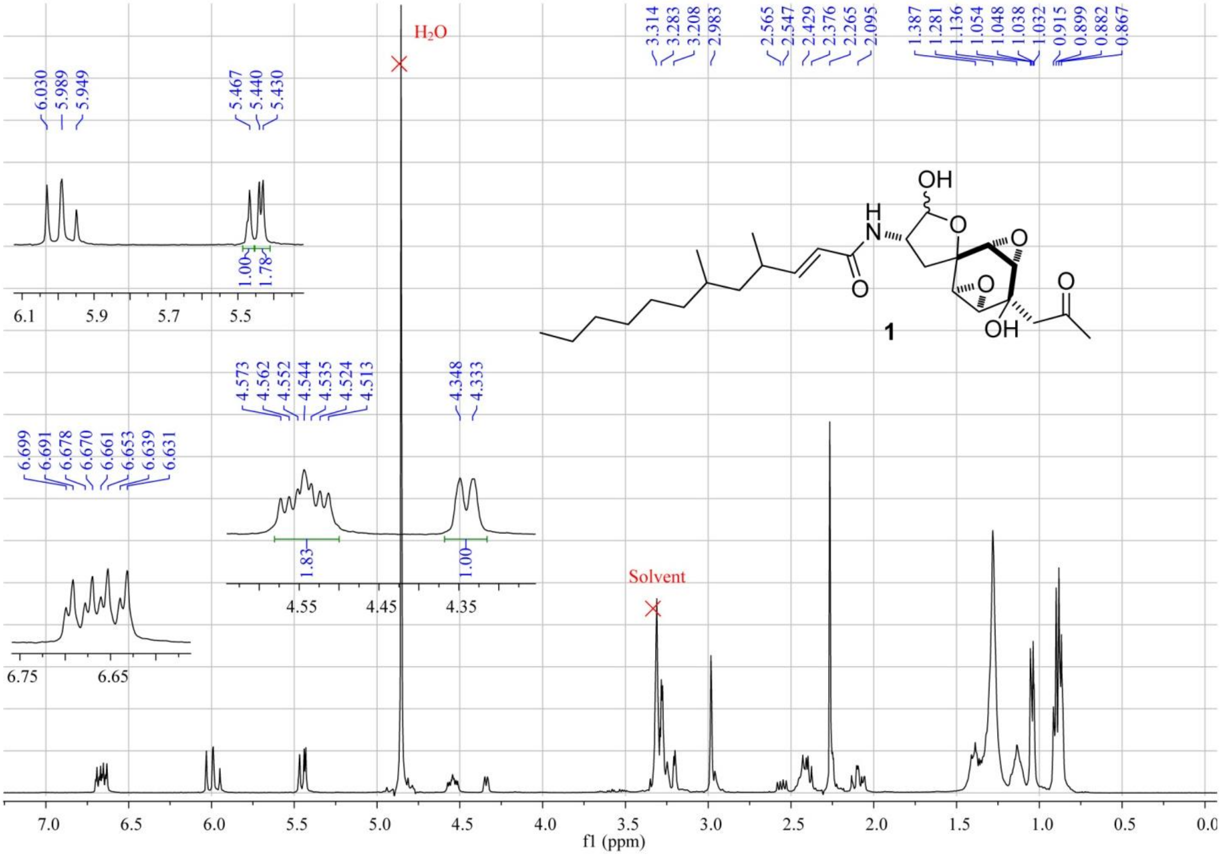

Figure SP1. N: 100 MHz ^{13}C-NMR spectrum of **1** in CD_3OD.

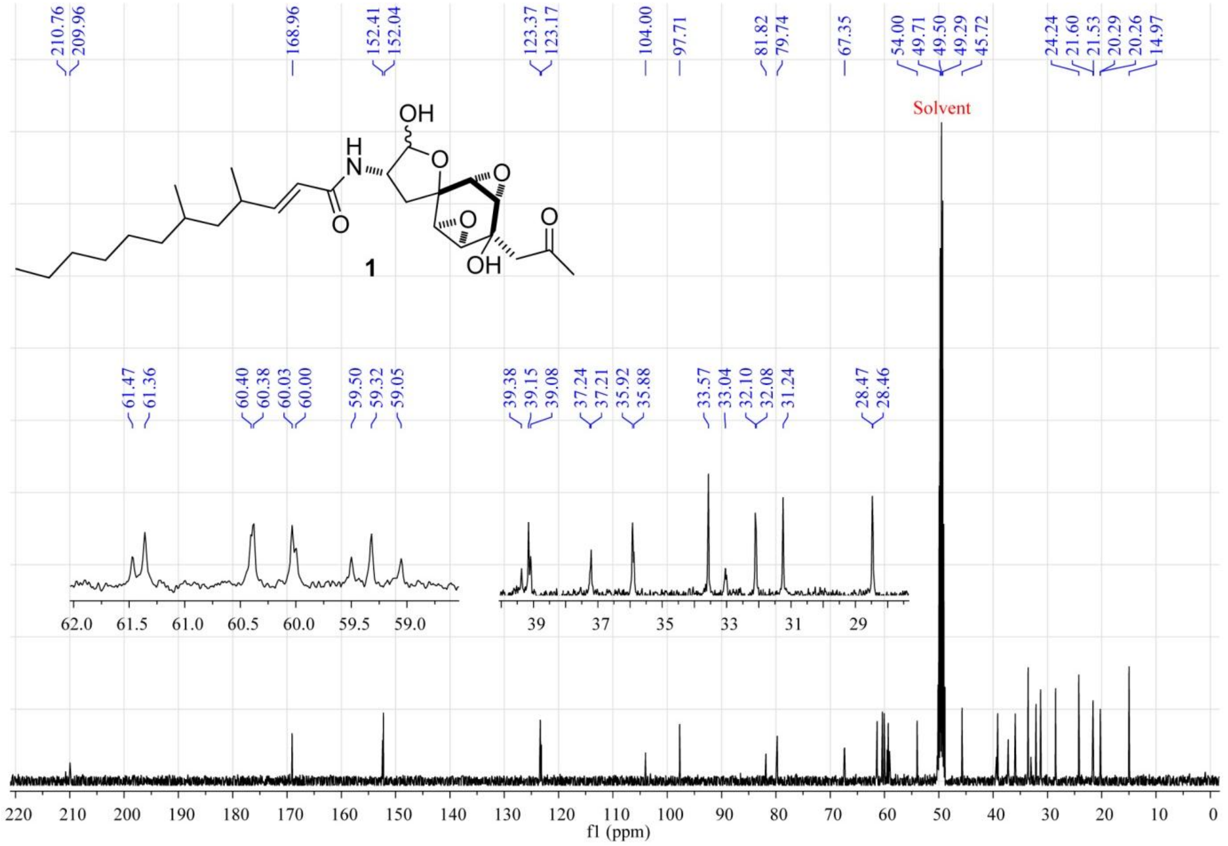

Figure SP1. O: ^{1}H-^{1}H COSY spectrum of **1** in CD_3OD.

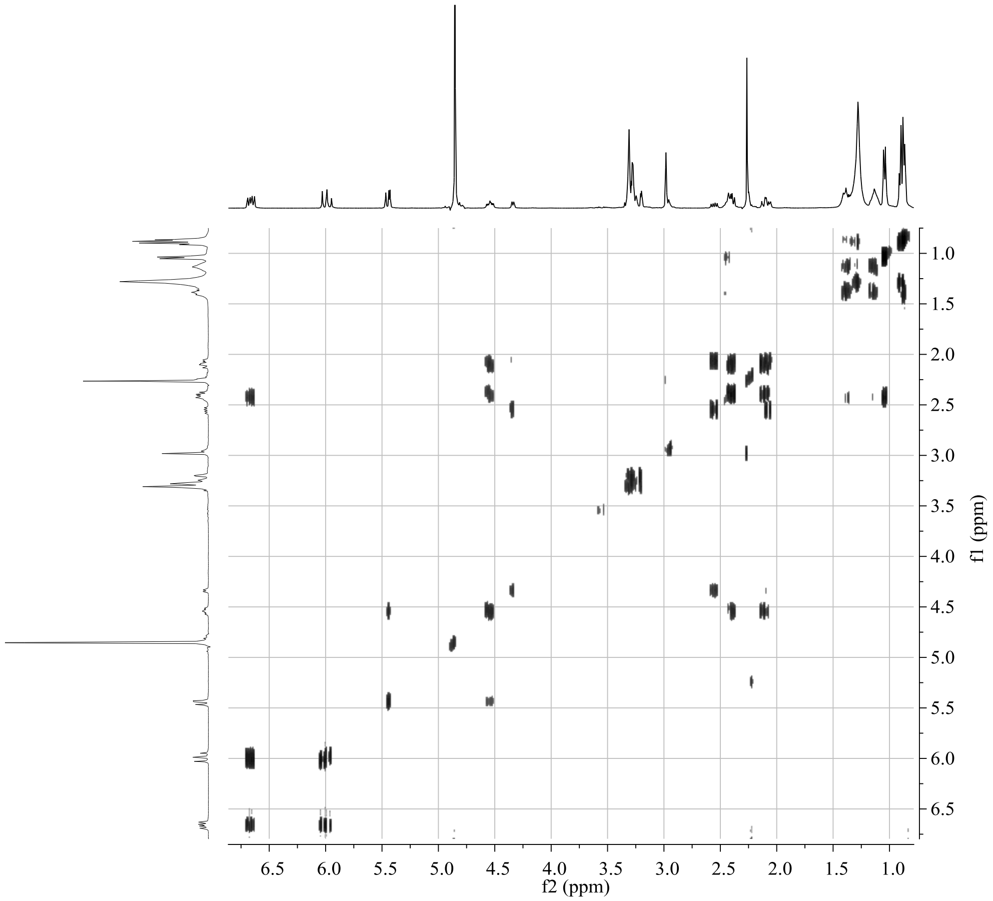

Figure SP1. P: HSQC spectrum of **1** in CD_3OD.

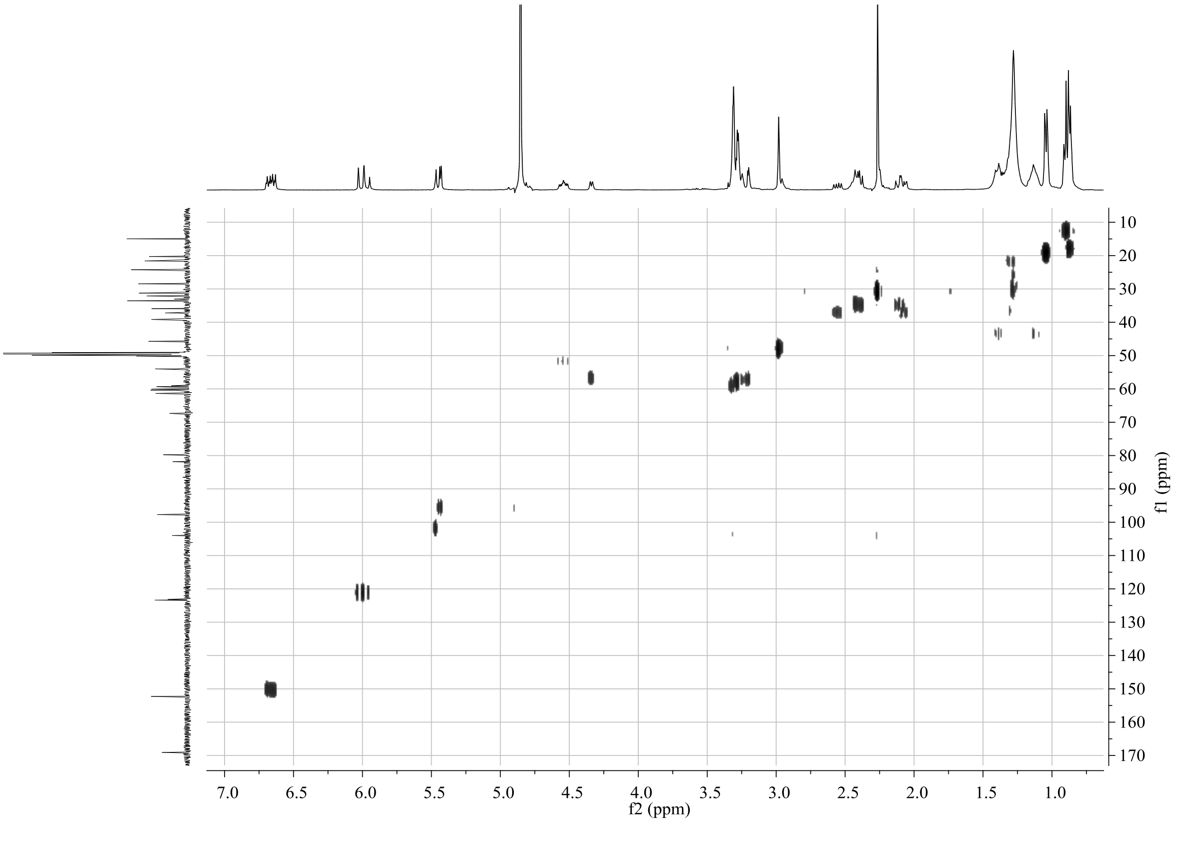

Figure SP1. Q: HMBC spectrum of **1** in CD_3OD.

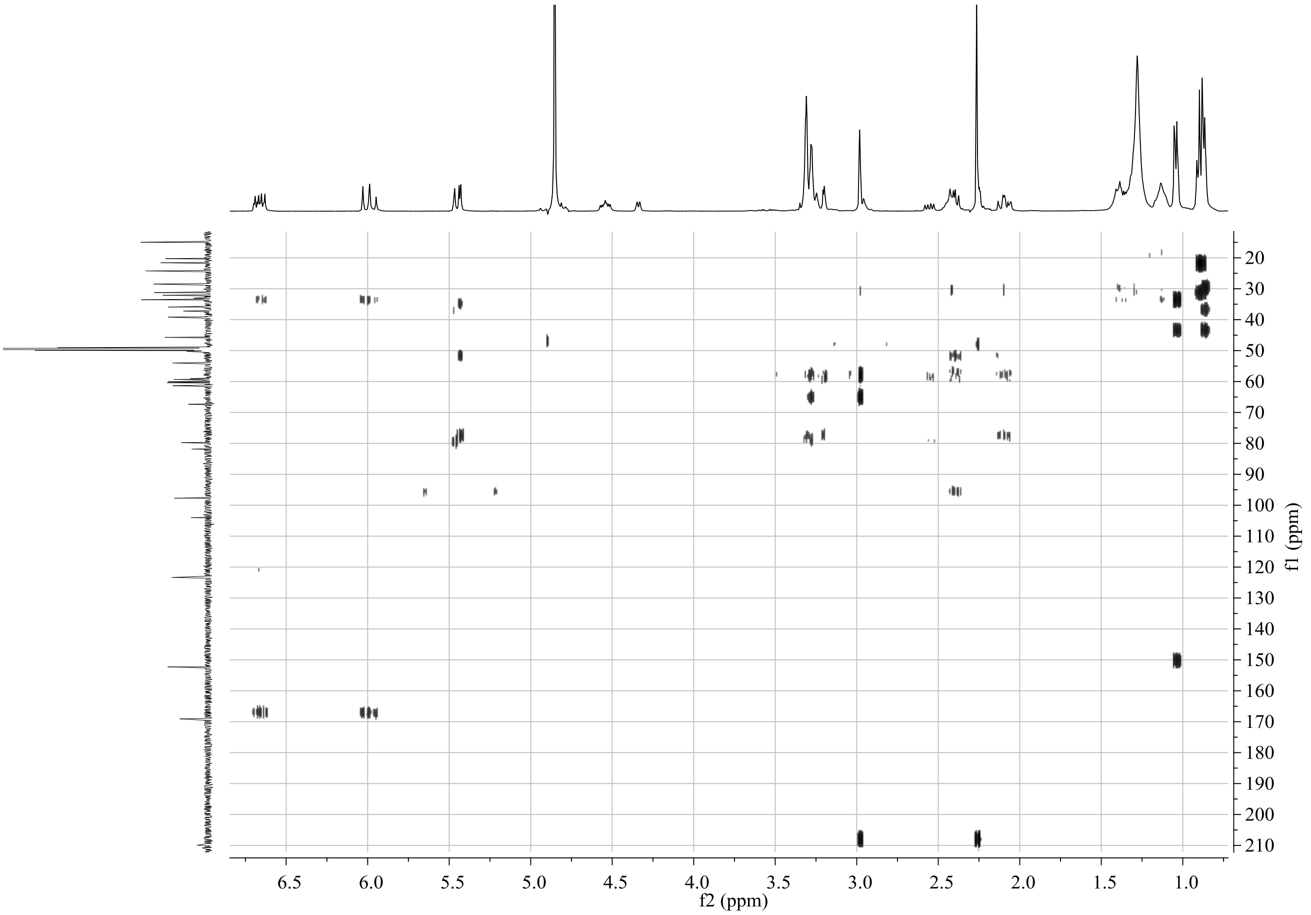

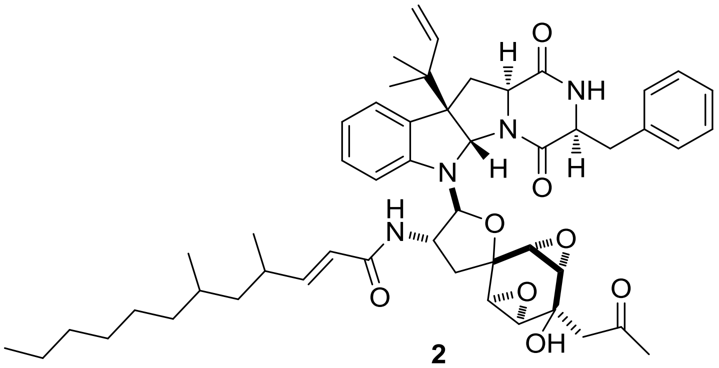

2

Figure SP2. A: Positive (A) and negative (B) ESIMS spectra of **2**.

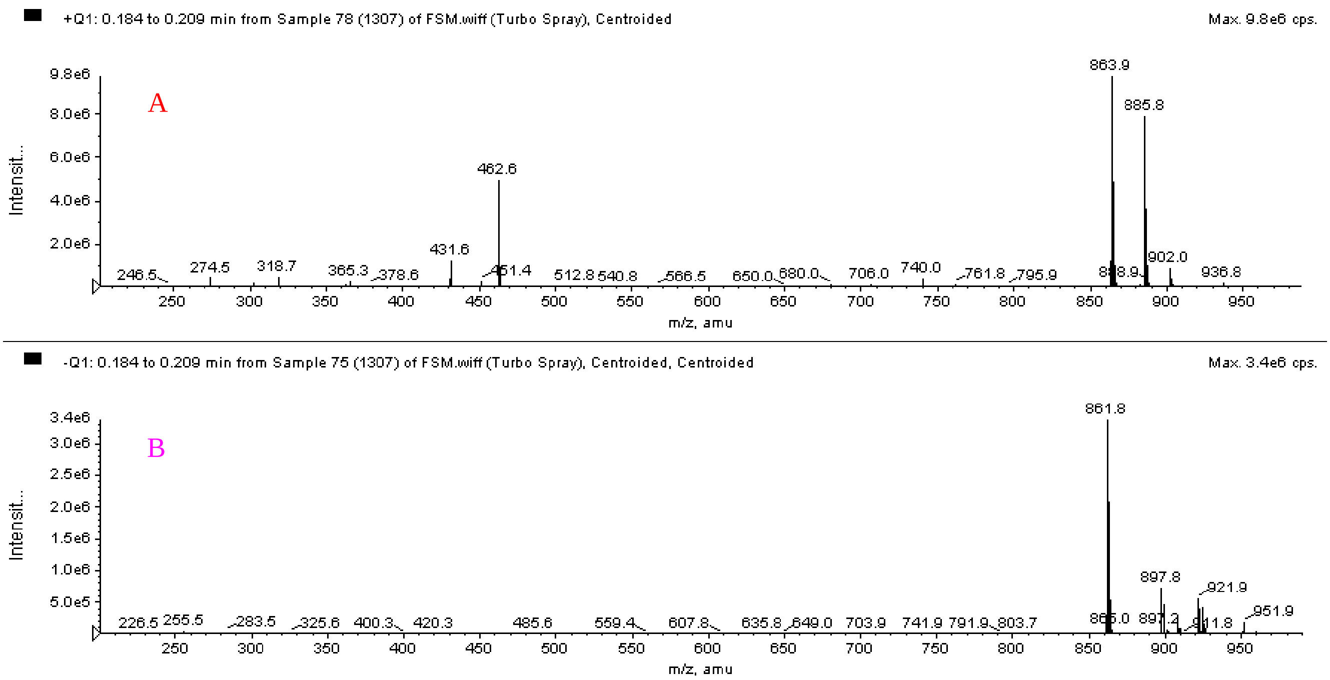

Figure SP2. B: Positive HRESIMS spectrum of **2**.

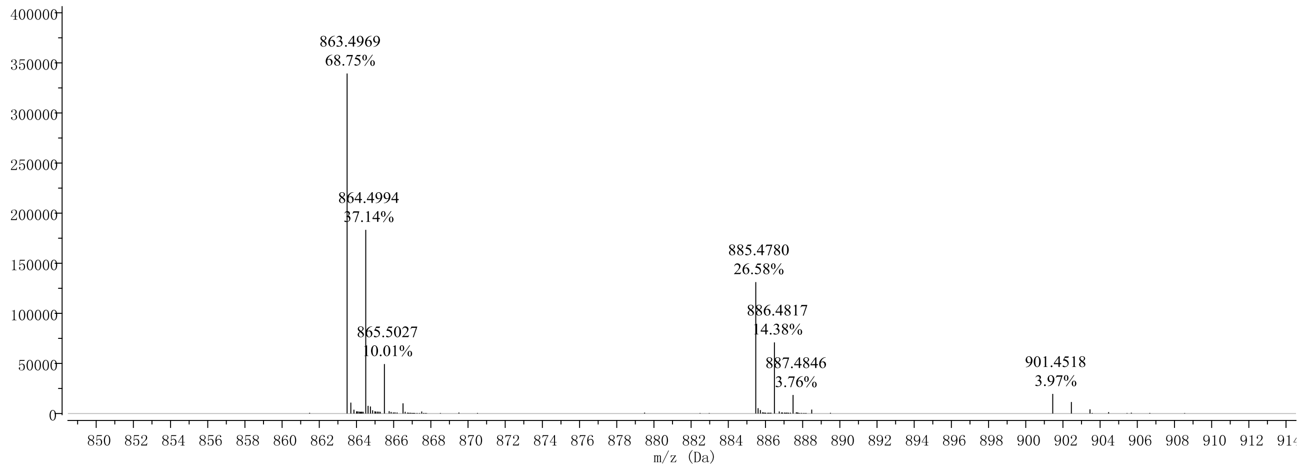

Figure SP2. C: IR spectrum of **2**.

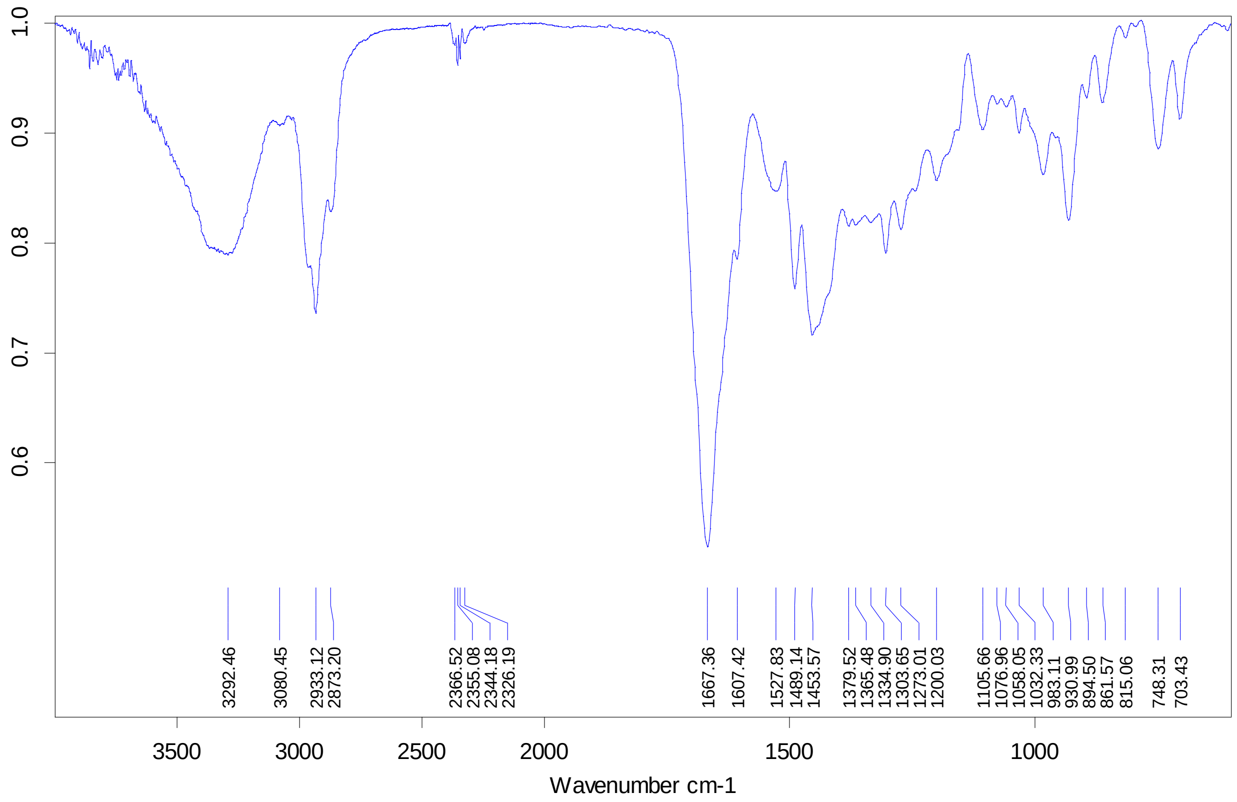

Figure SP2. D: UV spectrum of **2** in MeOH.

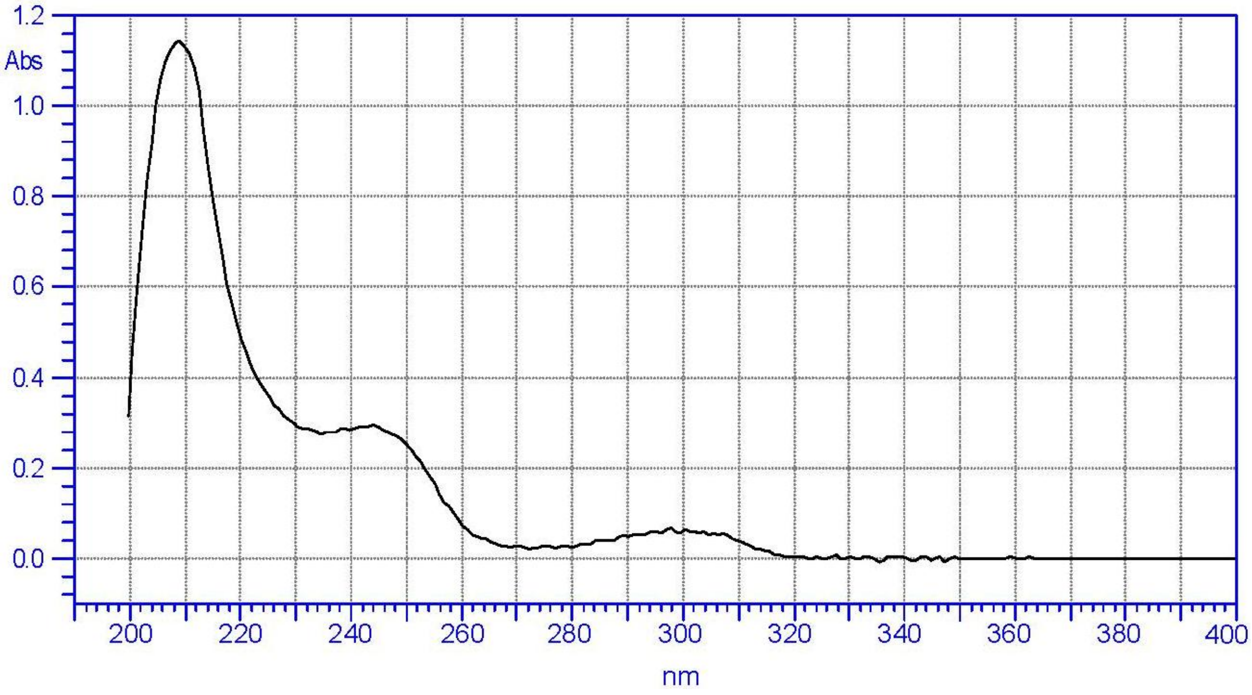

Figure SP2. E: 600 MHz ^{1}H-NMR spectrum of **2** in $CDCl_3$.

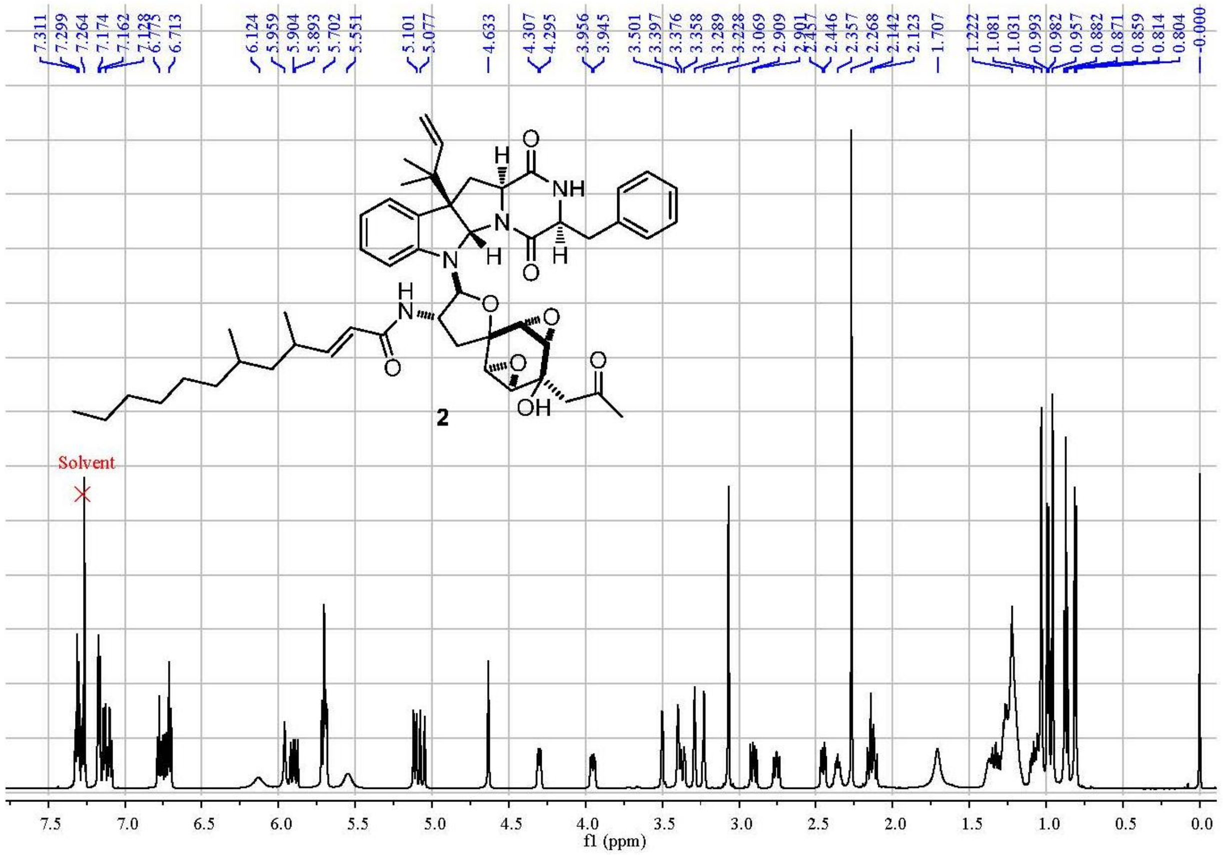

Figure SP2. F: 150 MHz ^{13}C-NMR spectrum of **2** in $CDCl_3$.

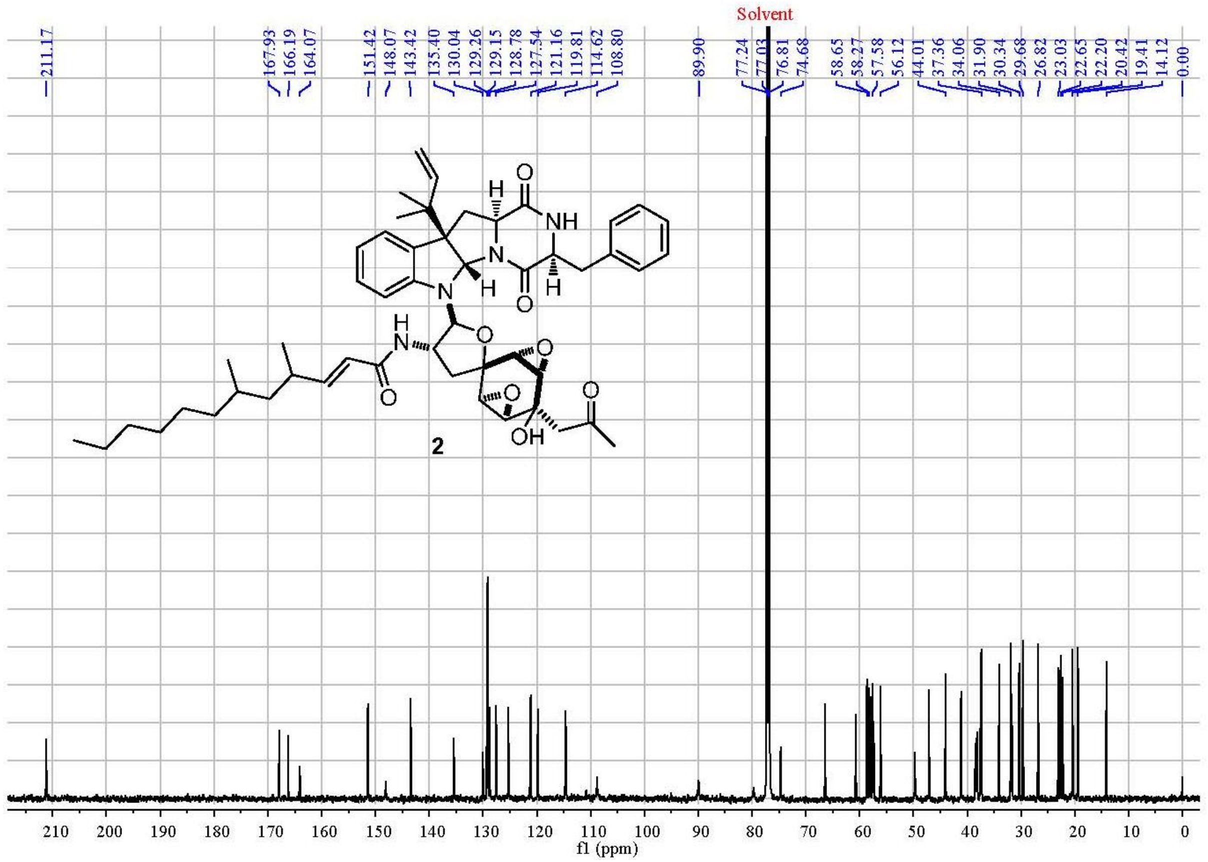

Figure SP2. G: DEPT spectra of **2** in $CDCl_3$.

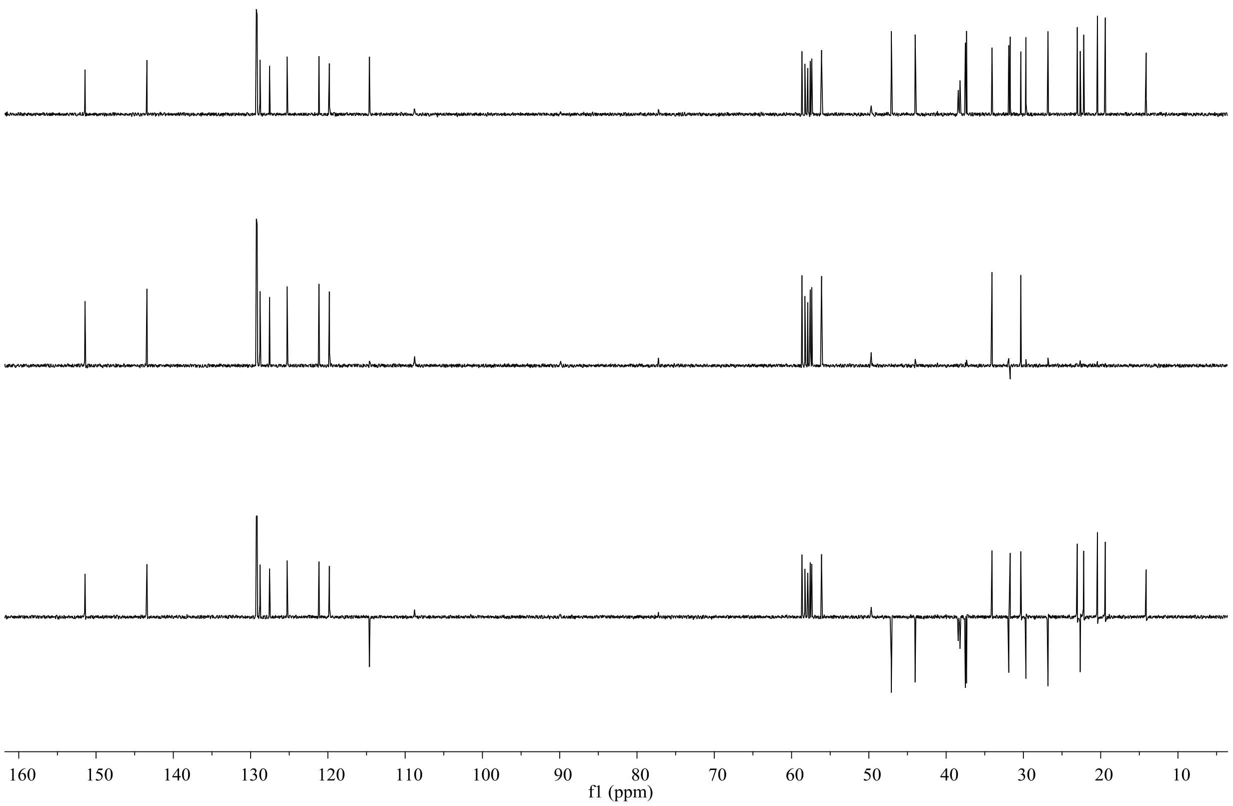

Figure SP2. H: ^1H-^1H COSY spectrum of **2** in $CDCl_3$.

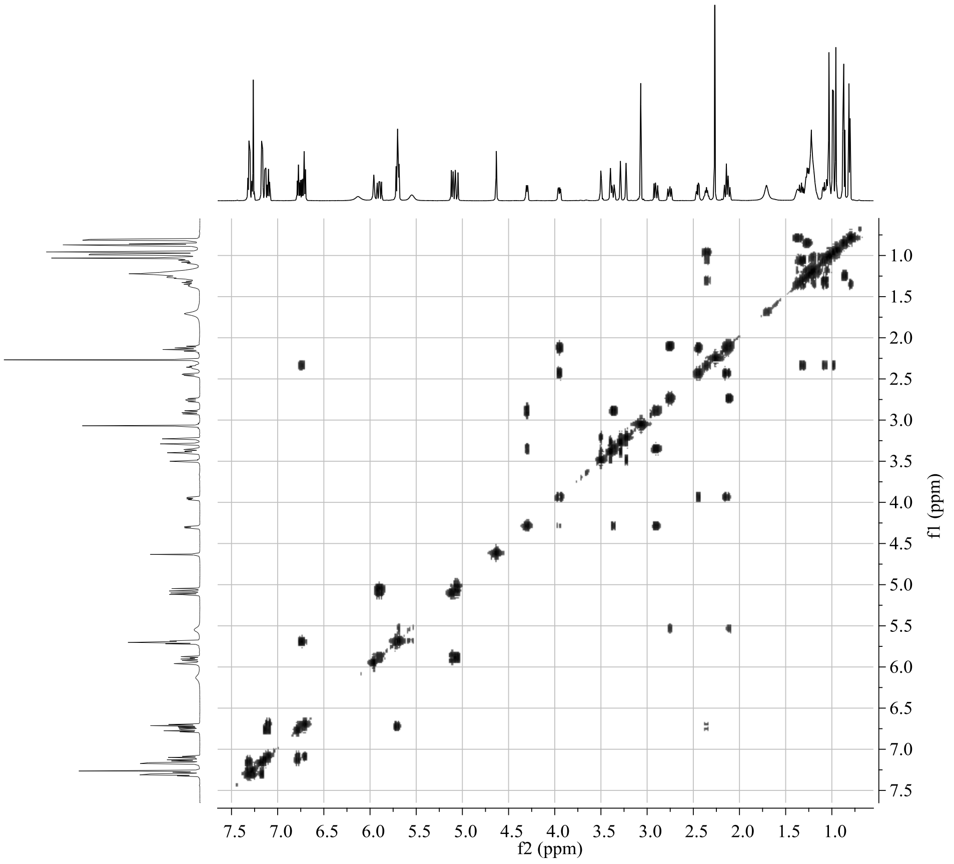

Figure SP2. I: HMQC spectrum of **2** in $CDCl_3$.

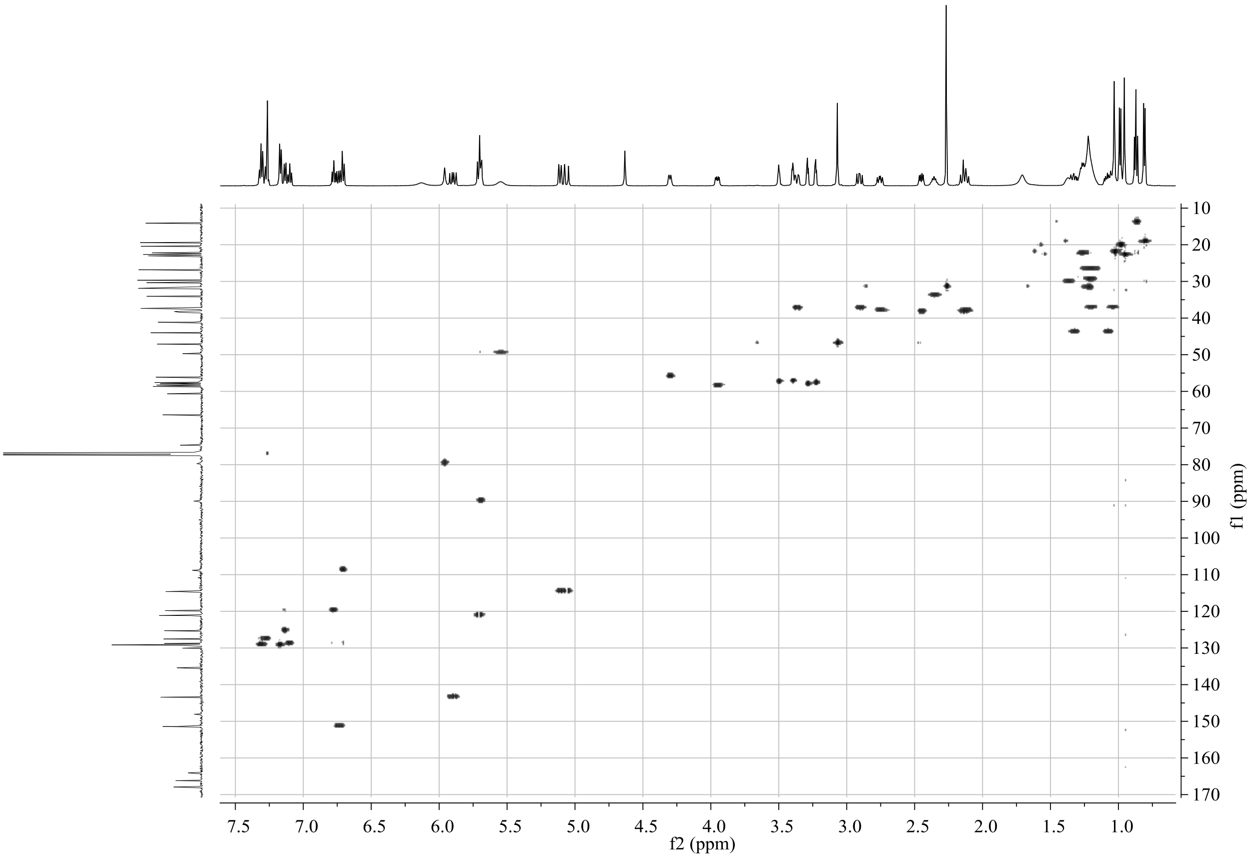

Figure SP2. J: HMBC spectrum of **2** in $CDCl_3$.

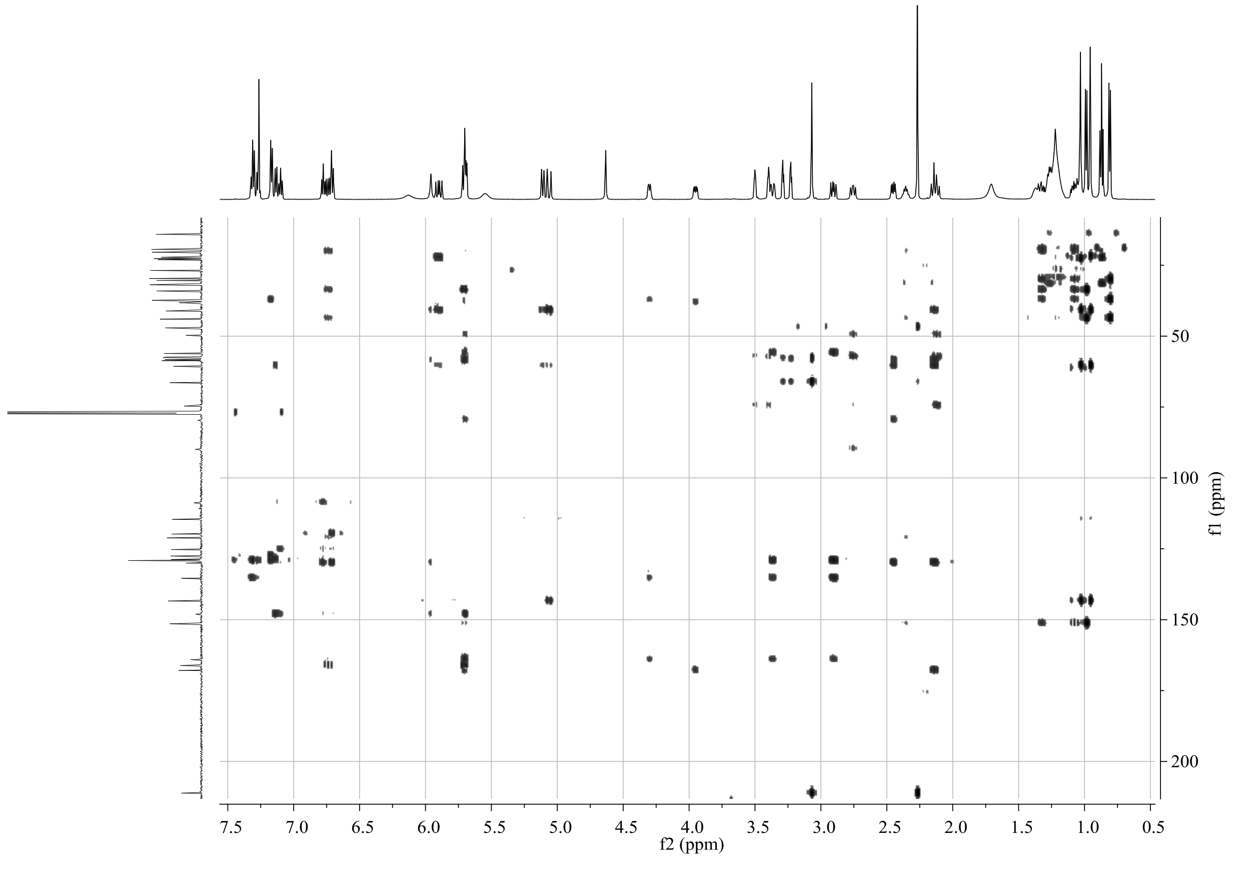

Figure SP2. K: ROESY spectrum of **2** in $CDCl_3$.

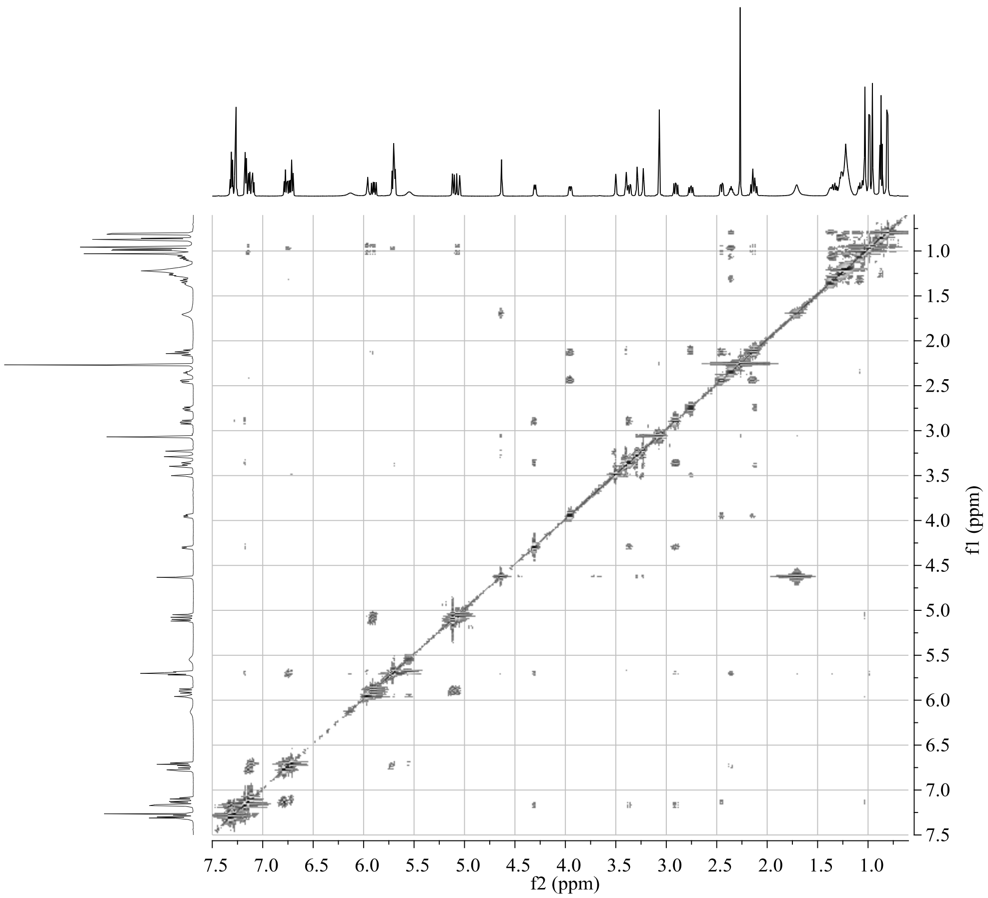

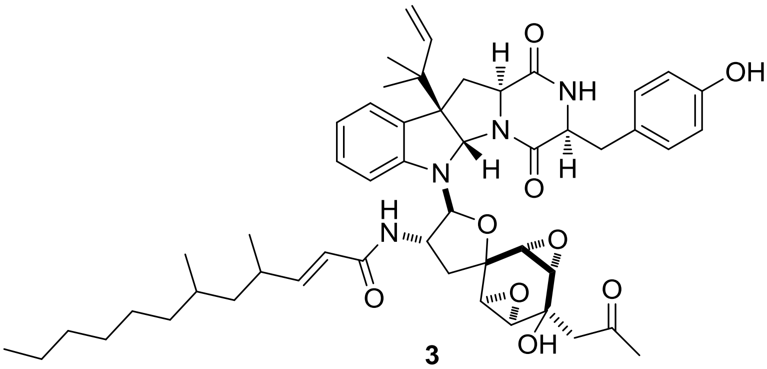

3

Figure SP3. A: Positive (A) and negative (B) ESIMS spectra of **3**.

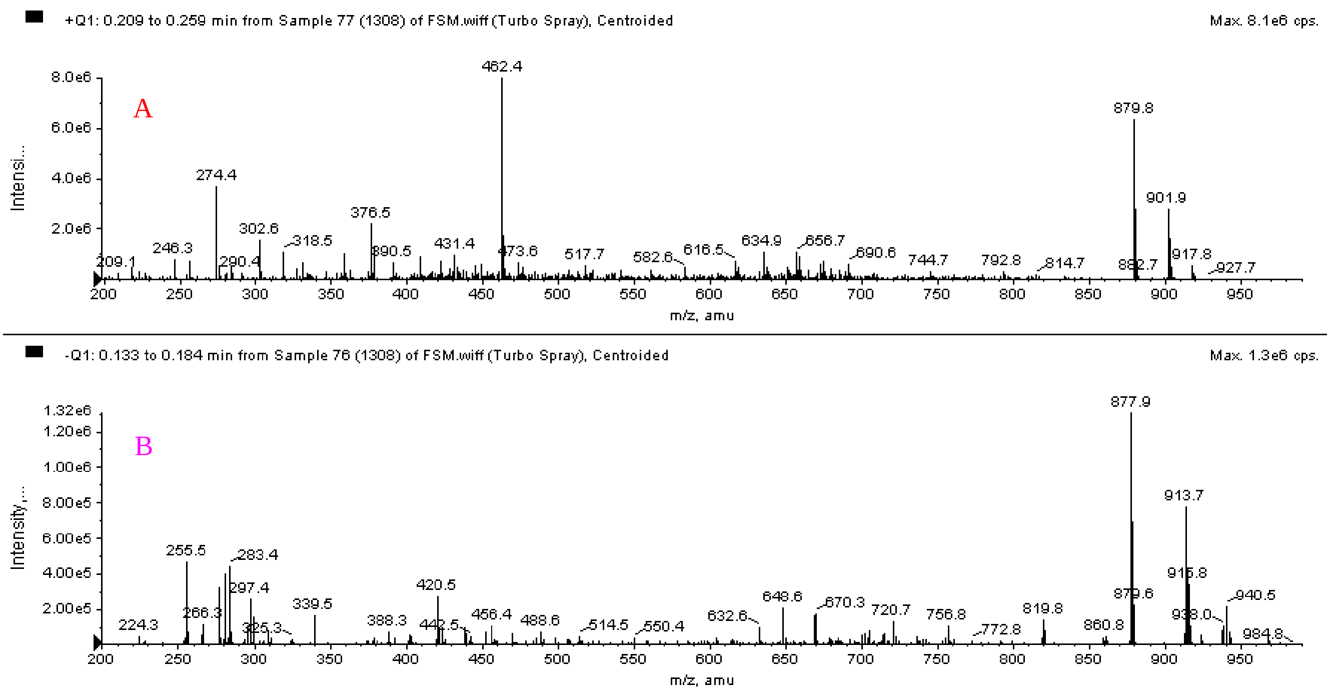

Figure SP3. B: Positive HRESIMS spectrum of **3**.

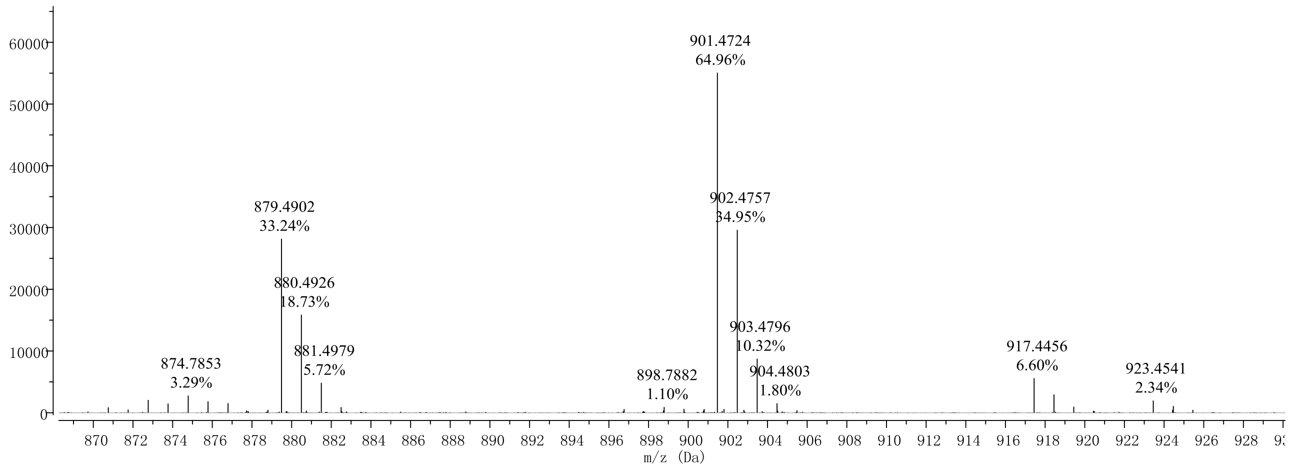

Figure SP3. C: IR spectrum of **3**.

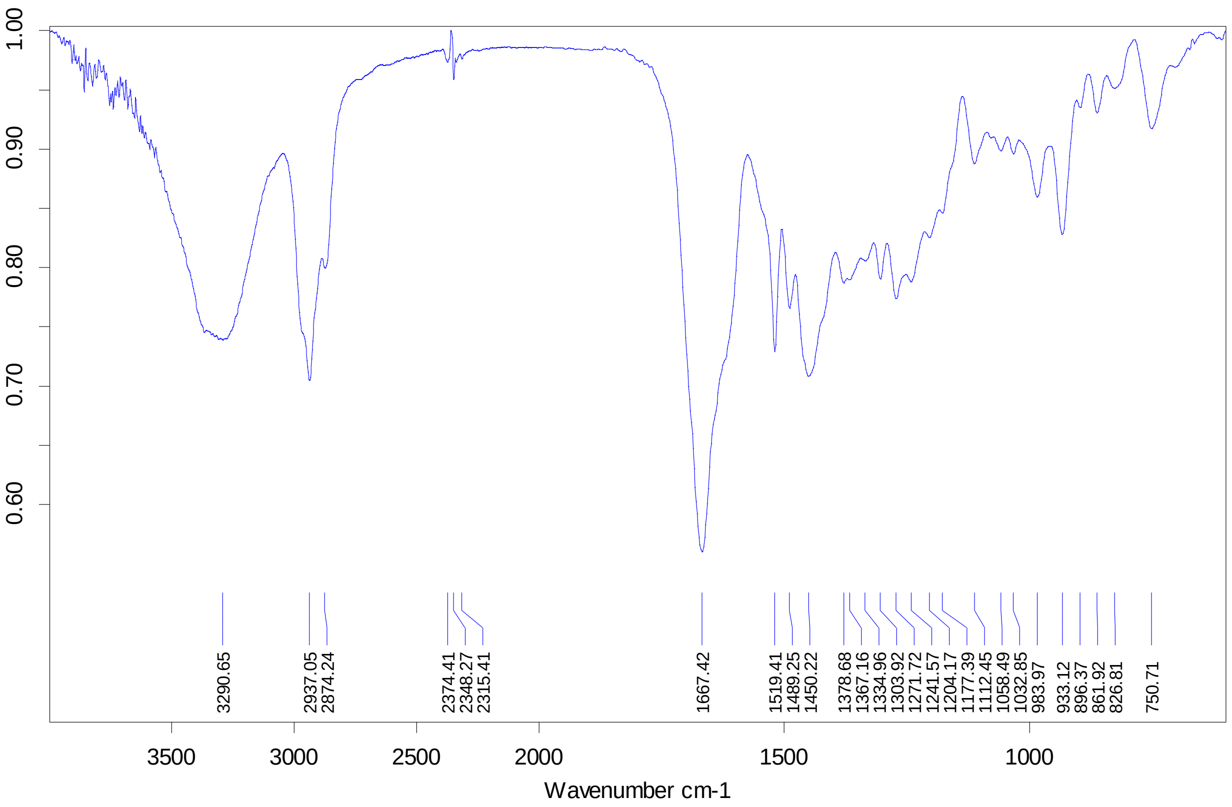

Figure SP3. D: UV spectrum of **3** in MeOH.

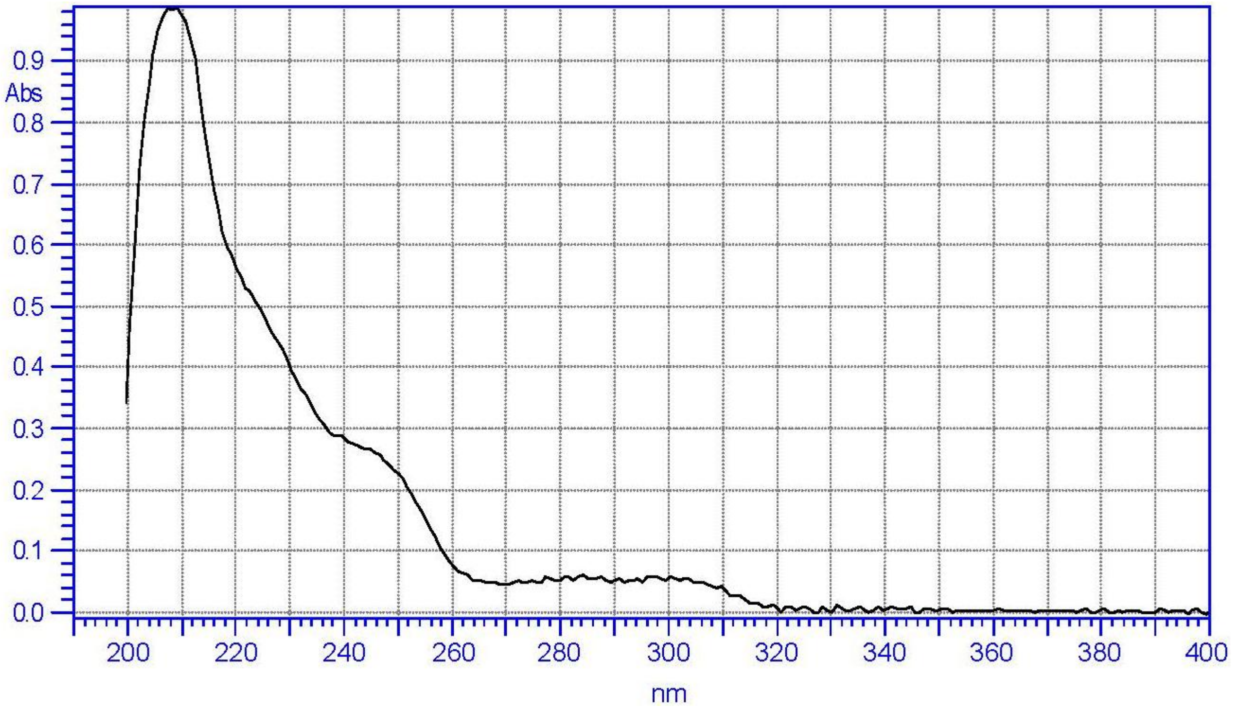

Figure SP3. E: 600 MHz ^{1}H-NMR spectrum of **3** in $CDCl_3$.

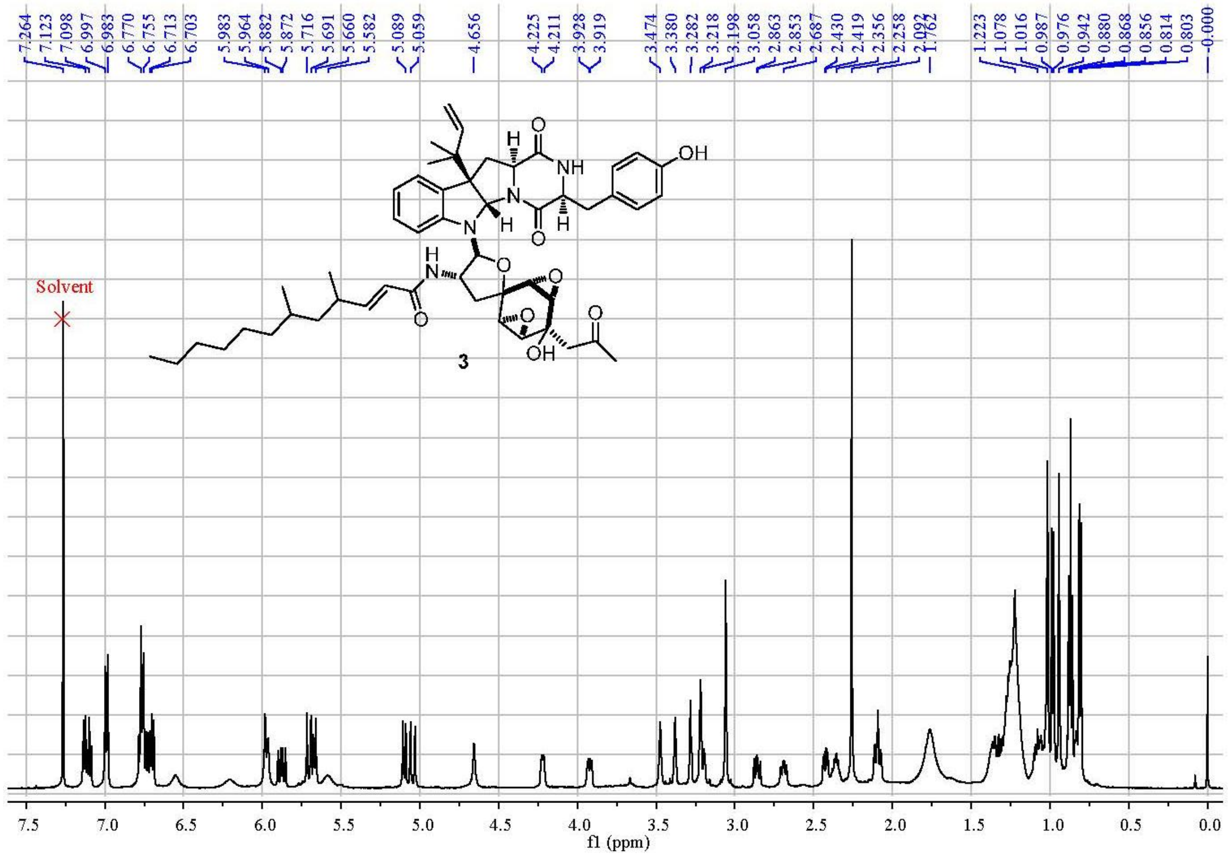

Figure SP3. F: 150 MHz ^{13}C-NMR spectrum of **3** in $CDCl_3$.

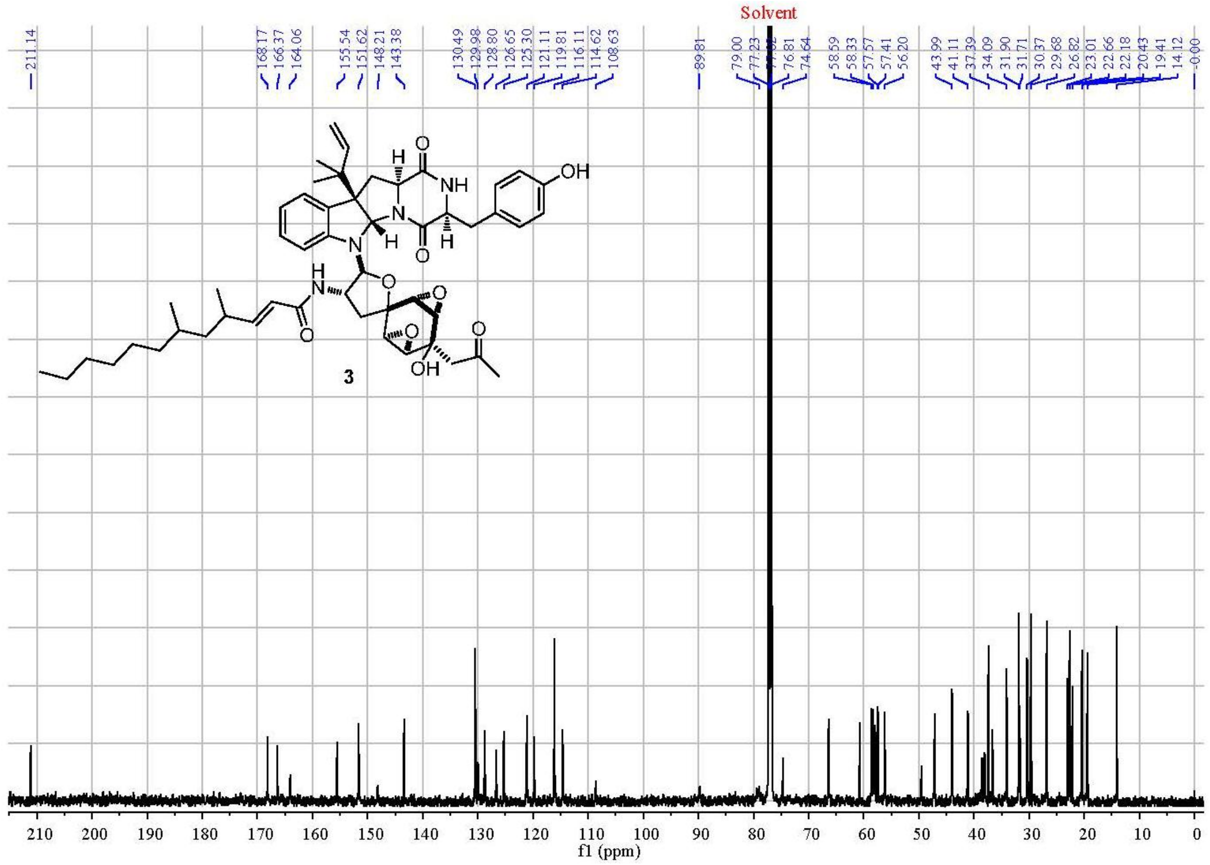

Figure SP3. G: DEPT spectra of **3** in $CDCl_3$.

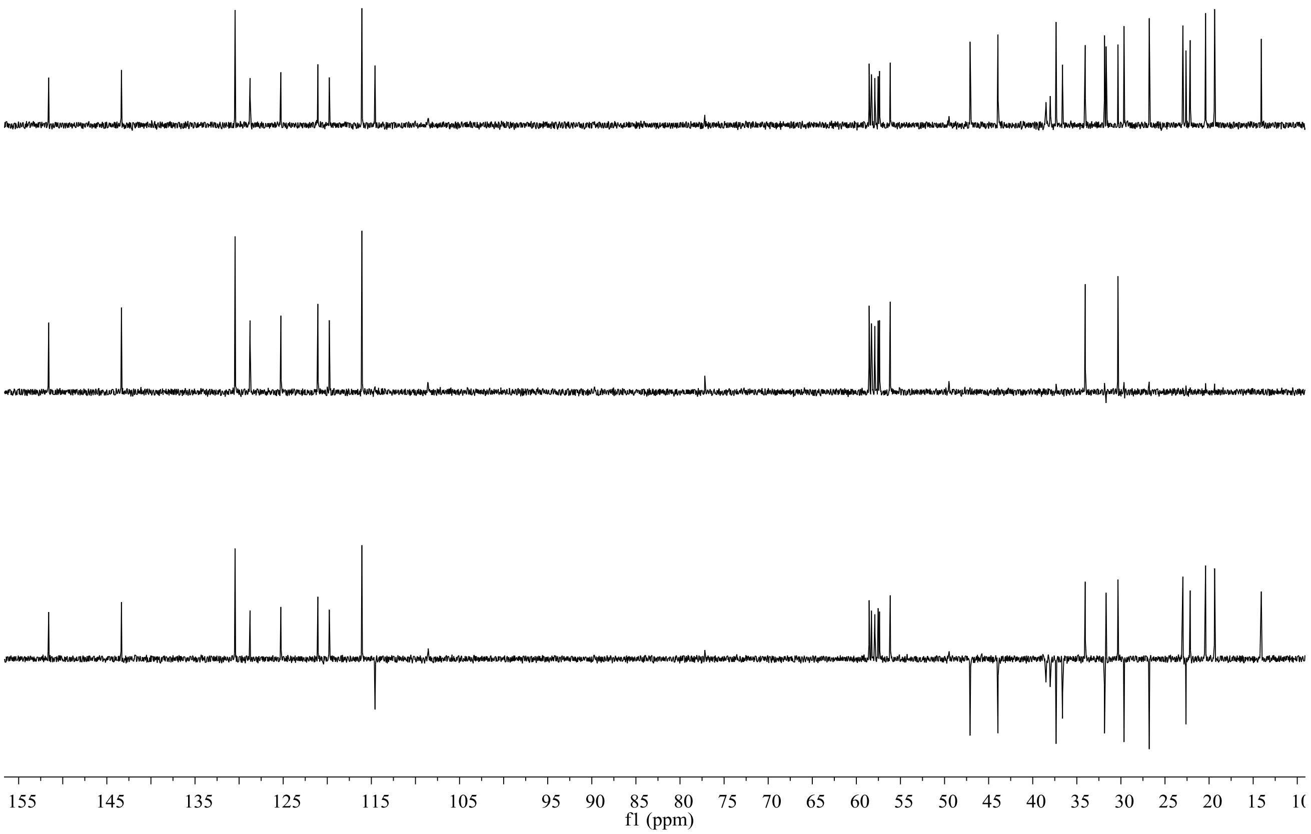

Figure SP3. H: ^{1}H-^{1}H COSY spectrum of **3** in $CDCl_3$.

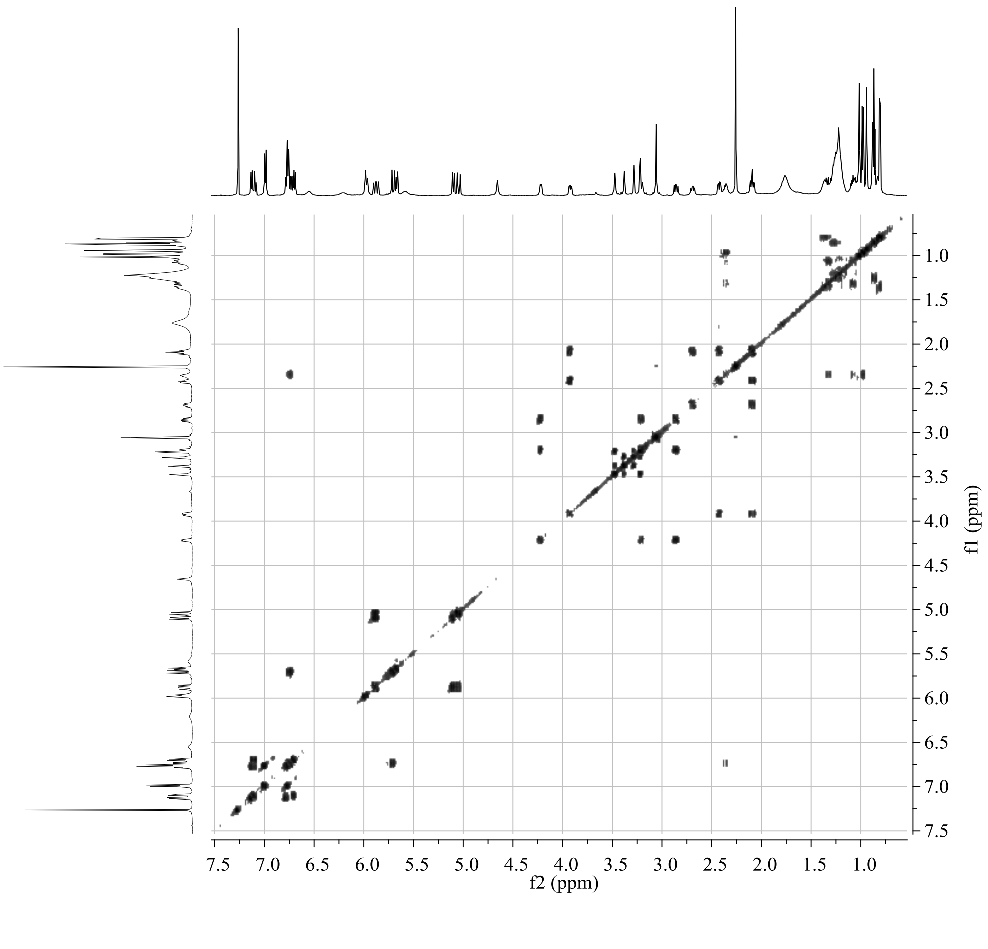

Figure SP3. I: HMQC spectrum of **3** in $CDCl_3$.

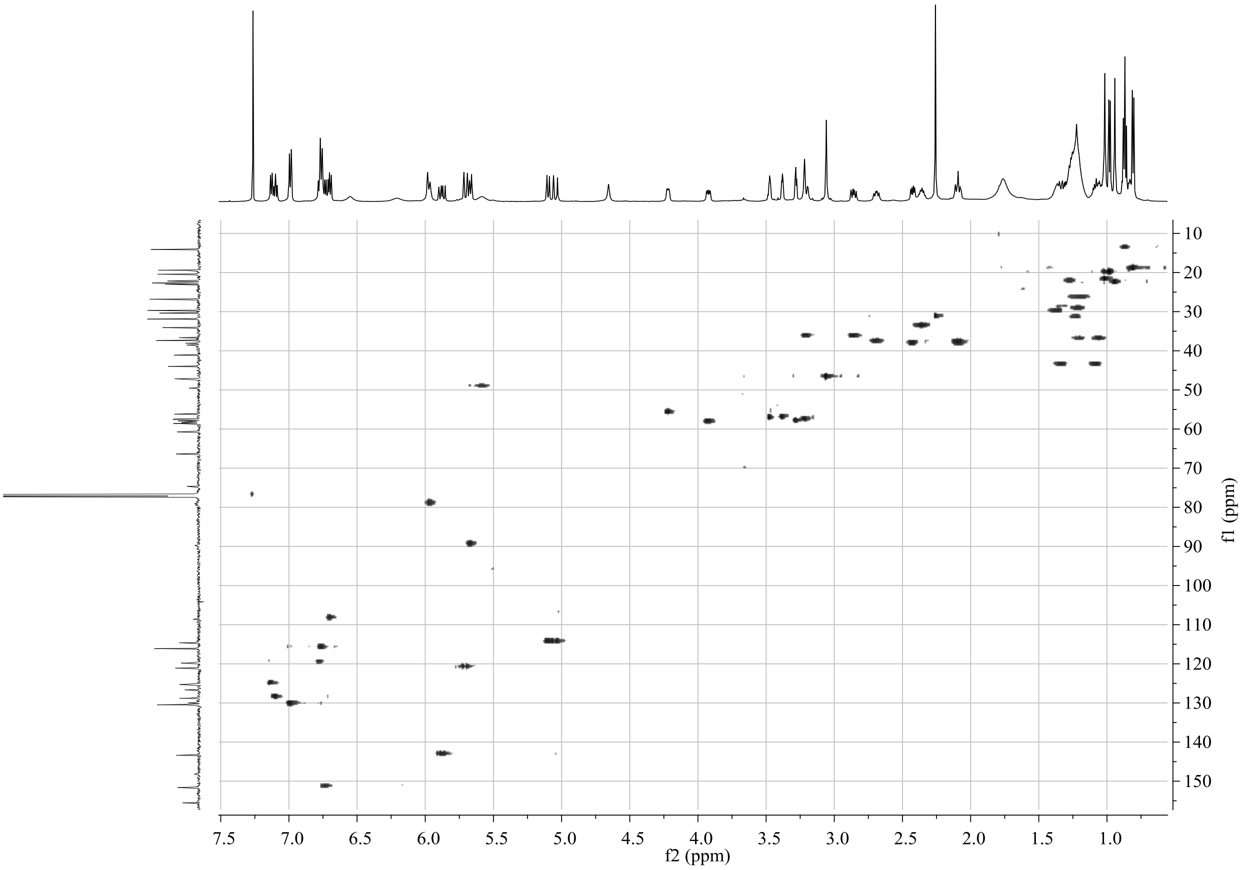

Figure SP3. J: HMBC spectrum of **3** in $CDCl_3$.

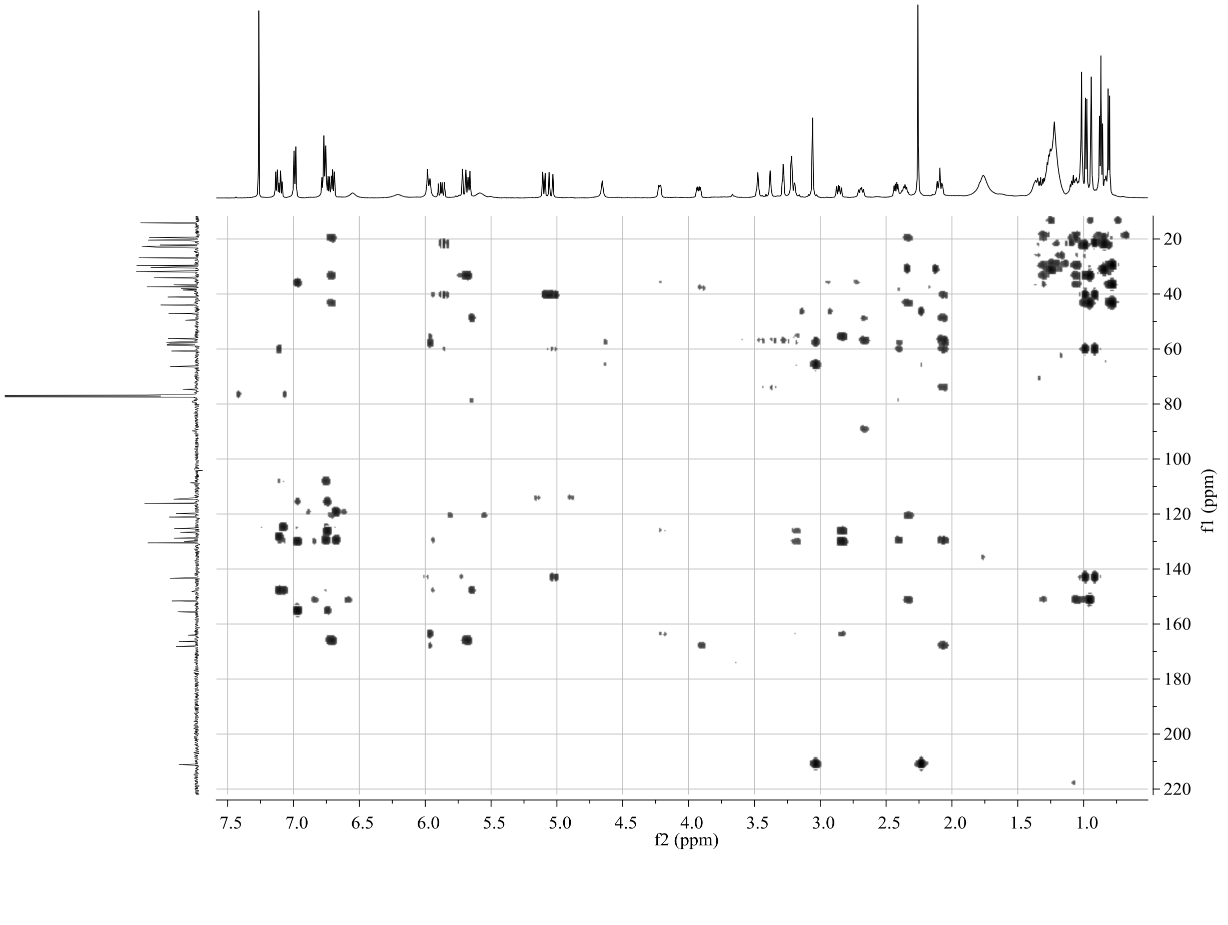

Figure SP3. K: ROESY spectrum of **3** in $CDCl_3$.

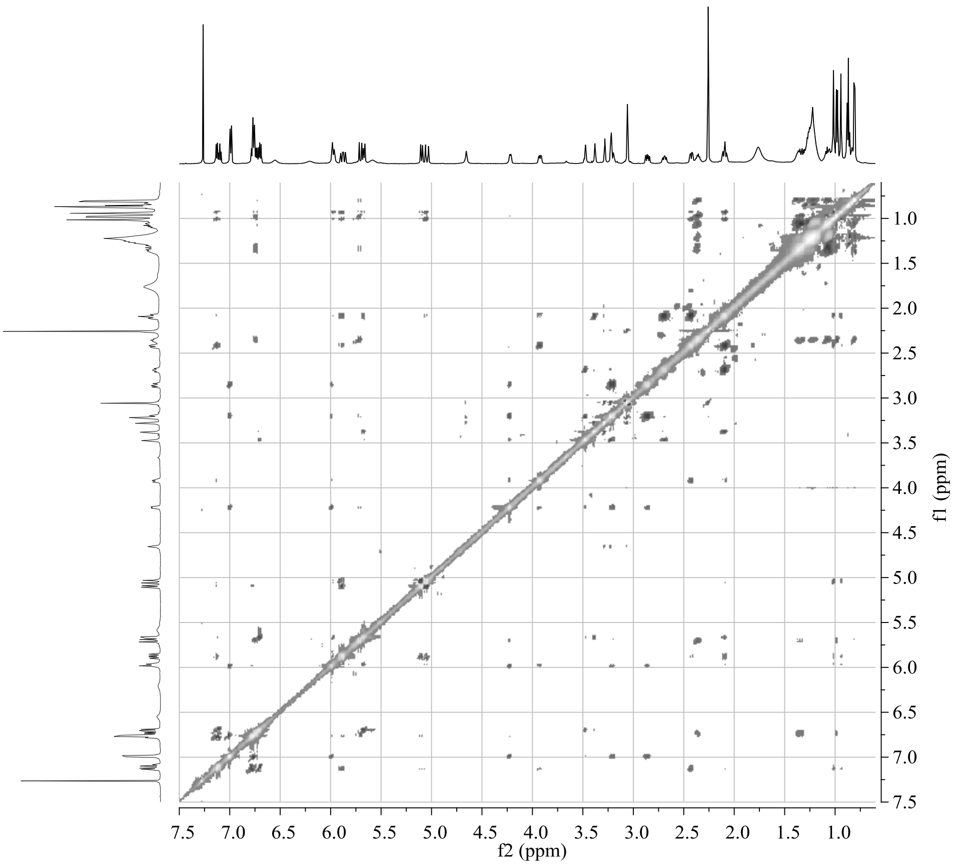

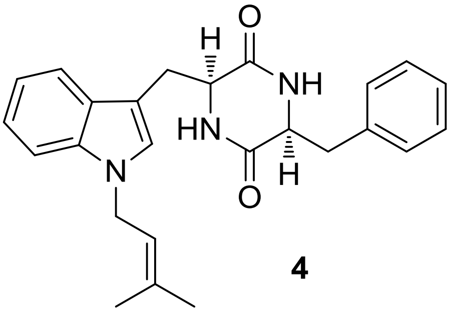

4

Figure SP4. A: Positive (A) and negative (B) ESIMS spectra of **4**.

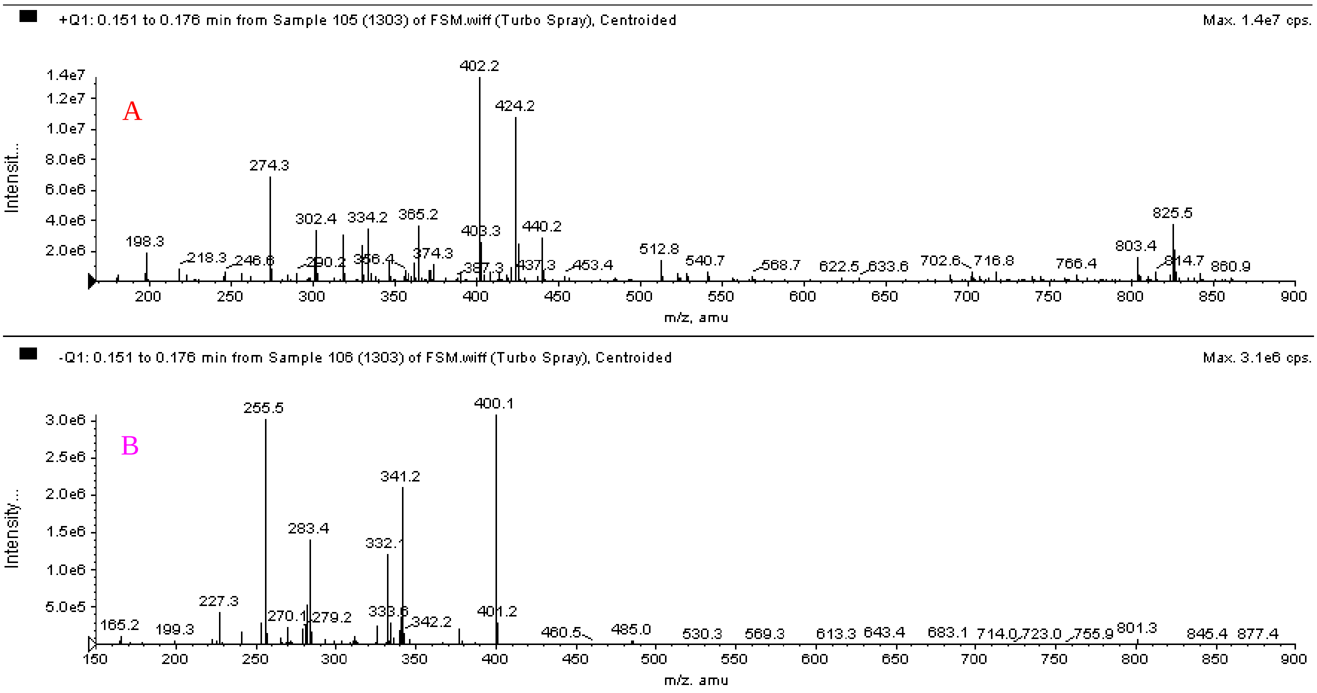

Figure SP4. B: Positive HRESIMS spectrum of **4**.

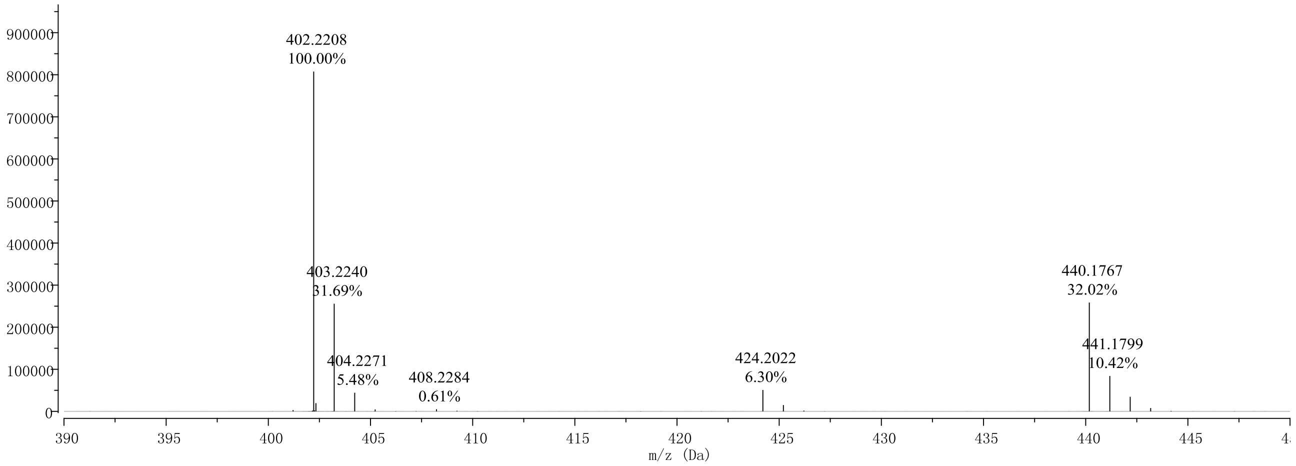

Figure SP4. C: IR spectrum of **4**.

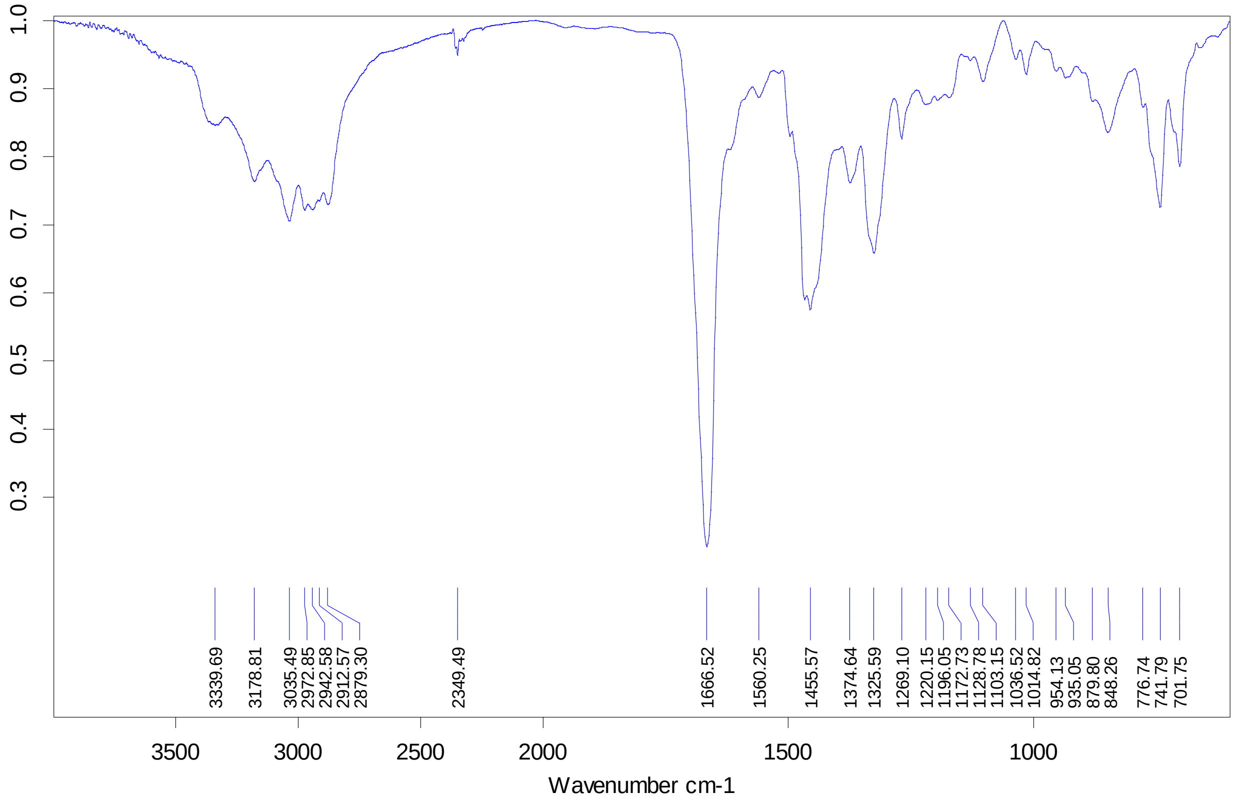

Figure SP4. D: UV spectrum of **4** in MeOH.

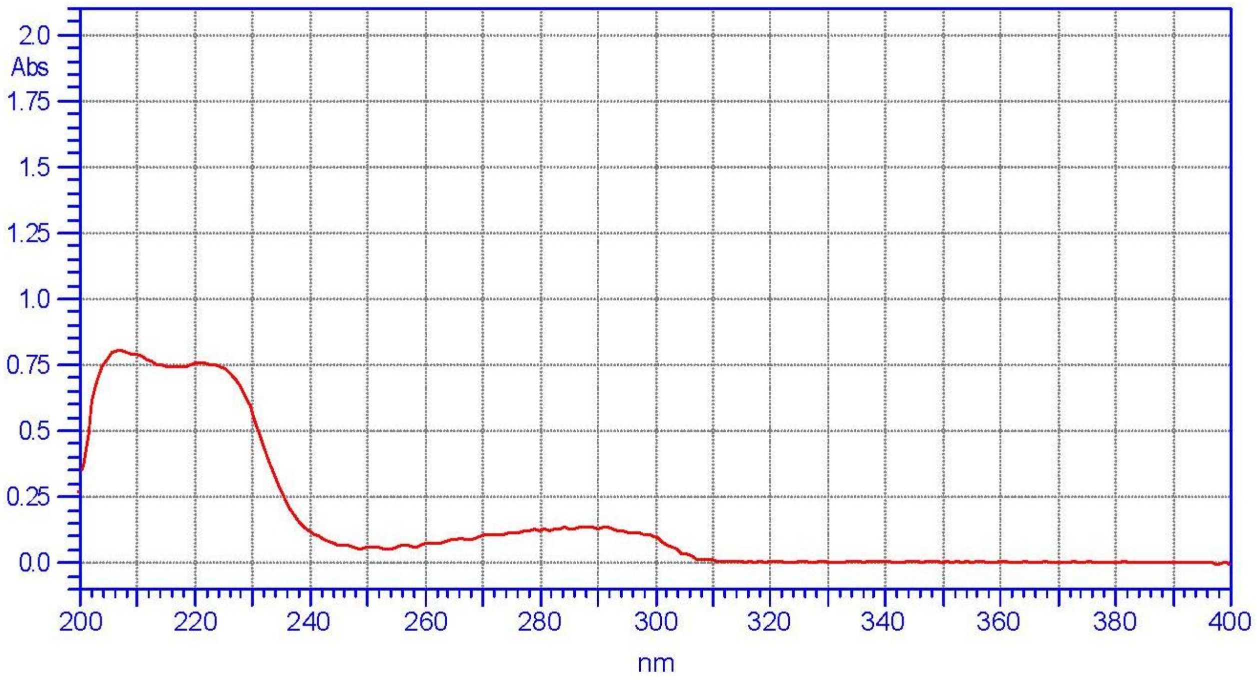

Figure SP4. E: 400 MHz ^{1}H-NMR spectrum of **4** in DMSO-d_6.

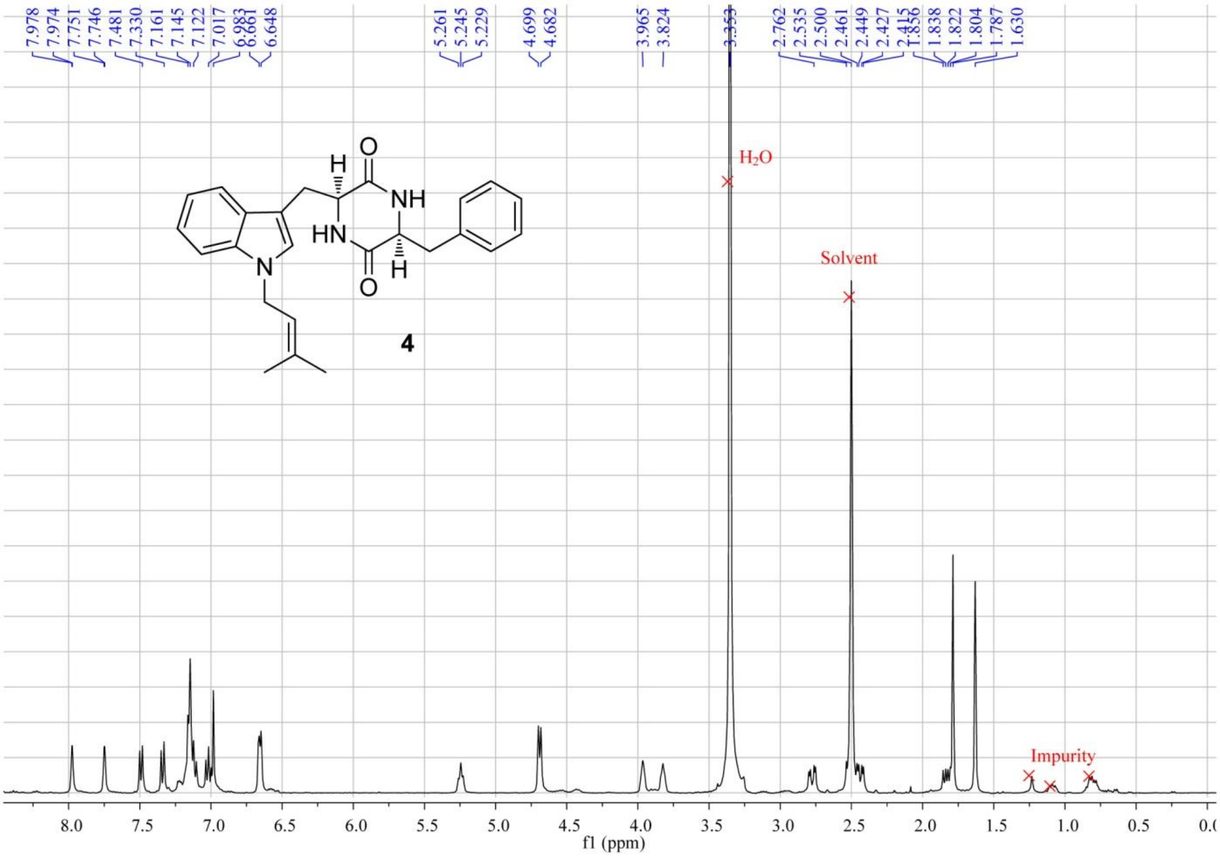

Figure SP4. F: 100 MHz ^{13}C-NMR spectrum of **4** in DMSO-d_6.

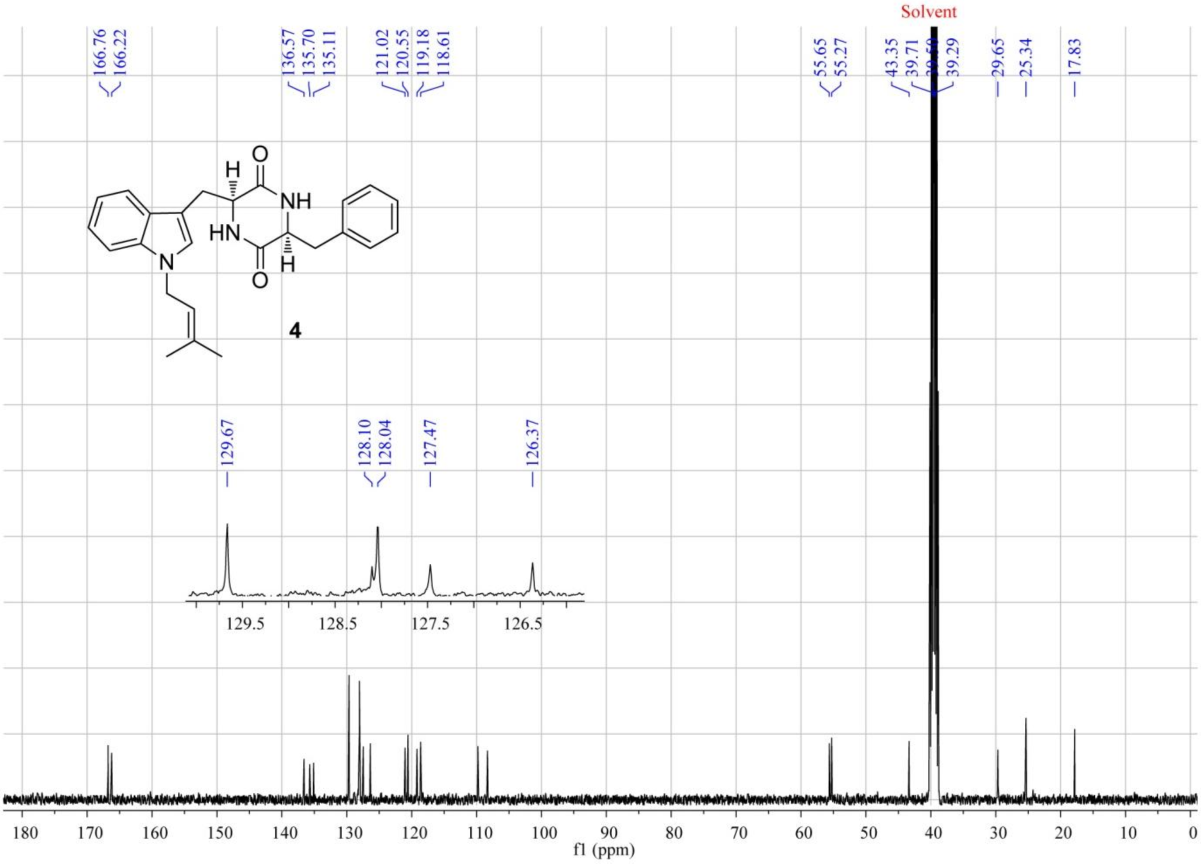

Figure SP4. G: ^{1}H-^{1}H COSY spectrum of **4** in DMSO-d_6.

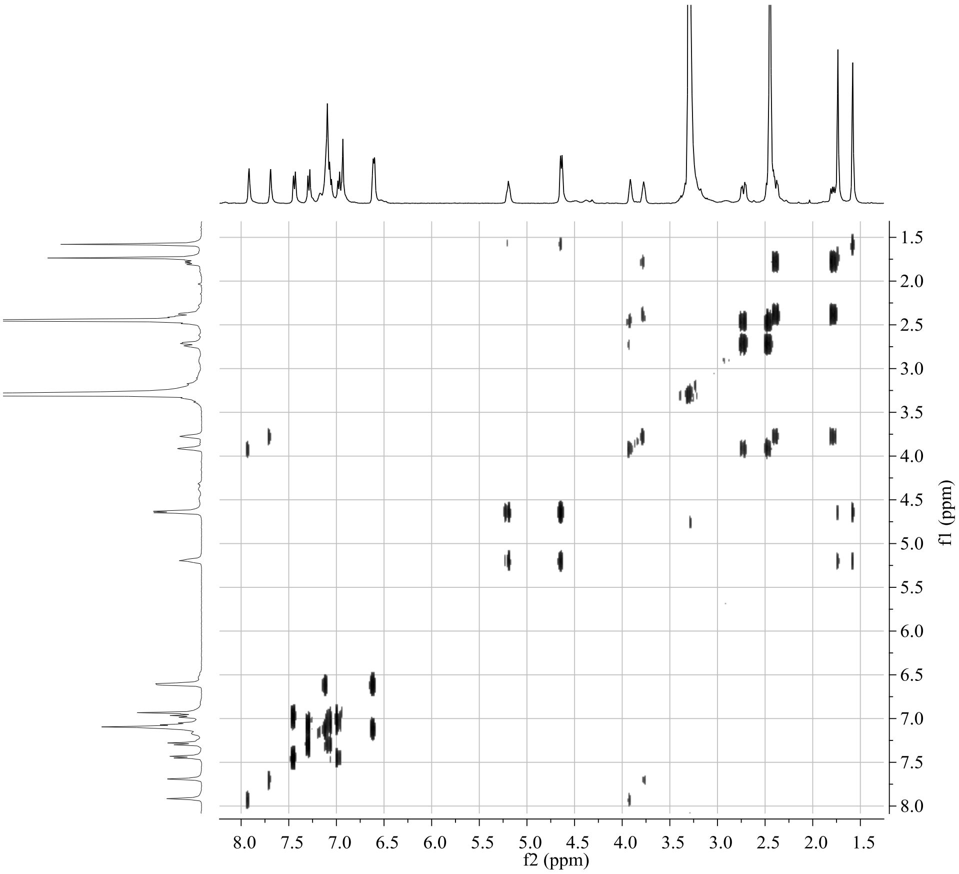

Figure SP4. H: HMQC spectrum of **4** in DMSO-d_6.

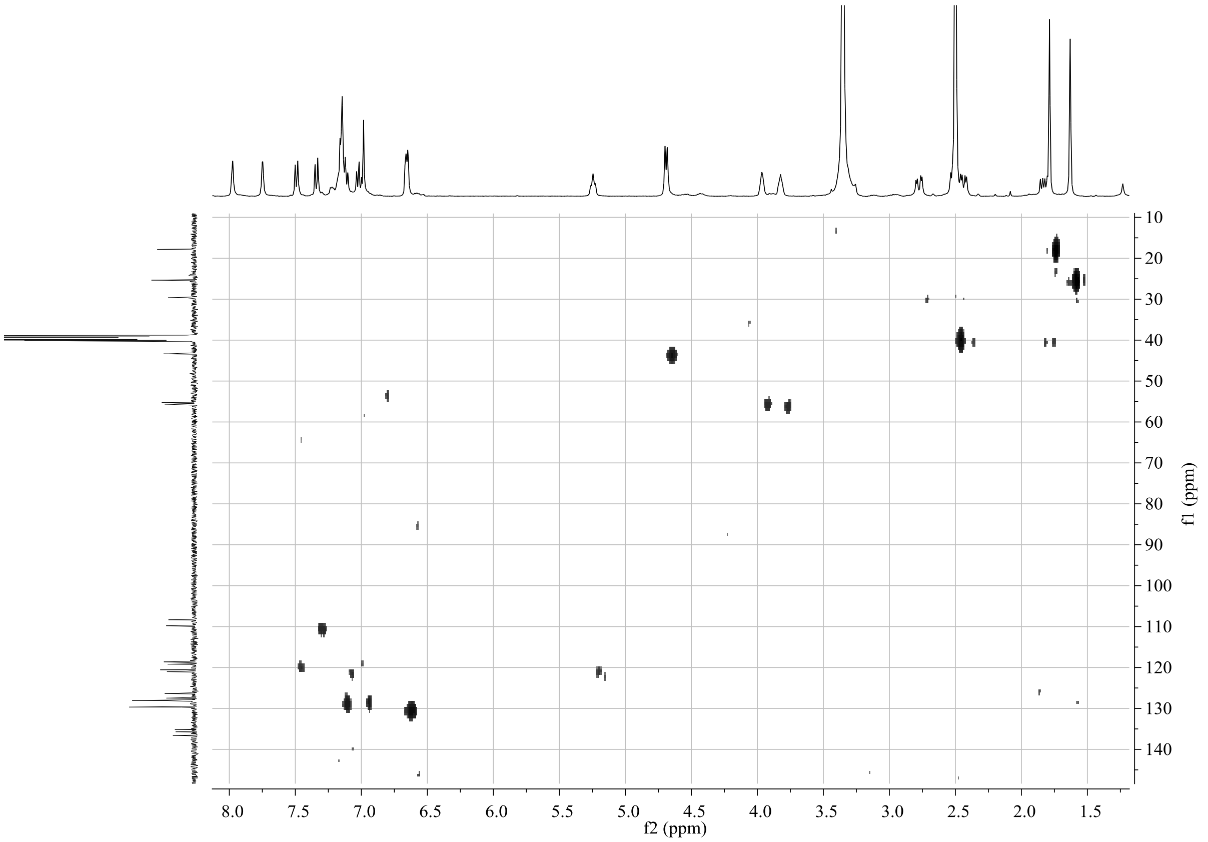

Figure SP4. I: HMBC spectrum of **4** in DMSO-d_6.

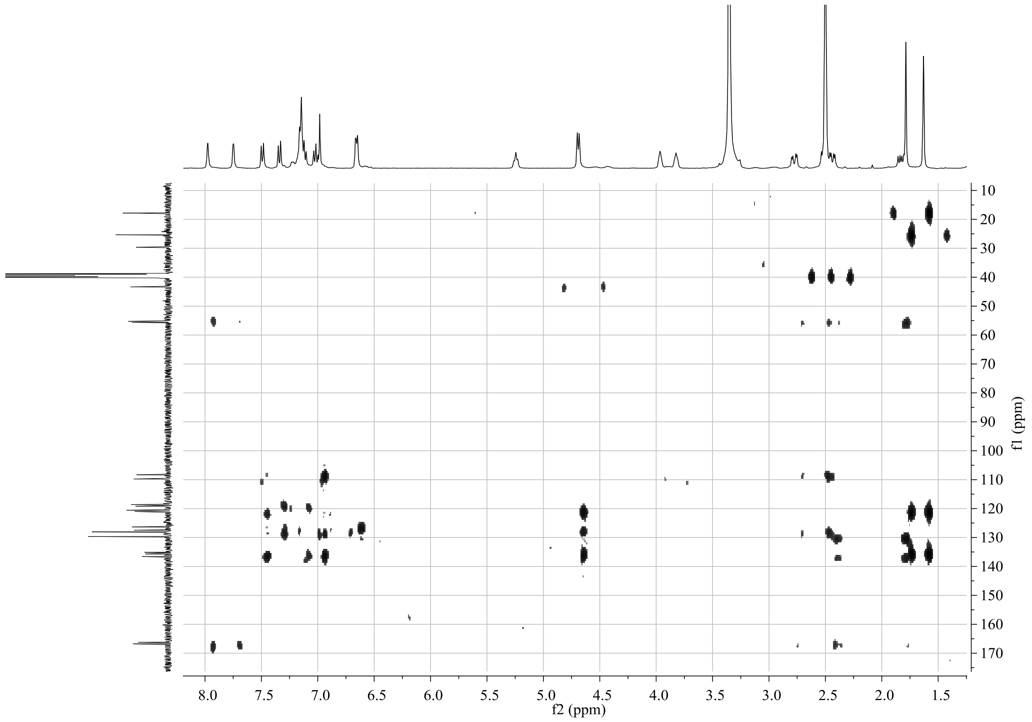

Figure SP4. J: NOESY spectrum of **4** in DMSO-d_6.

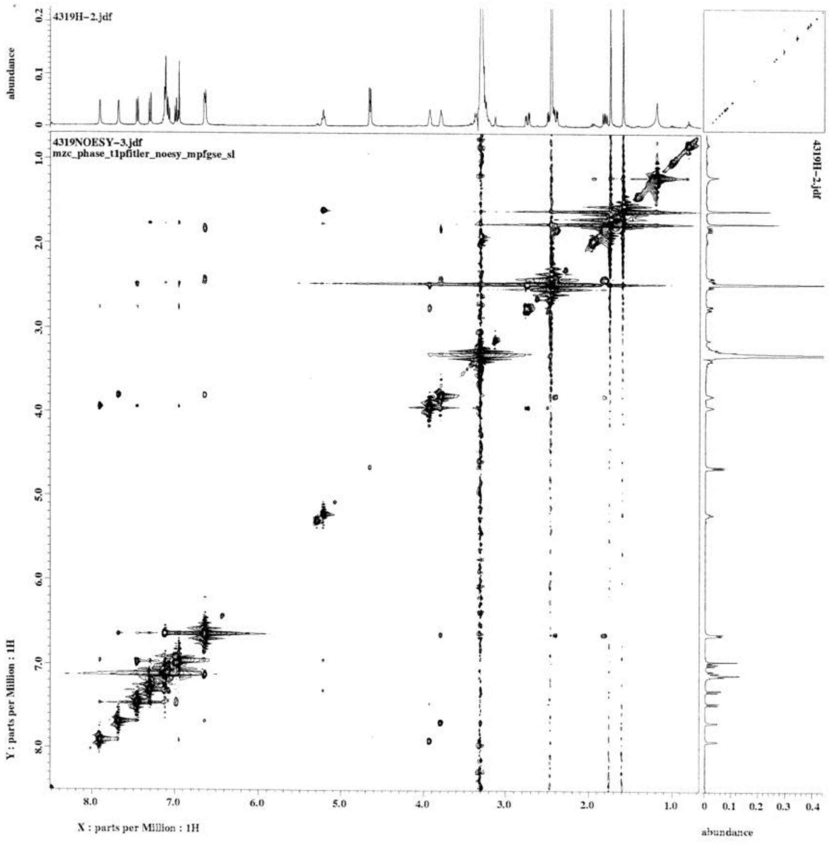

© 2014 by the authors; licensee MDPI, Basel, Switzerland. This article is an open access article distributed under the terms and conditions of the Creative Commons Attribution license (http://creativecommons.org/licenses/by/3.0/).
